# Supplementary material for: Understanding the sulphur-oxygen exchange process of metal sulphides prior to oxygen evolution reaction
Source: Nat Commun. 2023 Apr 7;14:1949. doi: 10.1038/s41467-023-37751-y (PMC10082196; doi:10.1038/s41467-023-37751-y)
Supplement: Supplementary file 1 — Supplementary Information [file 41467_2023_37751_MOESM1_ESM.pdf]

Supplementary Information

Understanding the Sulphur-Oxygen Exchange Process of Metal Sulphides prior to Oxygen Evolution Reaction

Yang Hu[1, 2, †], Yao Zheng[3, †], Jing Jin[1, †], Yantao Wang[1], Yong Peng[4, 5, *], Jie Yin[1, 2], Wei Shen[1], Yichao Hou[1], Liu Zhu[4, 5], Li An[1, 2], Min Lu[1, 2], Pinxian Xi[1, 2, *], and Chun-Hua Yan[1, 2, 6]

[1]State Key Laboratory of Applied Organic Chemistry, College of Chemistry and Chemical Engineering, Lanzhou University, Lanzhou 730000, China.

[2]Frontiers Science Center for Rare Isotopes, Lanzhou University, Lanzhou 730000, China.

[3]School of Chemical Engineering and Advanced Materials, The University of Adelaide, Adelaide, South Australia 5005, Australia.

[4]School of Materials and Energy, Lanzhou University, Lanzhou 730000, China.

[5]Electron Microscopy Centre, Lanzhou University, Lanzhou 730000, China.

[6]Beijing National Laboratory for Molecular Sciences, State Key Laboratory of Rare Earth Materials Chemistry and Applications, PKU-HKU Joint Laboratory in Rare Earth Materials and Bioinorganic Chemistry, College of Chemistry and Molecular Engineering, Peking University, Beijing 100871, China.

[†]These authors contributed equally: Yang Hu, Yao Zheng, Jing Jin.

*e-mail: pengy@lzu.edu.cn; xipx@lzu.edu.cn

Contents

Experimental section…………………………………………………………………………..3

Supplementary Figures (Supplementary Figure 1-45)…………………………………………...5

Supplementary Tables (Supplementary Table 1-7)……………………………………….…....67

References…………………………………………………………………………………75

Experimental section

Materials

Nickel nitrate hexahydrate ($Ni(NO_3)_2 \cdot 6H_2O$, 98.0%), cobalt (II) nitrate hexahydrate ($Co(NO_3)_2 \cdot 6H_2O$, 99.0%), urea (99.0%), potassium hydroxide (90.0%), and sulfuric acid (95.0%) were purchased from Aladdin. Rod GCEs were from the Chen Hua Co. Ltd. (Shanghai, China). The deionized (DI) water for solution preparation was from a Millipore Autopure system (18.2 MΩ, Millipore Ltd, USA). The 0.1 M KOH was employed as supporting electrolytes in the electrochemical experiment. Nafion 117 (brand: Dupont) was purchased from Shanghai Hesen Electrical Co., Ltd. All the other chemicals and reagents for electrochemical measurements were of analytical grade and used as received.

Synthesis of $NiCo_2O_4$ nanocrystals

A mixture of 0.33g $Ni(NO_3)_2 \cdot 6H_2O$, 0.665g $Co(NO_3)_2 \cdot 6H_2O$, and 0.24g urea were added into 80ml distilled water stirring for 30 minutes. After that the solution was transferred into an 100 mL Teflon-lined stainless steel autoclave and maintained at 120 °C for 16 hours. After the reaction, the products were centrifuged at 8000 rpm for 5 min and washed with DI water and ethanol. The hybrid intermediate was dried at 60 °C overnight and annealed at 300 °C with a rate of 2 °C/min in air for 2 h to get the $NiCo_2O_4$ nanocrystals.

Physicochemical Characterizations

XRD measurements were carried out on Rigaku MiniFlex 600 diffractometer with Cu Kα radiation (λ = 0.1542 nm) from 10° to 80° under a constant voltage of 40 kV. The atomic-scale crystal structure, elemental mapping and EELS spectra of as-prepared bi-metal sulphides were characterised by an aberration-corrected scanning transmission electron microscope (FEI Titan Cubed Themis G2 300, FEI, USA) operated at 300 kV and equipped with a monochromator, Gatan image filter (GIF Quantum ER/965, Gatan, USA) and energy dispersive X-Ray spectroscopy (EDX, Bruker, USA). Synchrotron radiation X-ray absorption fine structure (XAFS) spectroscopy analyses of Co and Ni K-edge were performed in transmission mode at the Singapore Synchrotron Light Source XAFCA beamline.

Differential electrochemical mass spectroscopy (DEMS) measurements

DEMS measurements were carried out to determine the ^{18}O-labeled volatile reaction products of as-prepared bi-metal sulphides during OER process using a QAS 100 device (Linglu Instruments,

Shanghai). A saturated Ag/AgCl electrode and a Pt wire were used as reference electrode and counter electrode, respectively. The working electrodes were prepared by sputtering Au onto 50 μm thick porus PTFE films. Then, the catalysts were drop cast onto the Au with a loading mass of 0.65 mg cm^{-2}. First, the catalysts were labeled with ^{18}O isotopes by performing 5 CV cycles at a scan rate of 5 mV/s in ^{18}O-labed 1M KOH. Afterwards, ^{18}O-labeled electrodes were rinsed with ^{16}O water for five times to remove the remaining $H_2{}^{18}O$. Finally, the electrodes were carried out CV cycles in ^{16}O KOH solution at the above potential window and scan rate. At the meantime, gas products of different molecular weights generated during OER process were measured in real time by mass spectroscopy. Since catalysts were thoroughly rinsed with ^{16}O water after ^{18}O-labelling, it is unlikely that ^{18}O species adsorbed on the surface contribute substantially to the observed $^{34}O_2$ ($^{16}O^{18}O$) signals. Thus, it can be determined to investigate the participation of lattice oxygen from catalysts in OER by measuring the $^{34}O_2$ signals.

Computational Details

Density functional theory (DFT) calculations were performed by employing the Vienna ab initio simulation package (VASP)[1, 2]. The Perdew-Burk-Ernzerhof (PBE) within the generalized gradient approximation (GGA) was used to describe the exchange-correlation functional[3]. The electron-ion potential was described by the projected augmented wave method (PAW)[4], and a plane-wave energy cut-off of 450 eV was set in this study. The convergence criterion of the force was -0.03 eV/Å and the convergence thresholds for the total energy was 10^{-5} eV. The Brillouin-zone integration was approximated 5×3×1 by grid using the Monkhorst-Pack k-point mesh. A vacuum space of 15 Å in the Z-direction was chosen to avoid artificial interaction. The effect of van der Waals (vdW) interactions was included for weak interaction cases using the semiempirical correction scheme of Becke-Jonson damping, DFT-D3[5].

Density functional theory (DFT) calculations were employed to investigate the adsorption energies of OH* on the (110) crystal facets of $(NiCo)S_{1.33}$ and O-substituted $(NiCo)S_{1.33}$, respectively. The adsorption energy of OH* (E_{ads}) on the catalyst surface was calculated by Eq. (1)

$$E_{ads} = E_{total} - E_{slab} - E_{OH*} \tag{1}$$

in which E_{total} is the total energy of the catalyst surface with adsorbed OH*, E_{slab} is the electronic energy of clean surface, and E_{OH*} is the electronic energy of free hydroxyl ion calculated by Eq. (2)

$$E_{OH*} = E_{H2O} - 1/2E_{H2} \tag{2}$$

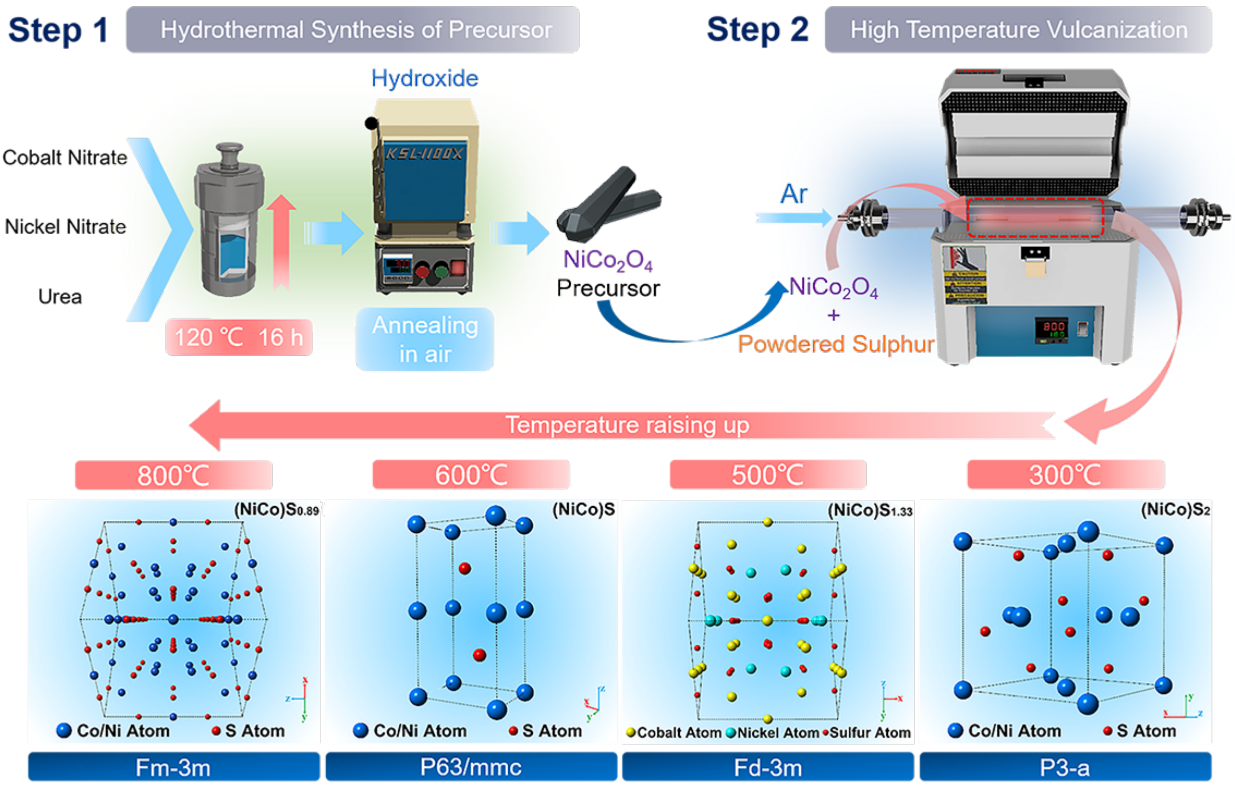

Supplementary Fig. 1 | Materials synthesis. Schematic diagram of the synthesis strategy for cobalt-nickel bimetallic sulphides.

Based on the two-steps synthesis strategy, we precisely regulated the sulfidation degree of the $NiCo_2O_4$ precursor by adjusting the evaporation rate of sulphur powder based on temperature control, and achieved a series of cobalt-nickel bimetallic sulphides. Furthermore, the relationship between the vulcanization temperature and the structure of as-prapared sulphides indicated that the content of S in the bimetallic sulphide increases with the decrease of temperature, implying a change of the metal-sulphur coordination forms, which further induces the formation of different crystal structures.

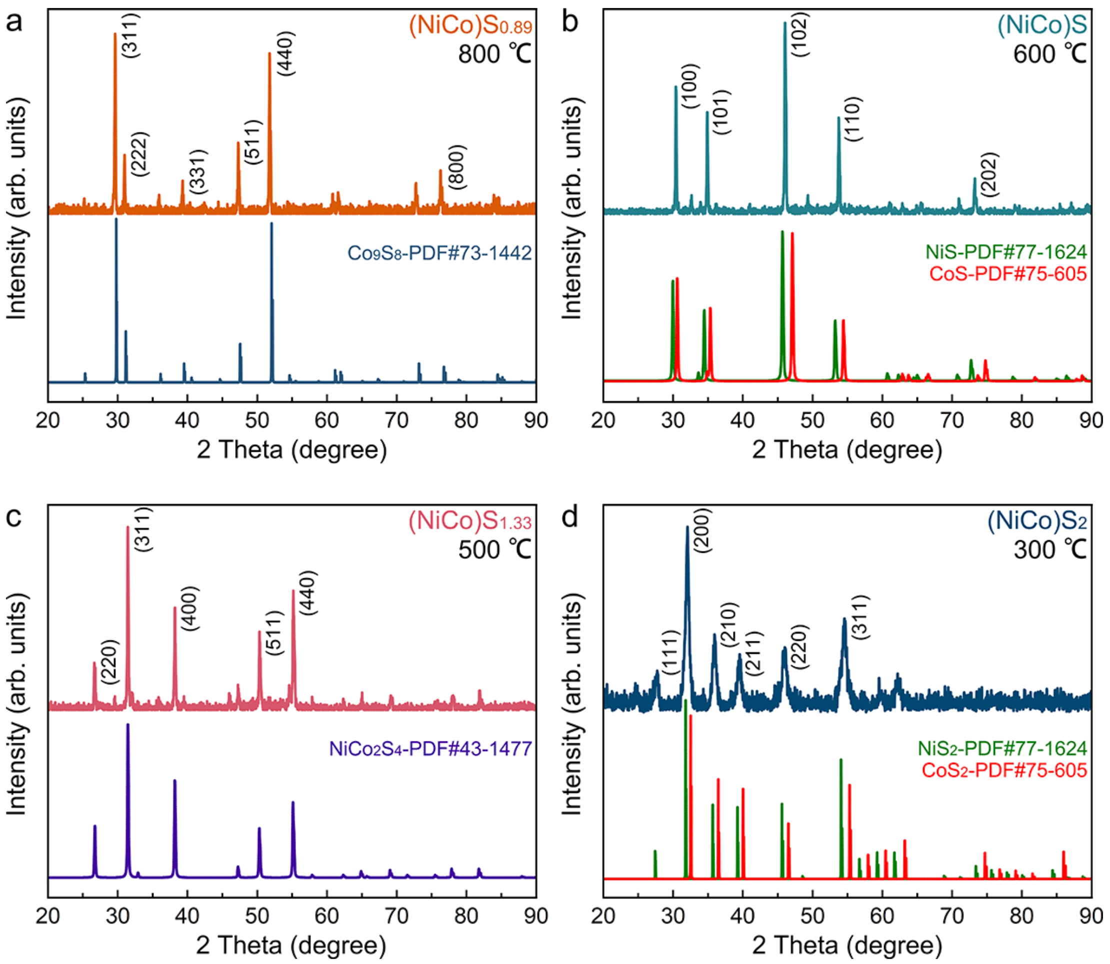

Supplementary Fig. 2 | Structure characterization. a-d, The XRD spectra and corresponding standard PDF card of $(NiCo)S_{0.89}$, $(NiCo)S$, $(NiCo)S_{1.33}$ and $(NiCo)S_2$, respectively.

As the vulacanizing temperature was controlled at 800 °C, the diffraction peak of the sample is consistent with the standard peak of Co_9S_8 (Supplementary Fig. 2a) with the peak position shifting to a lower angle. Similarly, for those samples at the temperature of 600 °C (Supplementary Fig. 2b) and 300 °C (Supplementary Fig. 2d), the characteristic peaks are both in the middle of the two monometallic sulphides. This kind of a peak shift could be attributed to the different ionic radii of the Ni and Co cations, which causes the changes of lattice parameters, and suggests a solid solution property. Notably, the sample under a vulacanizing temperature of 500 °C perfectly matches the structure of $NiCo_2S_4$ (Supplementary Fig. 2c), which is a standard antispinel sulphide. As observed in XRD parttens, it can be confirmed that we have successfully synthesized four different kinds of nickel-cobalt bimetallic sulphides, which are $(NiCo)S_{0.89}$, $(NiCo)S$, $(NiCo)S_{1.33}$ and $(NiCo)S_2$. Furthermore, the relationship between the vulcanization temperature and the structure of as-prapared sulphides implies a change of the metal-sulfur coordination forms, which further induces the formation of different crystal structures.

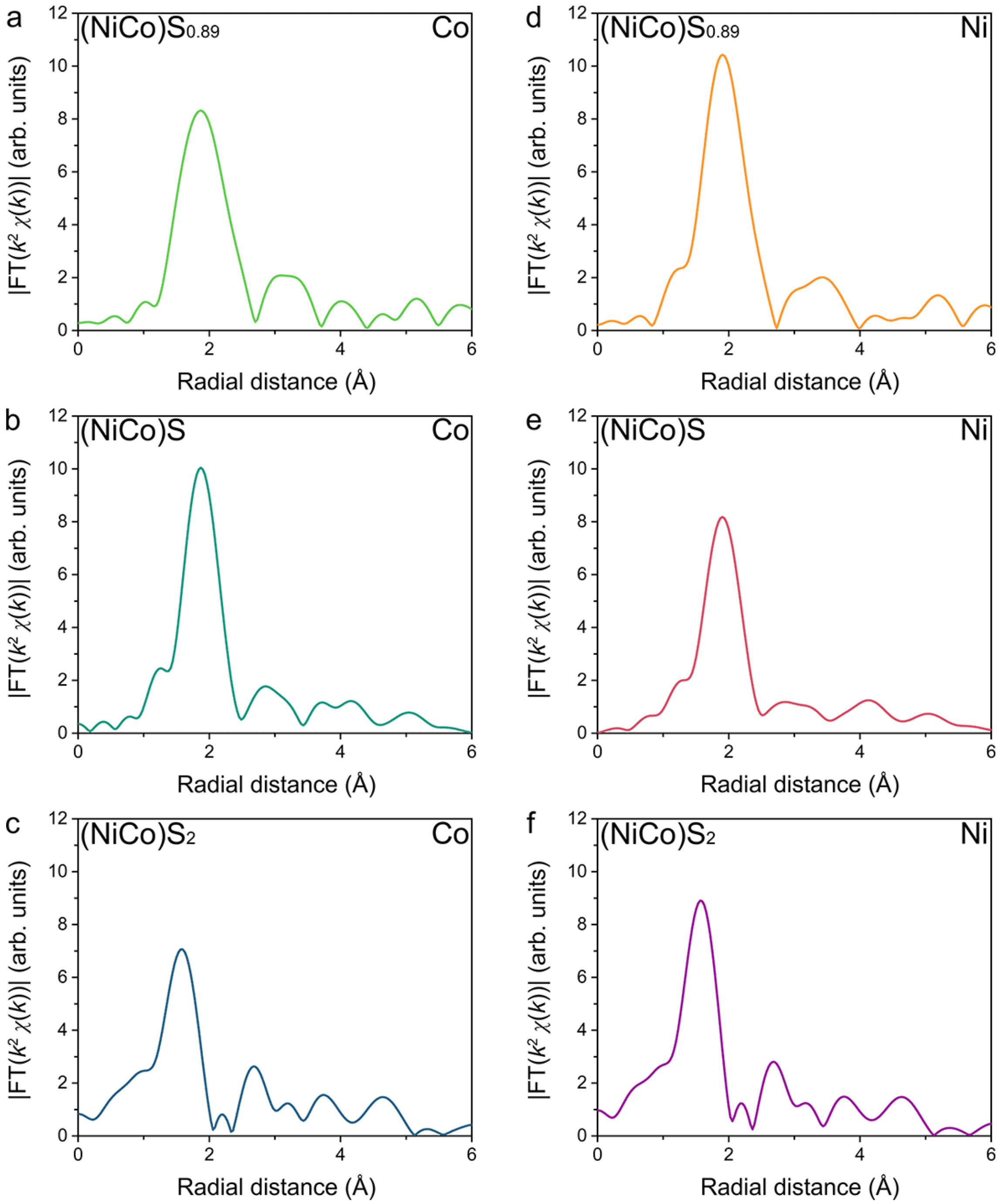

Supplementary Fig. 3 | FT K-edge EXAFS spectra of $(NiCo)S_{0.89}$, (NiCo)S, and $(NiCo)S_2$. a-c, FT K-edge EXAFS spectra of Co. **d-f**, FT K-edge EXAFS spectra of Ni.

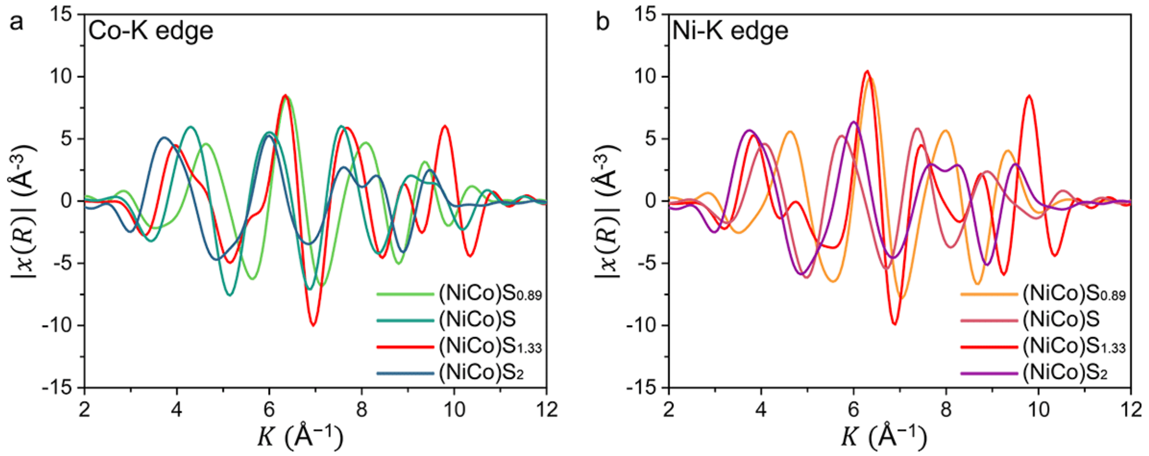

Supplementary Fig. 4 | Co and Ni K-edge EXAFS spectra of as-prepared nickel-cobalt bimetallic sulphides (x ≈ 0.1–2.0). a, Co K-edge EXAFS spectra. **b**, Ni K-edge EXAFS spectra.

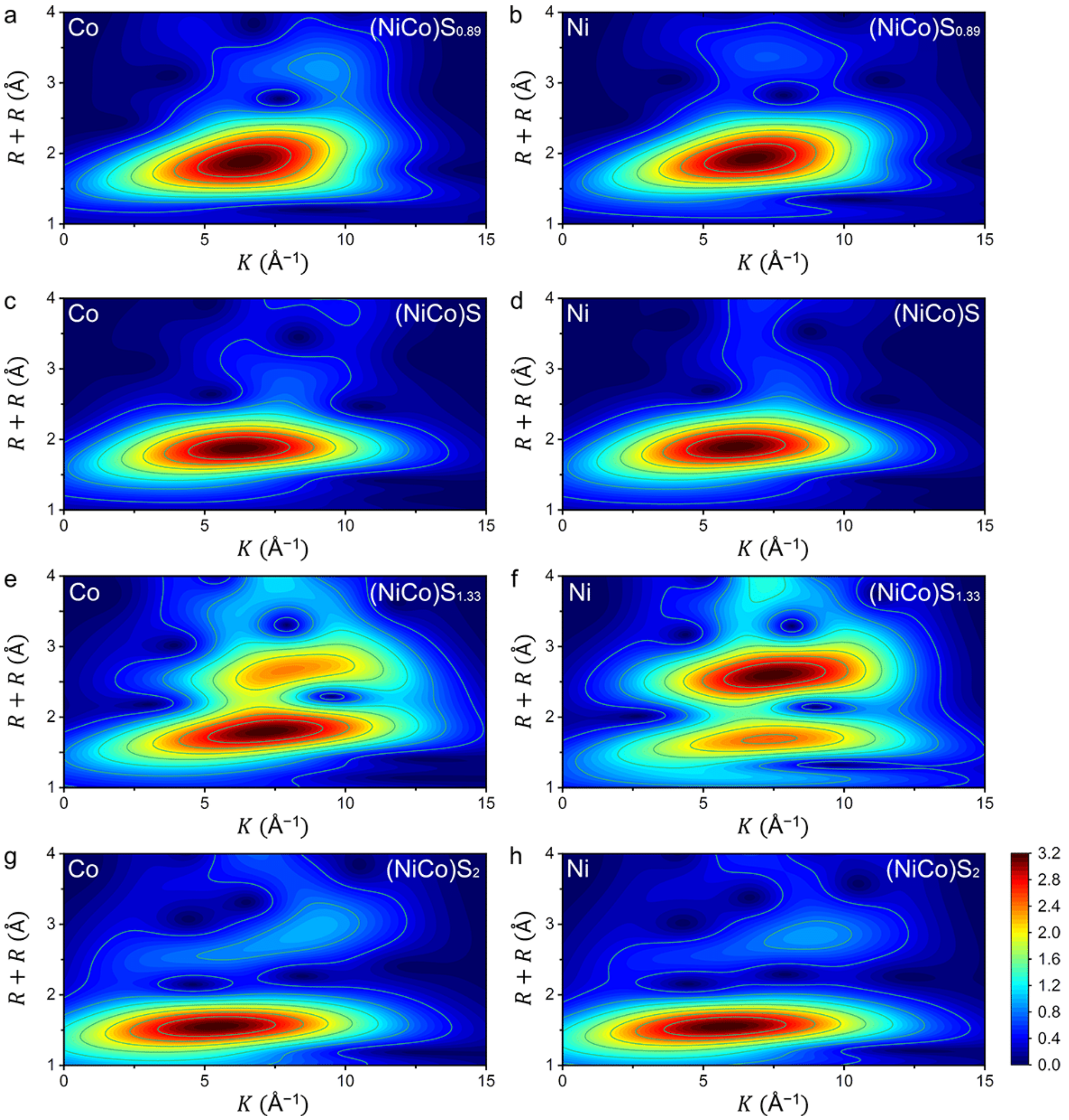

Supplementary Fig. 5 | Comparison of Co and Ni K-edge WT-EXAFS. a-b, Wavelet transforms for the k^3-weighted Co and Ni K-edge EXAFS signals of $(NiCo)S_{0.89}$. **c-d**, Wavelet transforms for the k^3-weighted Co and Ni K-edge EXAFS signals of (NiCo)S. **e-f**, Wavelet transforms for the k^3-weighted Co and Ni K-edge EXAFS signals of $(NiCo)S_{1.33}$. **g-h**, Wavelet transforms for the k^3-weighted Co and Ni K-edge EXAFS signals of $(NiCo)S_2$.

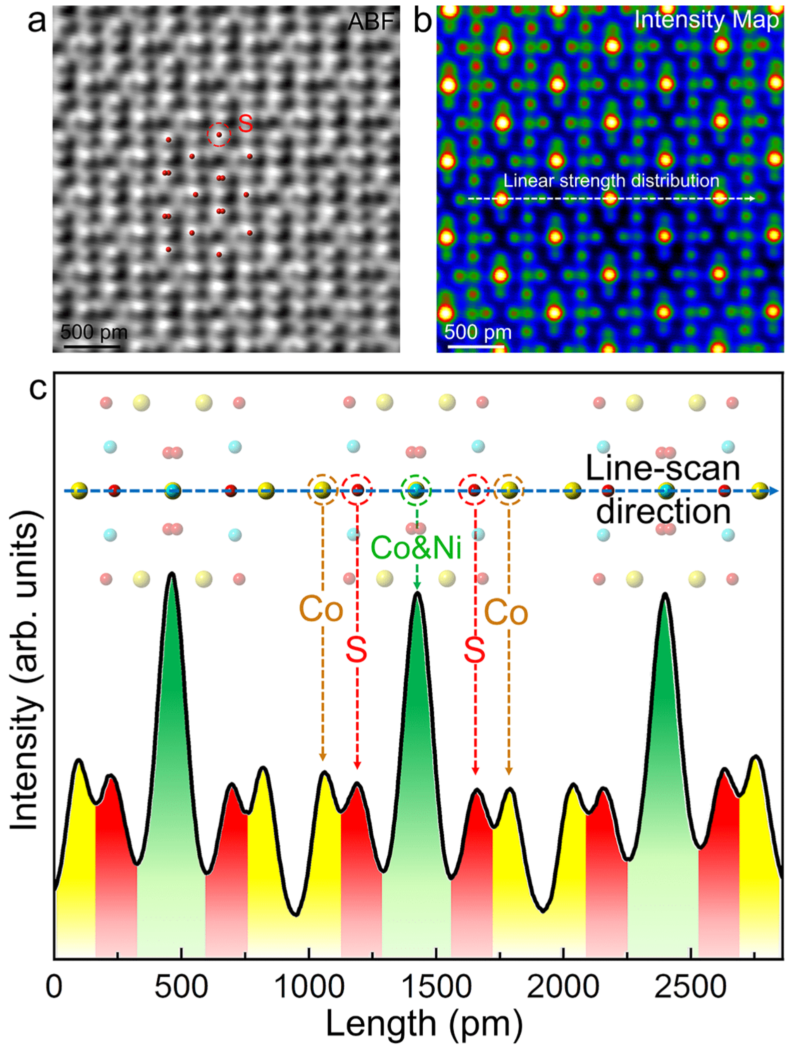

Supplementary Fig. 6 | The occupation analysis of metal and sulphur atoms. a, ABF image of $(NiCo)S_{1.33}$. **b-c**, Linear strength analysis of high-angle annular dark field (HAADF) image.

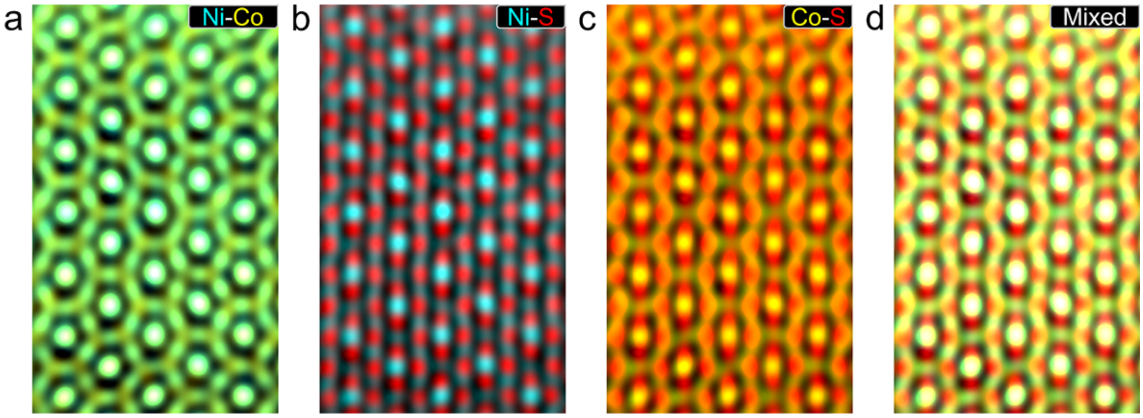

Supplementary Fig. 7 | Metal-sulfur mixed EDX mapping of (NiCo)$S_{1.33}$. a, Ni and Co mixed EDX mapping. **b**, Ni and S mixed EDX mapping. **c**, Co and S mixed EDX mapping. **d**, Co, Ni and S mixed EDX mapping.

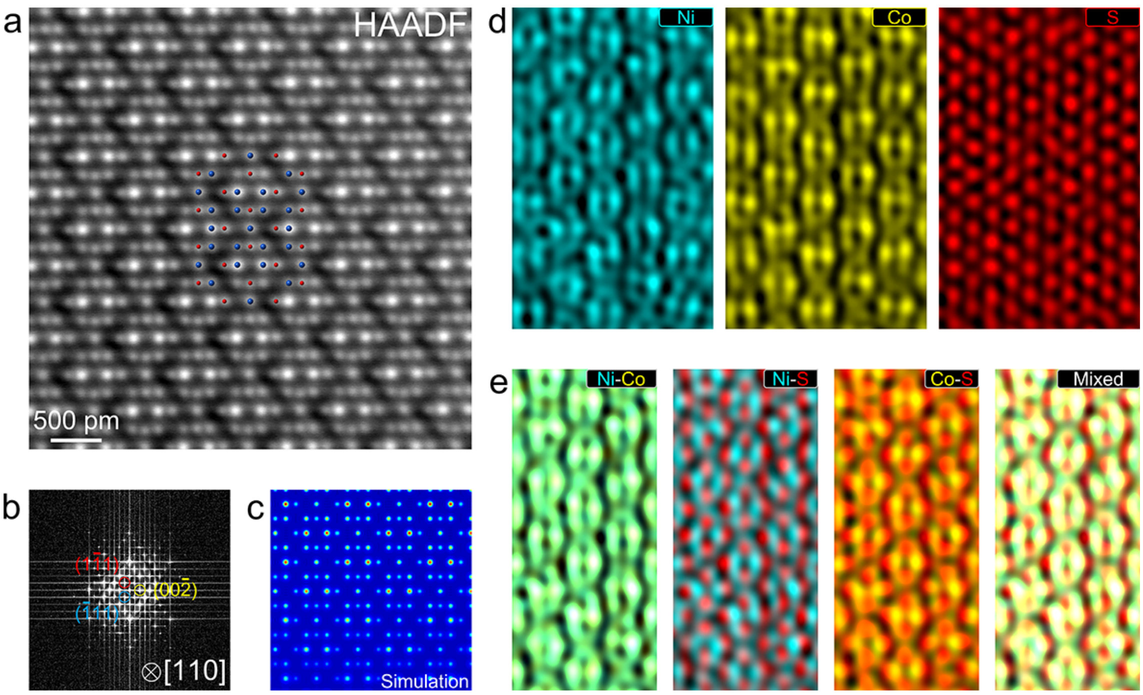

Supplementary Fig. 8 | Structural characterization of (NiCo)$S_{0.89}$. **a**, Atomic STEM-HAADF image of (NiCo)$S_{0.89}$. **b**, Corresponding FFT pattern. **c**, HAADF simulation of (NiCo)$S_{0.89}$ along the [110] orientation. **d-e**, Atomic EDX elemental mapping of Ni, Co and S.

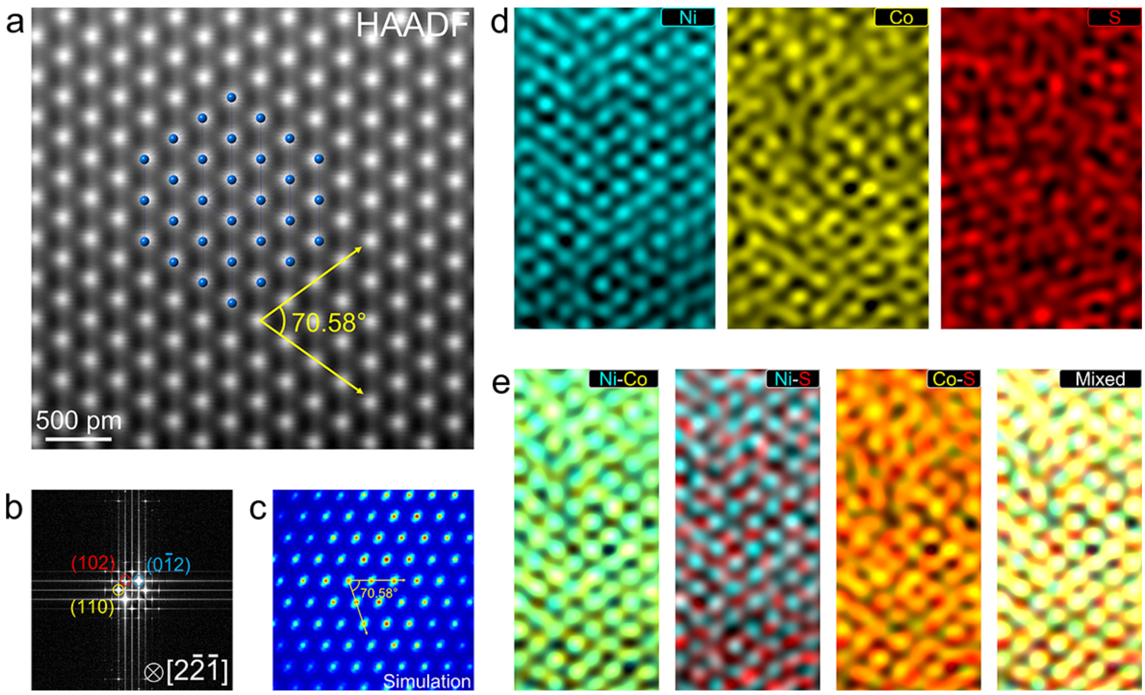

Supplementary Fig. 9 | Structural characterization of (NiCo)S. a, Atomic STEM-HAADF image of (NiCo)S. **b**, Corresponding FFT pattern. **c**, HAADF simulation of (NiCo)S along the [2-2-1] orientation. **d-e**, Atomic EDX elemental mapping of Ni, Co and S.

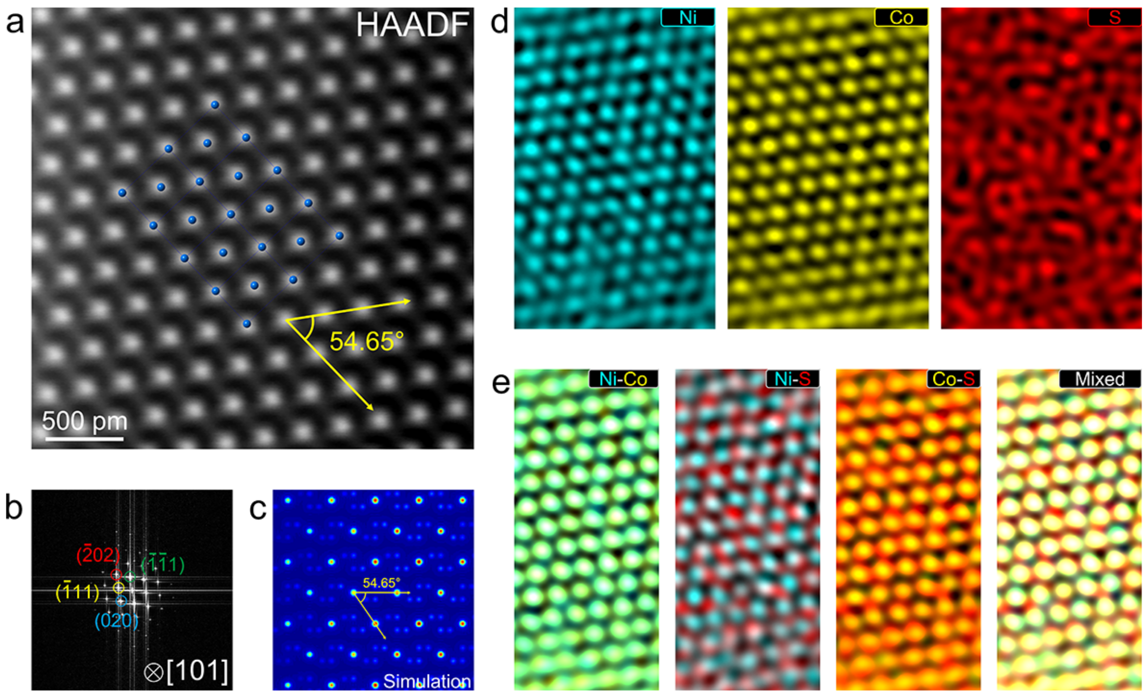

Supplementary Fig. 10 | Structural characterization of (NiCo)S_2. a, Atomic STEM-HAADF image of (NiCo)S_2. **b**, Corresponding FFT pattern. **c**, HAADF simulation of (NiCo)S_2 along the [101] orientation. **d-e**, Atomic EDX elemental mapping of Ni, Co and S.

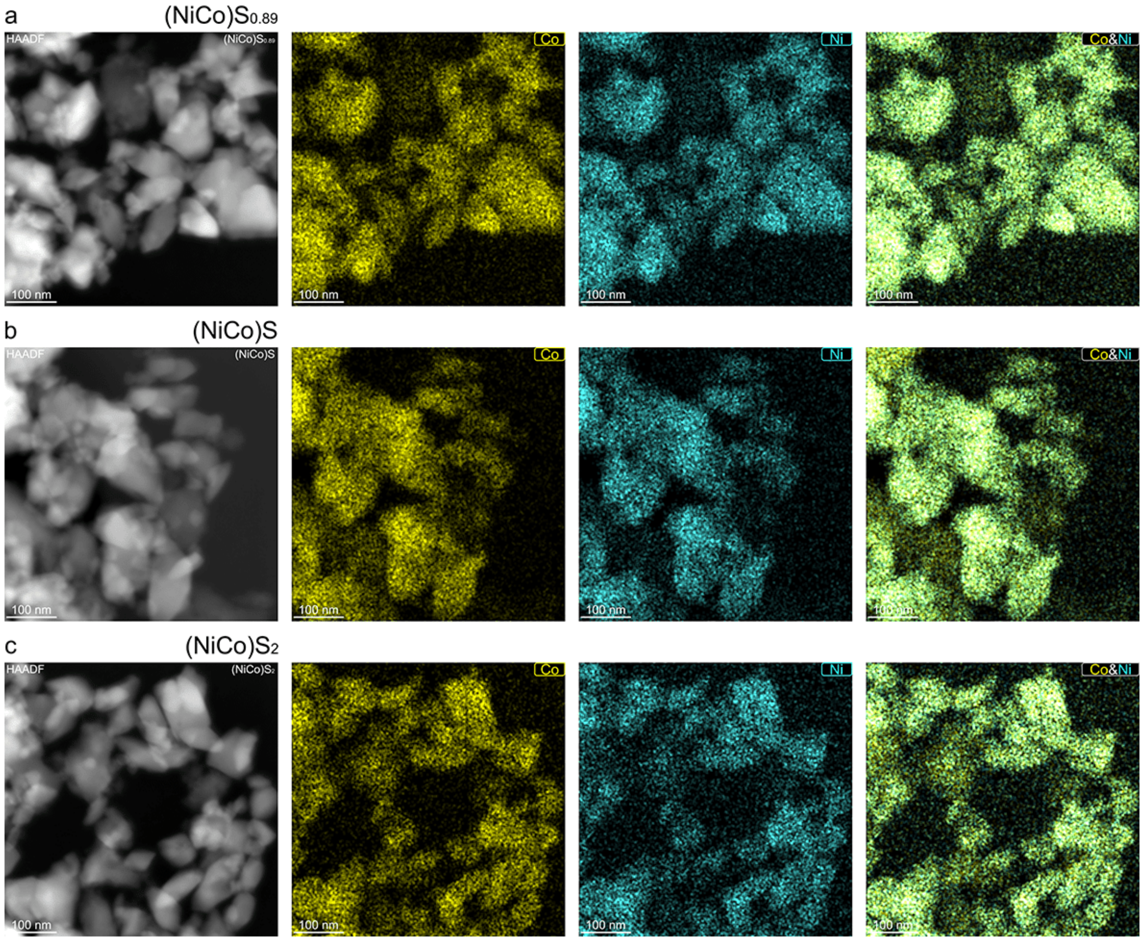

Supplementary Fig. 11 | Elemental mapping of Co and Ni in as-prepared nickel-cobalt bimetallic sulphides at low magnification. a, Elemental mapping of Co and Ni in $(NiCo)S_{0.89}$. **b**, Elemental mapping of Co and Ni in (NiCo)S. **c**, Elemental mapping of Co and Ni in $(NiCo)S_2$.

The Co and Ni are evenly ditributed in each particle of $(NiCo)S_{0.89}$, (NiCo)S and $(NiCo)S_2$. But for mixture of binaries, the elemental distribution of Co and Ni should exhibit a clear distinction because each single particle has only one kind of metallic element. Thus, it could be confirmed that the $(NiCo)S_{0.89}$, (NiCo)S and $(NiCo)S_2$ reported in our manuscript are dual-transition metal sulphides.

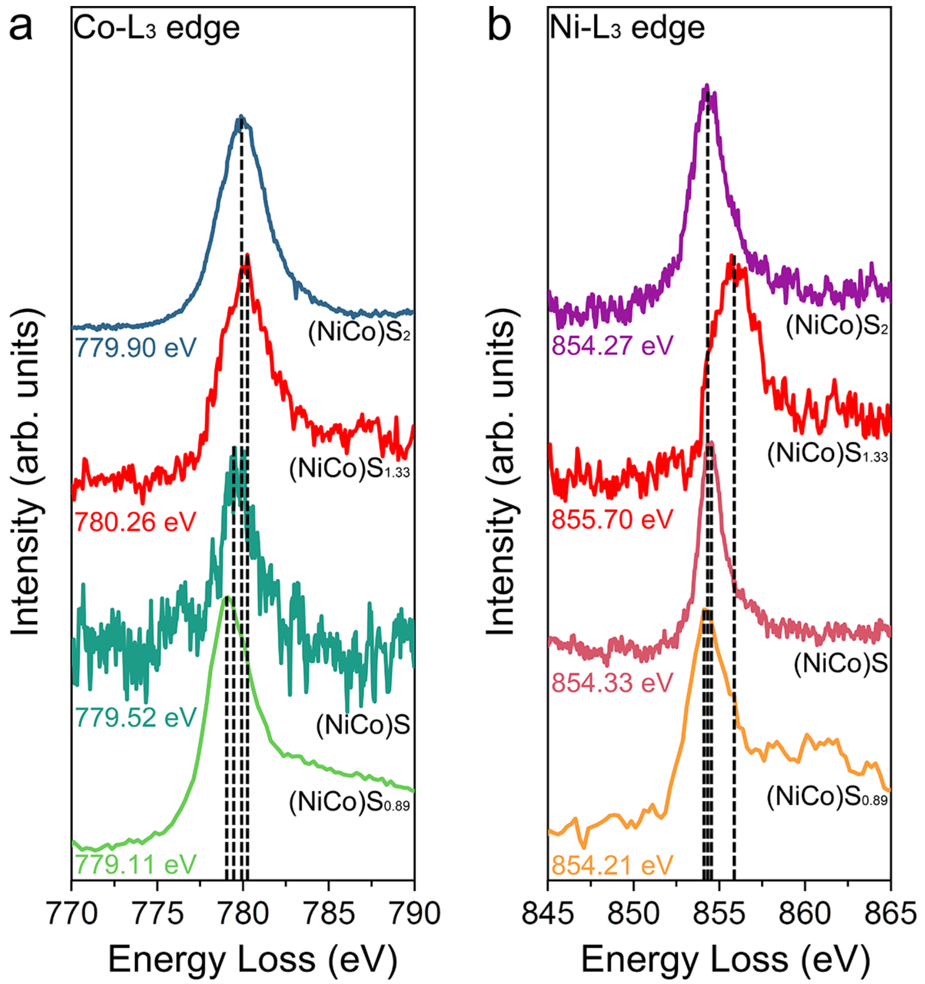

Supplementary Fig. 12 | The EELS spectra of Co- and Ni-L_3 edges in as-prepared nickel-cobalt bimetallic sulphides. a, Co-L_3 edge. **b**, Ni-L_3 edge.

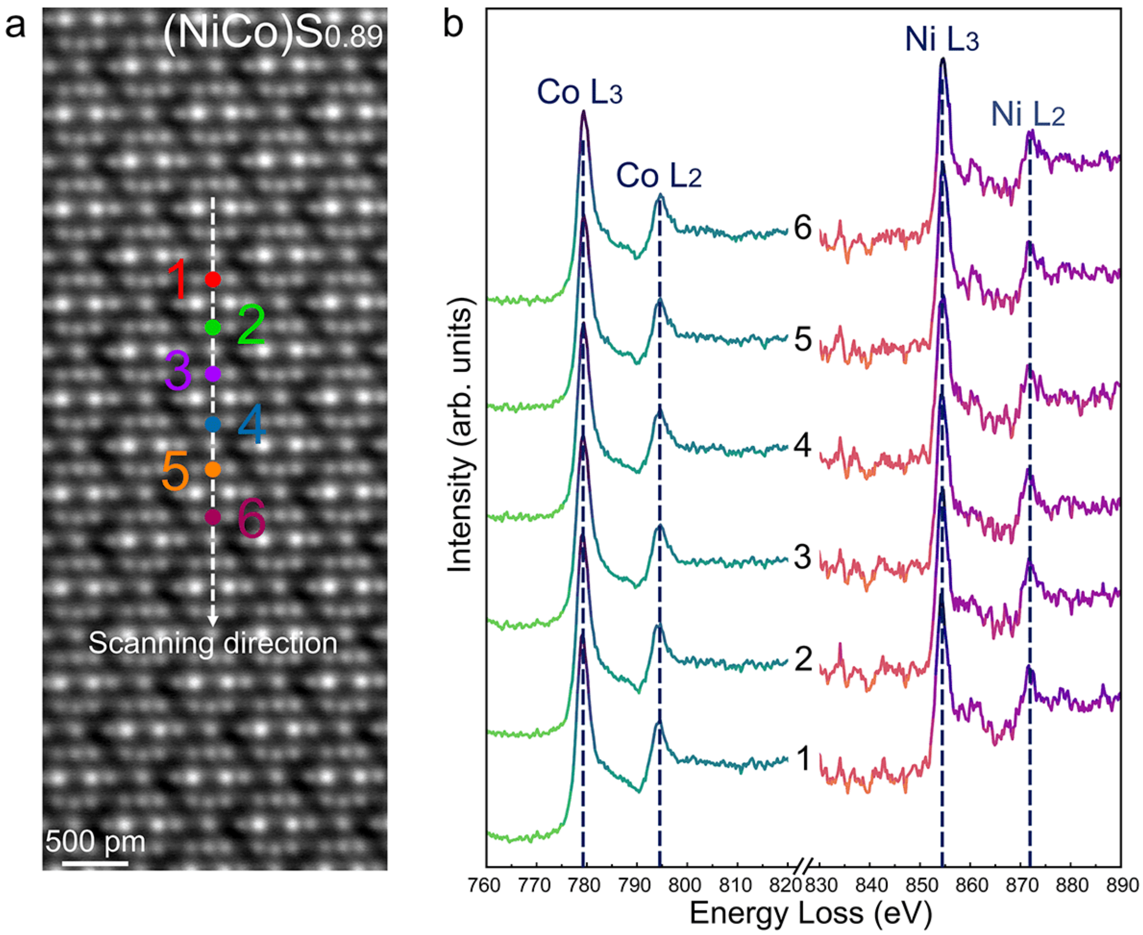

Supplementary Fig. 13 | Atomic EELS analysis of $(NiCo)S_{0.89}$ via line-scan mode. a, Atomic HAADF image of $(NiCo)S_{0.89}$ and corresponding EELS signal acquisition sites. **b**, EELS spectra of Co and Ni collected from various acquisition sites.

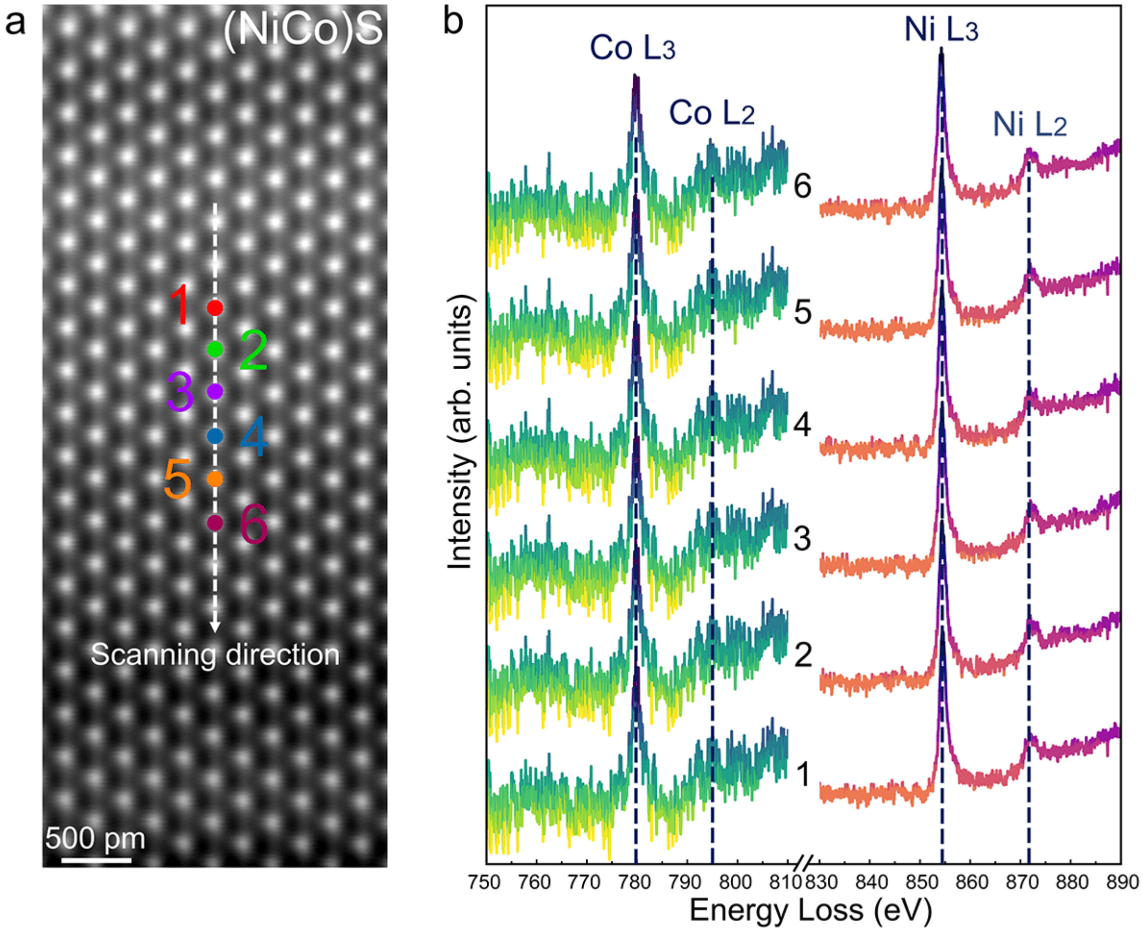

Supplementary Fig. 14 | Atomic EELS analysis of (NiCo)S via line-scan mode. a, Atomic HAADF image of (NiCo)S and corresponding EELS signal acquisition sites. **b**, EELS spectra of Co and Ni collected from various acquisition sites.

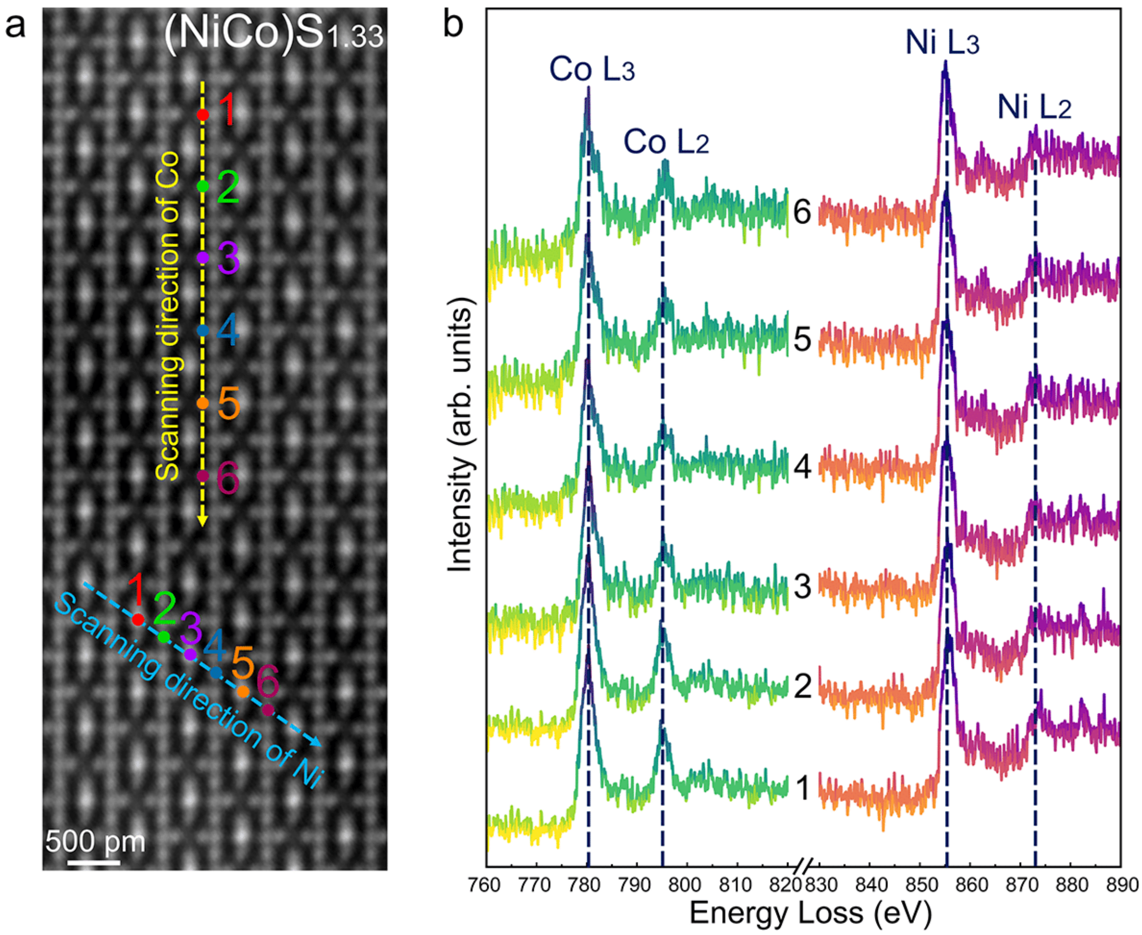

Supplementary Fig. 15 | Atomic EELS analysis of $(NiCo)S_{1.33}$ via line-scan mode. a, Atomic HAADF image of $(NiCo)S_{1.33}$ and corresponding EELS signal acquisition sites. **b**, EELS spectra of Co and Ni collected from various acquisition sites.

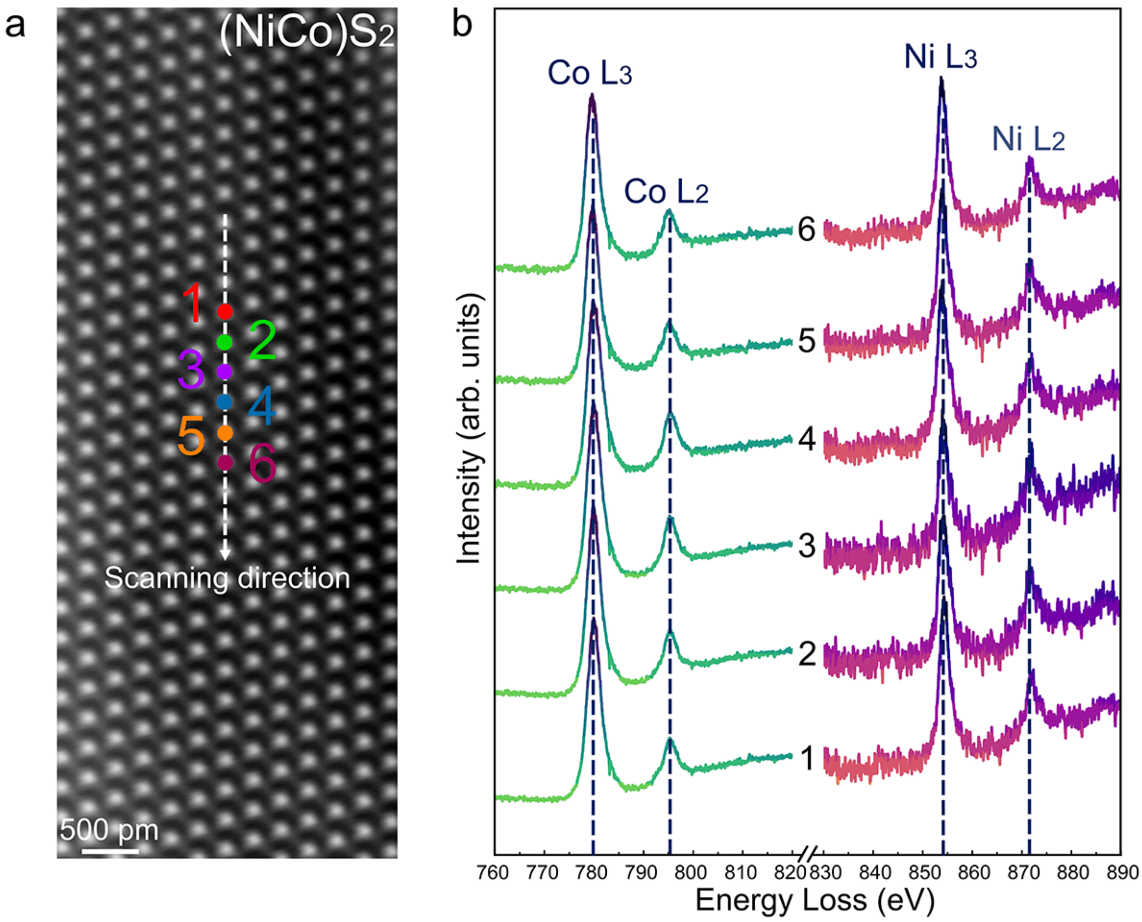

Supplementary Fig. 16 | Atomic EELS analysis of $(NiCo)S_2$ via line-scan mode. a, Atomic HAADF image of $(NiCo)S_2$ and corresponding EELS signal acquisition sites. **b**, EELS spectra of Co and Ni collected from various acquisition sites.

According to the sub-point spectrum (Supplementary Fig. 13-16), there is no shift of Co and Ni EELS spectra in each nickel-cobalt bimetallic sulphides, which proved that the metal atoms in these as-prepared sulphides have a stable and uniform local electronic environment without any changes caused by obvious atomic defects. Therefore, the shift of the metal elements EELS spectra between different sulphides can be used to reflect the intrinsic information of the material, and this local electronic environment change can be attributed to the differences of the metal-sulfur coordination forms.

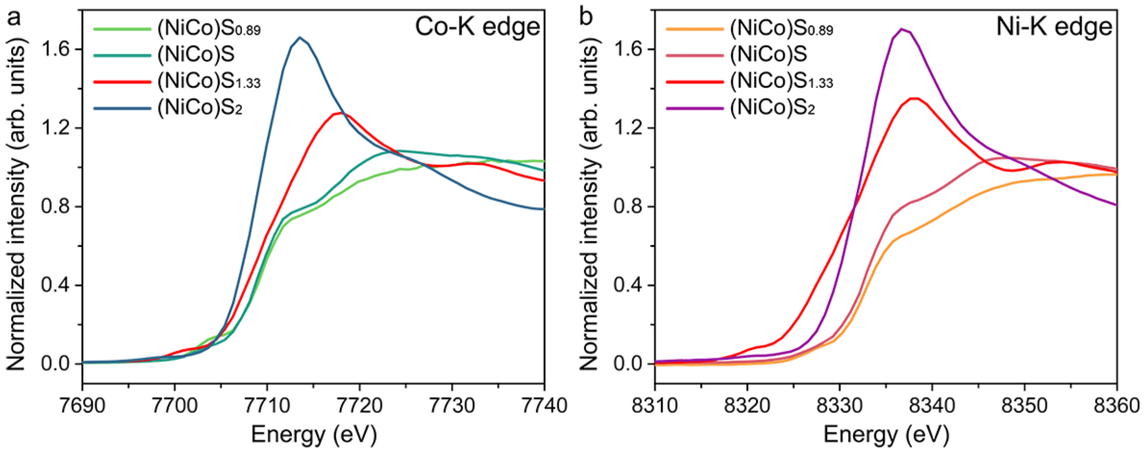

Supplementary Fig. 17 | Co and Ni K-edge EXAFS spectra of as-prepared nickel-cobalt bimetallic sulphides ($x \approx 0.1$–2.0). a, K-edge EXAFS spectra of Co. **b**, K-edge EXAFS spectra of Ni.

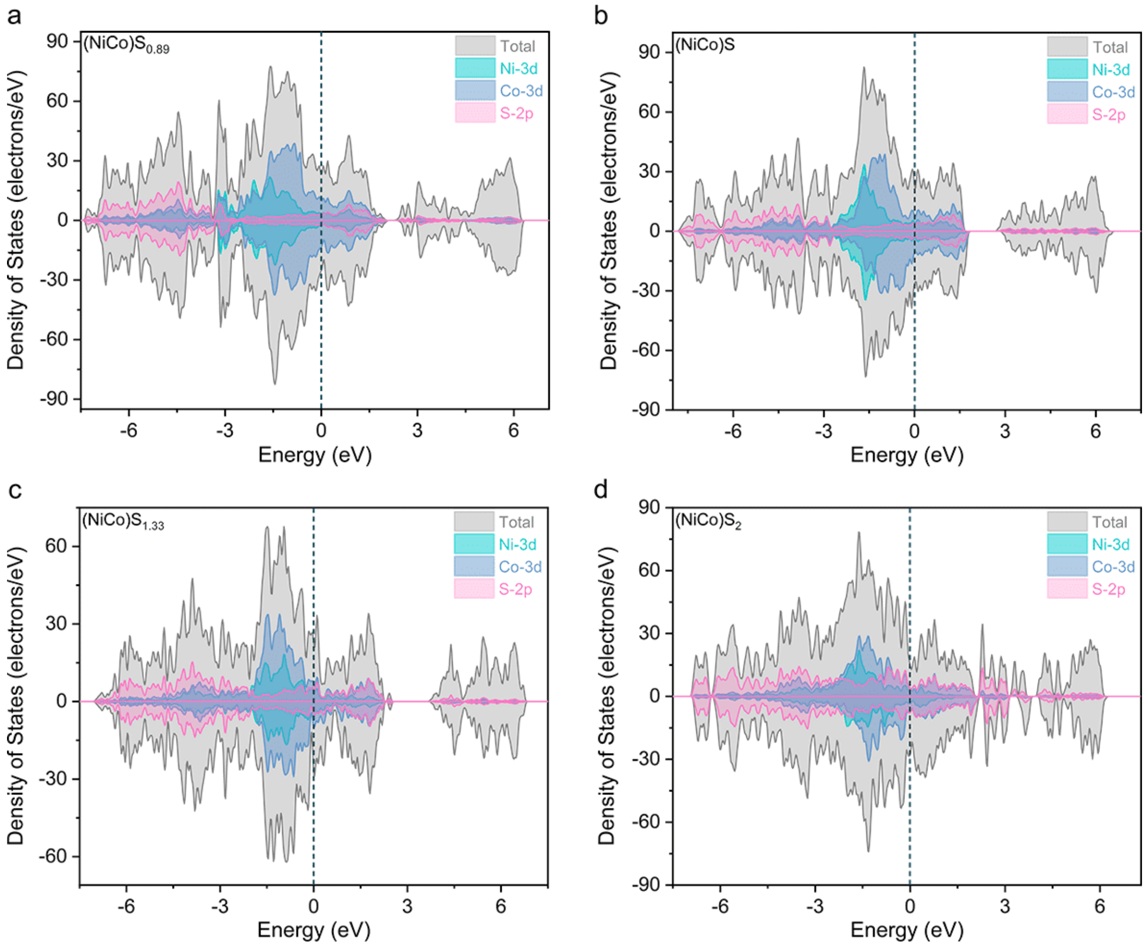

Supplementary Fig. 18 | The calculation of PDOS in different sulphides. a-d, PDOS of $(NiCo)S_{0.89}$, (NiCo)S, $(NiCo)S_{1.33}$ and $(NiCo)S_2$, respectively.

The partial density of states (PDOS) of $(NiCo)S_{0.89}$, (NiCo)S, $(NiCo)S_{1.33}$ and $(NiCo)S_2$ possess the obvious contribution near the Fermi level, suggesting the metallic properties for $(NiCo)S_{0.89}$, (NiCo)S, $(NiCo)S_{1.33}$ and $(NiCo)S_2$.

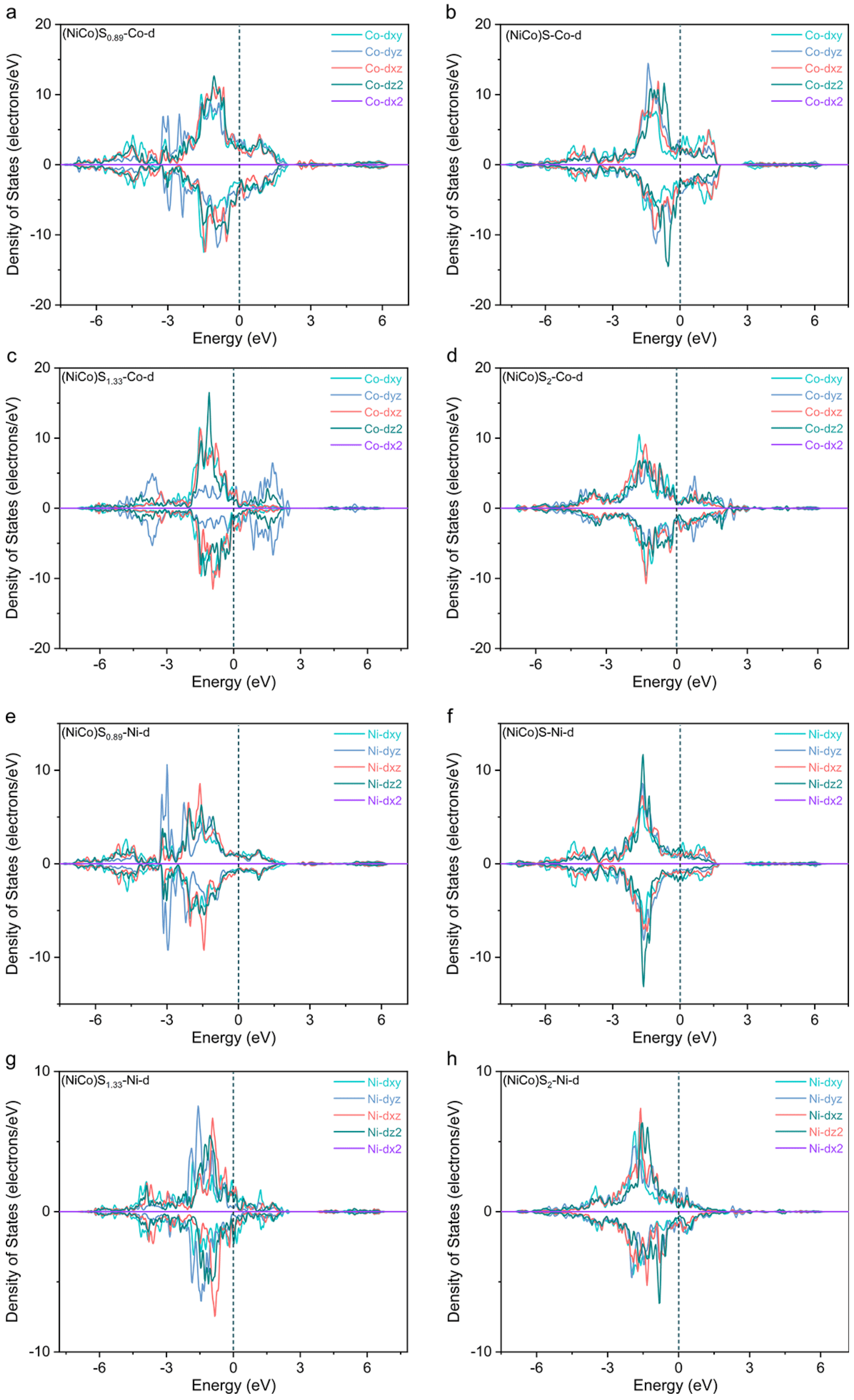

Supplementary Fig. 19 | The calculation of PDOS-Co and Ni in different sulphides. a-d, PDOS-Co of $(NiCo)S_{0.89}$, (NiCo)S, $(NiCo)S_{1.33}$ and $(NiCo)S_2$, respectively. **e-h**, PDOS-Ni of $(NiCo)S_{0.89}$, (NiCo)S, $(NiCo)S_{1.33}$ and $(NiCo)S_2$, respectively.

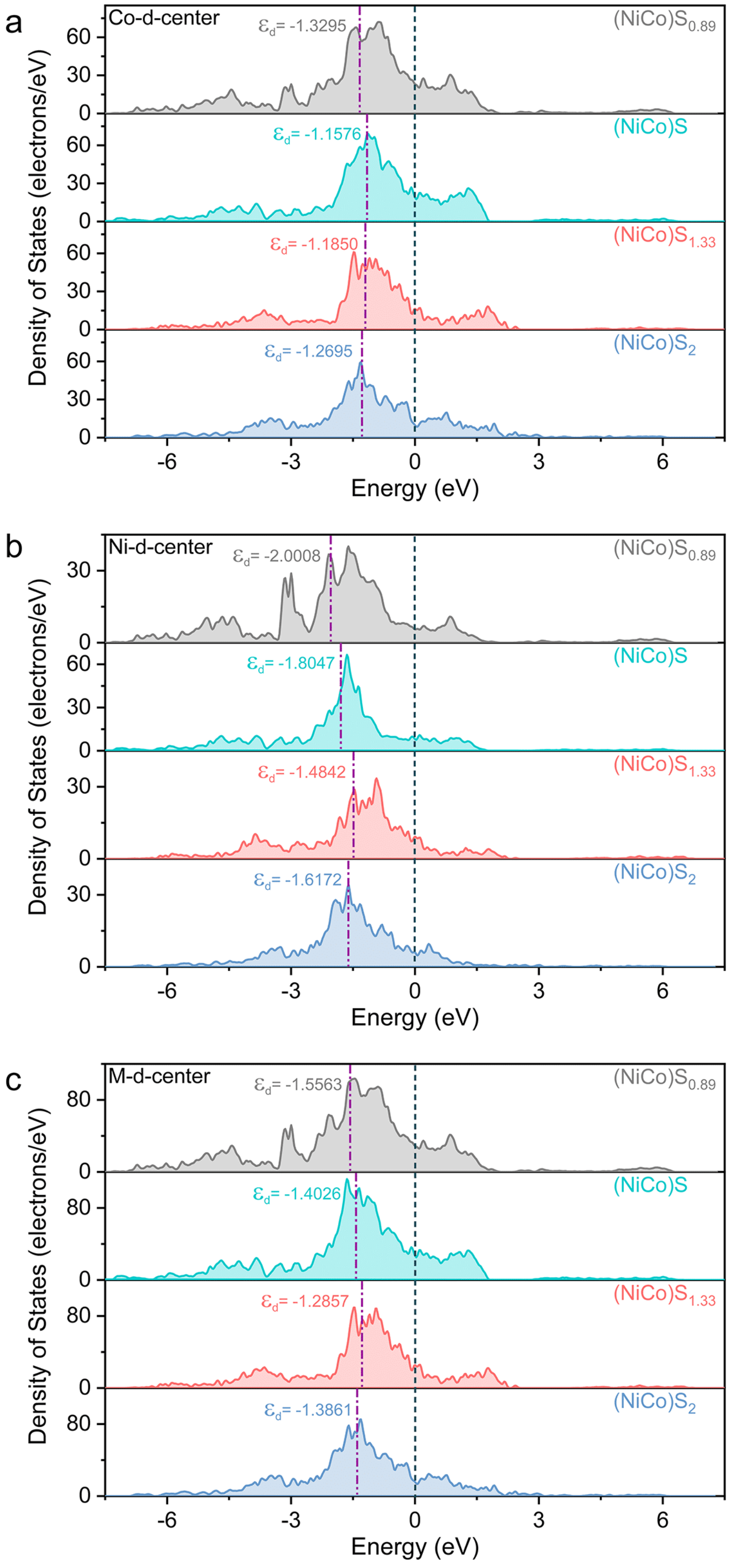

Supplementary Fig. 20 | The calculation of d-band center in different sulphides. a-c, d-band center of Co, Ni, Metal in different sulphides, respectively.

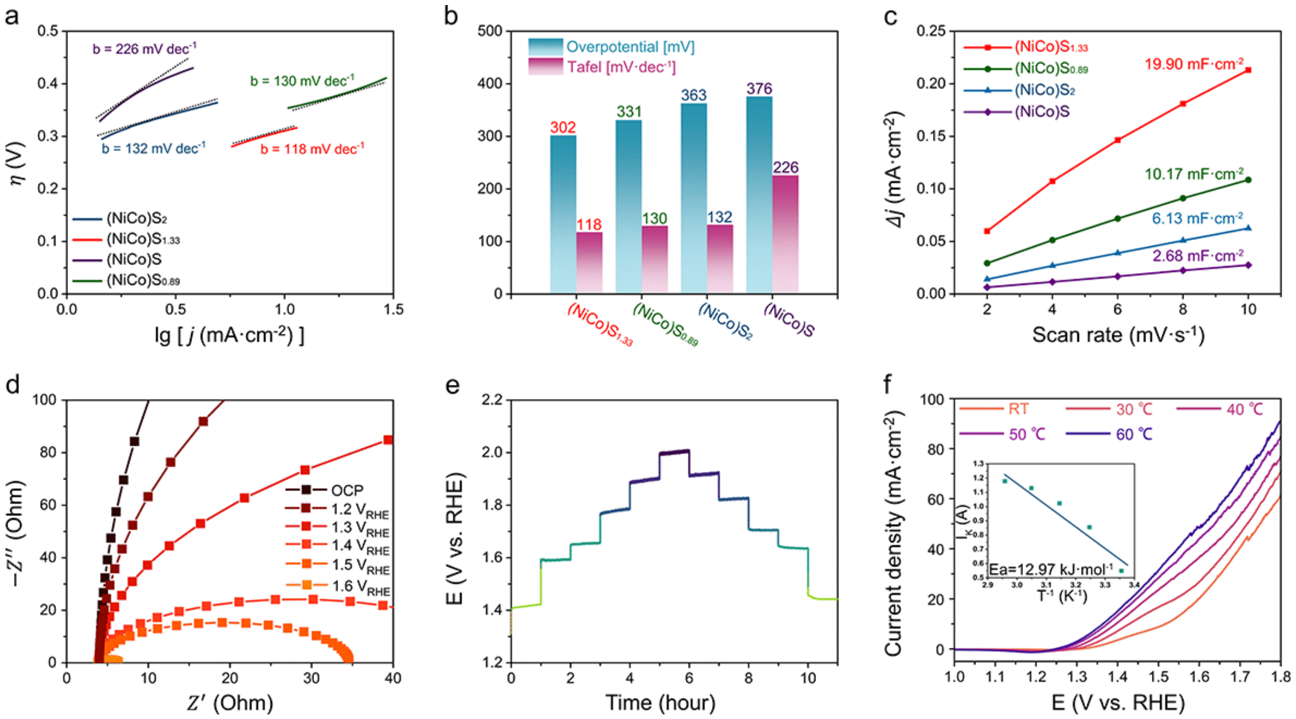

Supplementary Fig. 21 | OER performance of as-prepared nickel-cobalt bimetallic sulphides. **a**, Derived Tafel slopes. **b**, Statistics of overpotential and Tafel slope values. **c**, Plots of *Δj* versus scan rates for as-prepare bi-metal sulphides. **d**, The EIS of $(NiCo)S_{1.33}$. **e**, Stability of $(NiCo)S_{1.33}$ under different applied voltages. **f**, The LSV of $(NiCo)S_{1.33}$ under the increasing temperature. The inset shows the calculated values of the activation energy.

The OER performance of the as-prepared nickel-cobalt bimetallic sulphides were evaluated under alkaline condition of 1.0 M KOH solution. The electrochemical impedance spectroscopy (EIS) (Supplementary Fig. 21d) indicated that the charge transfer resistance of $(NiCo)S_{1.33}$ during the reaction process was significantly reduced with the potential increasing, which could facilitate charge transfer. And the activation energy was calculated to be 12.97 $KJ\cdot mol^{-1}$ from the LSV under the increasing temperature (Supplementary Fig. 21f), which reflected the good catalytic performance of $(NiCo)S_{1.33}$. This good activity and stability of $(NiCo)S_{1.33}$ could be attributed to the specialization of the metal-sulfur coordination form induced by the the Ni_{Oh} and Co_{Oh}/Co_{Td} occuptions (Supplementary Fig. 21).

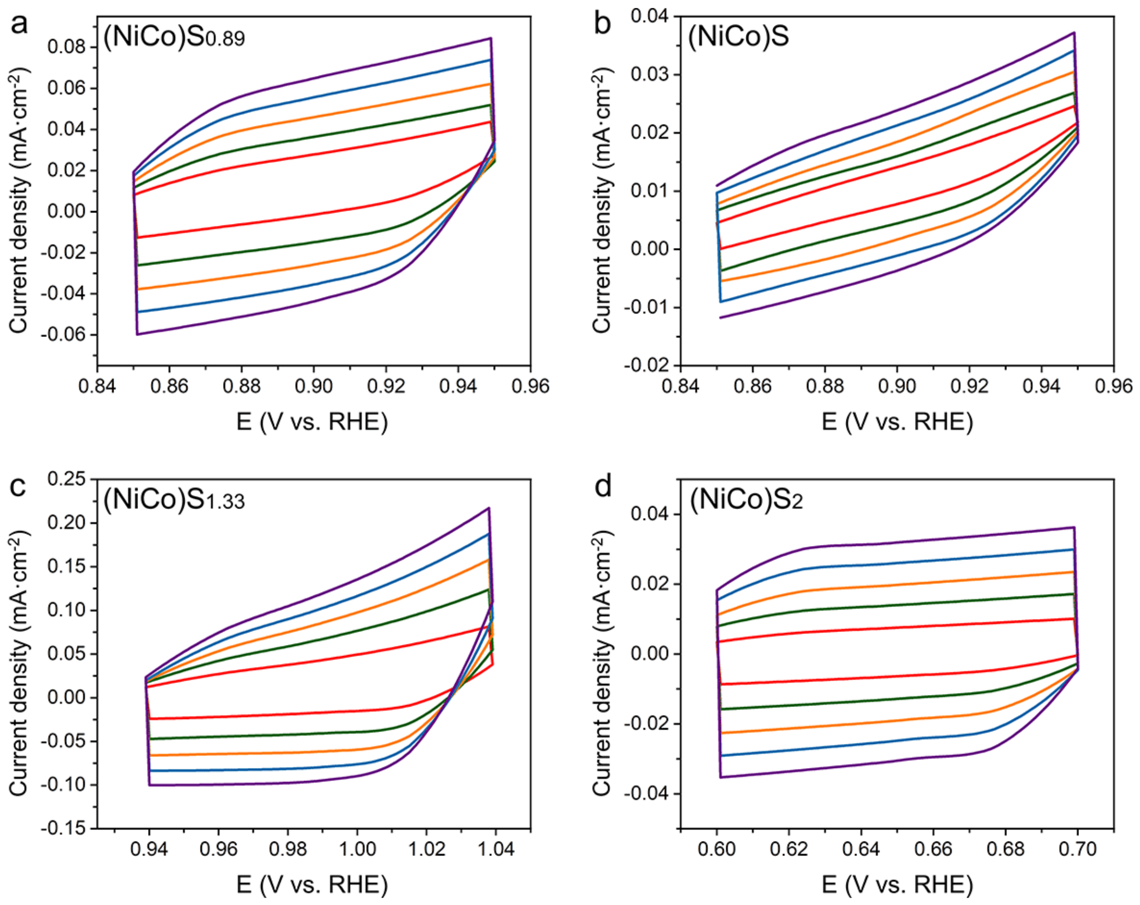

Supplementary Fig. 22 | Cyclic voltammetry of as-prepared nickel-cobalt bimetallic sulphides collected at various scanning rates during non-Faradaic process GC electrode. a, CV curves of $(NiCo)S_{0.89}$. **b**, CV curves of (NiCo)S. **c**, CV curves of $(NiCo)S_{1.33}$. **d**, CV curves of $(NiCo)S_2$.

The CV curves in Supplementary Fig. 22 are used to measure the electric double layer capacitance for further calculating the electrochemical active area of different sulphides (as shown in Supplementary Fig. 21 c). In the actual testing process, we first measured the open circuit potential of different sulphides, and then set the voltage range as OCP ± 0.05 V with the scan rate of 2, 4, 6, 8 and 10 mV/s, respectively. This potential range is in the non-Faraday current range, only the double layer charging and discharging process occurs, without any electrochemical reaction. And the CV curve will show a rectangular-like shape. Therefore, the edge of CV curves in Supplementary Fig. 22 are sharp, which is different from the smooth conversion of conventional CV curve (Supplementary Fig. 23) with complete electrochemical reaction.

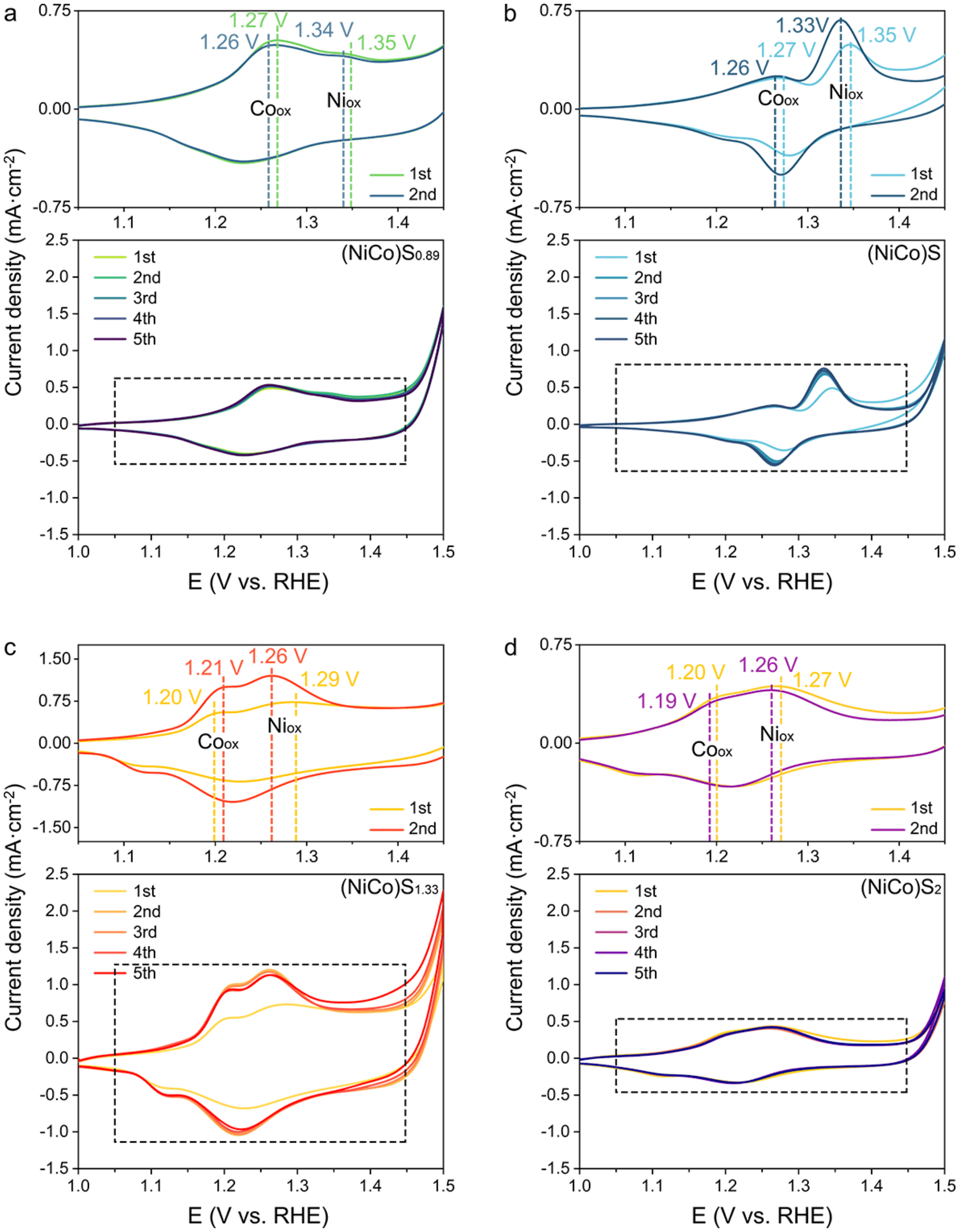

Supplementary Fig. 23 | CV curves of as-prepared nickel-cobalt bimetallic sulphides collected at a scan rate of 5 mV·s^{-1}. a, CV curves of $(NiCo)S_{0.89}$. **b**, CV curves of (NiCo)S. **c**, CV curves of $(NiCo)S_{1.33}$. **d**, CV curves of $(NiCo)S_2$.

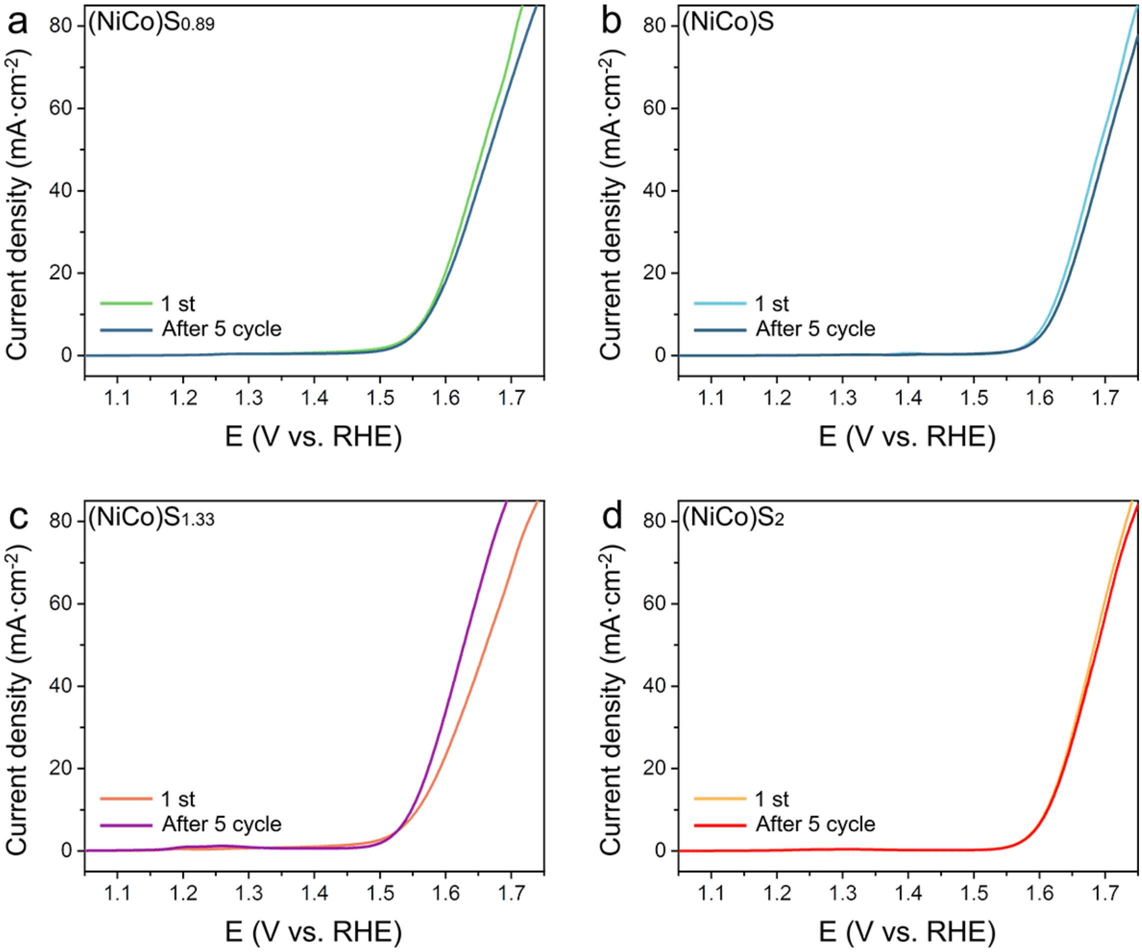

Supplementary Fig. 24 | iR-corrected LSV of as-prepared nickel-cobalt bimetallic sulphides collected before and after five CV cycles at a scan rate of 5 mV·s^{-1}. a, LSV of $(NiCo)S_{0.89}$. **b**, LSV of (NiCo)S. **c**, LSV of $(NiCo)S_{1.33}$. **d**, LSV of $(NiCo)S_2$.

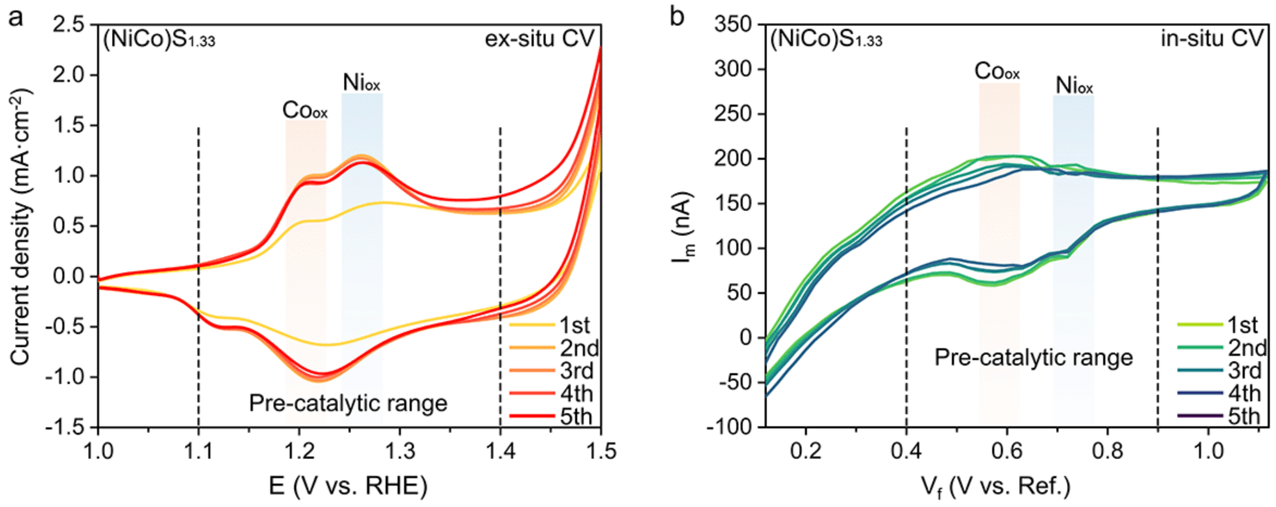

Supplementary Fig. 25 | CV curves of $(NiCo)S_{1.33}$ under the ex- and in-situ conditions. a, CV curves collected under the ex-situ conditions. **b**, CV curves collected under the in-situ conditions.

Firstly, the working electrode, counter electrode and reference electrode on the electrochemical chip used for in-situ experiment are all made of Pt. For ex-situ electrochemical measurement, the glassy carbon electrode, Pt-foil and Hg/HgO with 1.0 M KOH filling solution are used as the working, counter and reference electrodes, respectively. Secondly, in order to obtain a smooth and ideal curve, we have set the scan rate to 100 mV/s during the in-situ CV test. The parameter used in ex-situ electrochemical test is 10 mV/s (see **Electrical Measurements** and **in-situ Transmission Electron Microscope** in **Methods** for details). Thirdly, in order to obtain a clear field of vision and prevent a large number of samples from piling up and damaging the silicon nitride film of the chip during the in-situ experiment, we only dropped a small amount of pure sulphide nanoparticles on the chip, which results in a very few samples being loaded on the working electrode. However, the catalysts for ex-situ test were prepared by dispersing 3 mg of catalyst@C in 1450μL of N, N-Dimethylformamide (DMF) with 50μL of 5 wt% Nafion solution.

Therefore, we suggest that the three differences between the in-situ and ex-situ CV tests leads to the reversal of the relative strength of Ni and Co oxidation peaks. However, we believe that the intrinsic structure, elemental distribution and valence transition of catalysts should be consistent whether under in-situ or ex-situ conditions. The phenomena observed by in-situ experiments can reflect the actual electrochemical behaviours of sulphides in the OER process. Besides, the main purpose of in-situ CV curve is to determine the voltage range of the pre-catalytic stage and provide reference for setting applied potential for subsequent in-situ IT experiments.

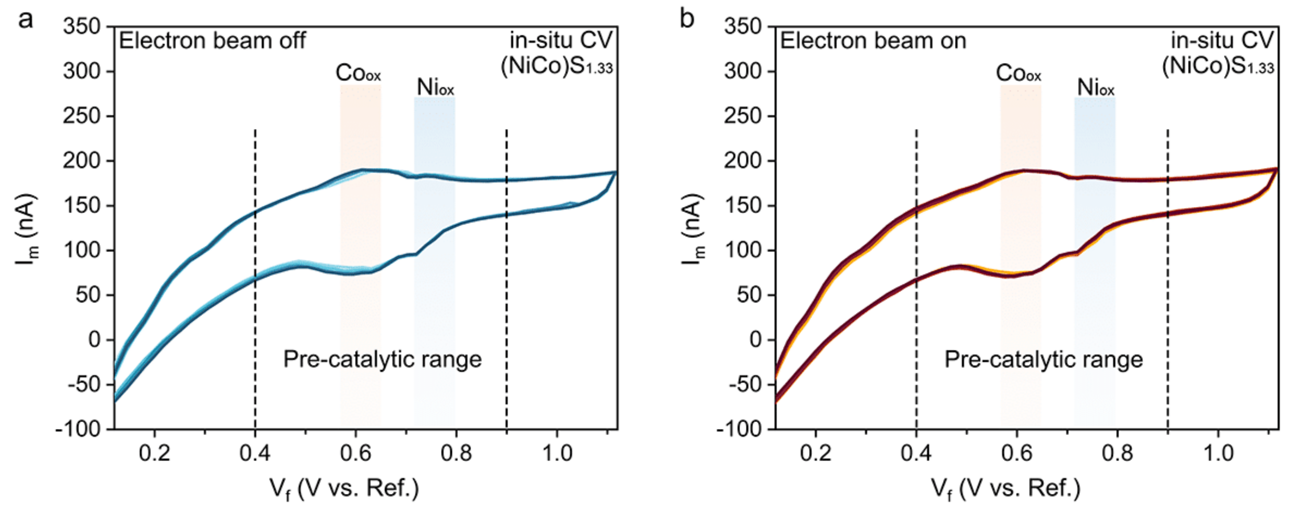

Supplementary Fig. 26 | in-situ CV curve of (NiCo)$S_{1.33}$ separately under the two conditions. a, in-situ CV curve collected with the electron beam isolation. **b**, in-situ CV curve collected with continuous electron beam irradiation.

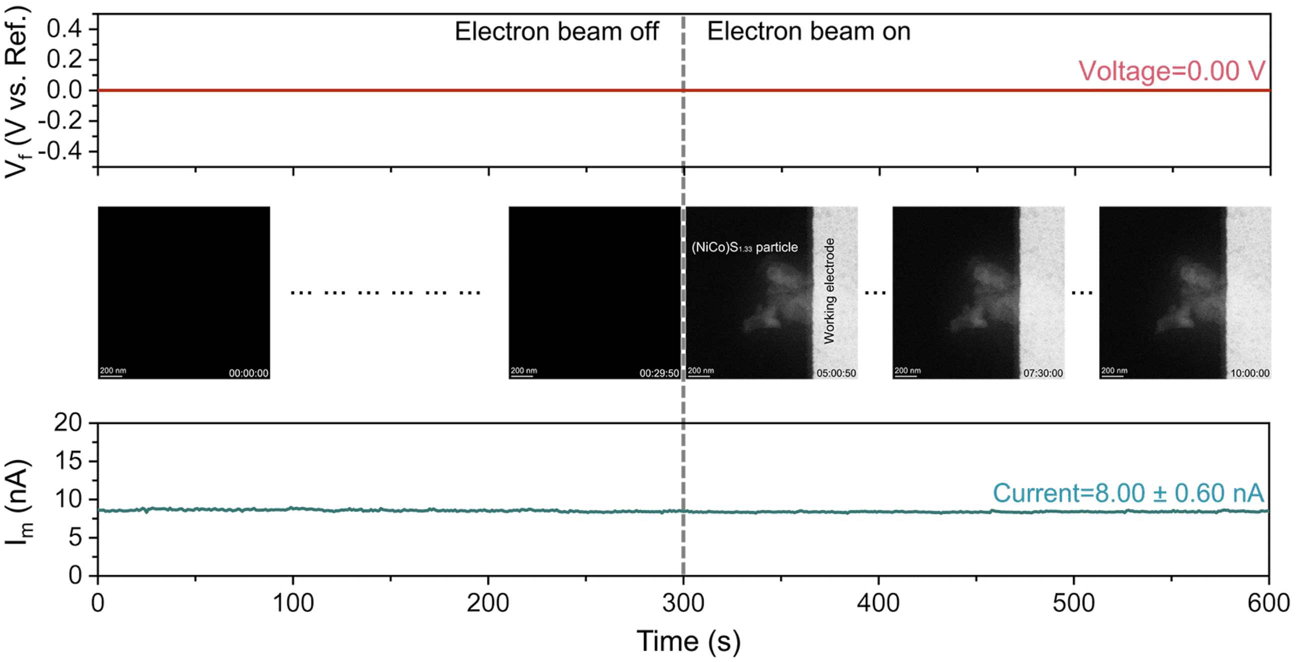

Supplementary Fig. 27 | Real-time record on the variation of current density with and without electron beam irradiation under the applied voltage of 0 V.

We have performed a control experiment to record the variation of current density with and without electron beam irradiation under the applied voltage of 0 V. As shown in Supplementary Fig. 27, it could be found that there is no significantly fluctuation of current density when the electron beam switched from the off state to on state. And the current density did not fluctuate significantly during the 5 minutes of electron beam irradiation. Besides, the Supplementary Fig. 26 has shown the in-situ CV curves of $(NiCo)S_{1.33}$ separately under the two conditions of electron beam isolation and continuous electron beam irradiation, which suggested that the effect of electron beam irradiation is negligible.

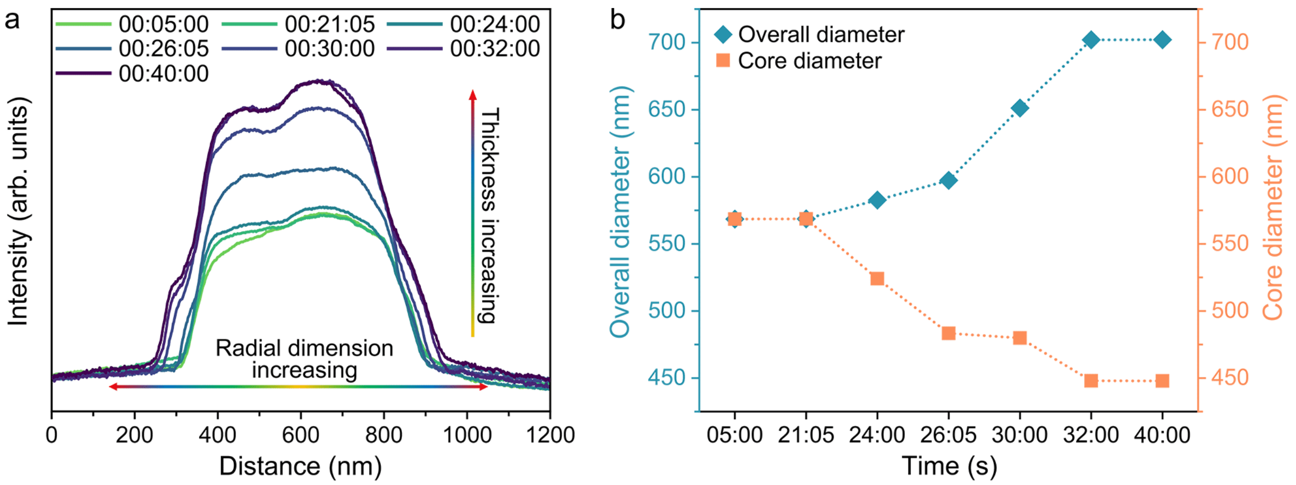

Supplementary Fig. 28 | Diameter evolution of (NiCo)$S_{1.33}$ nanoparticle during the reaction process. a, The radial dimension and thickness evolution curve of the central particle in Fig. 2e. **b,** The diameter evolution of overall and crystal core of the central particle in Fig. 2e.

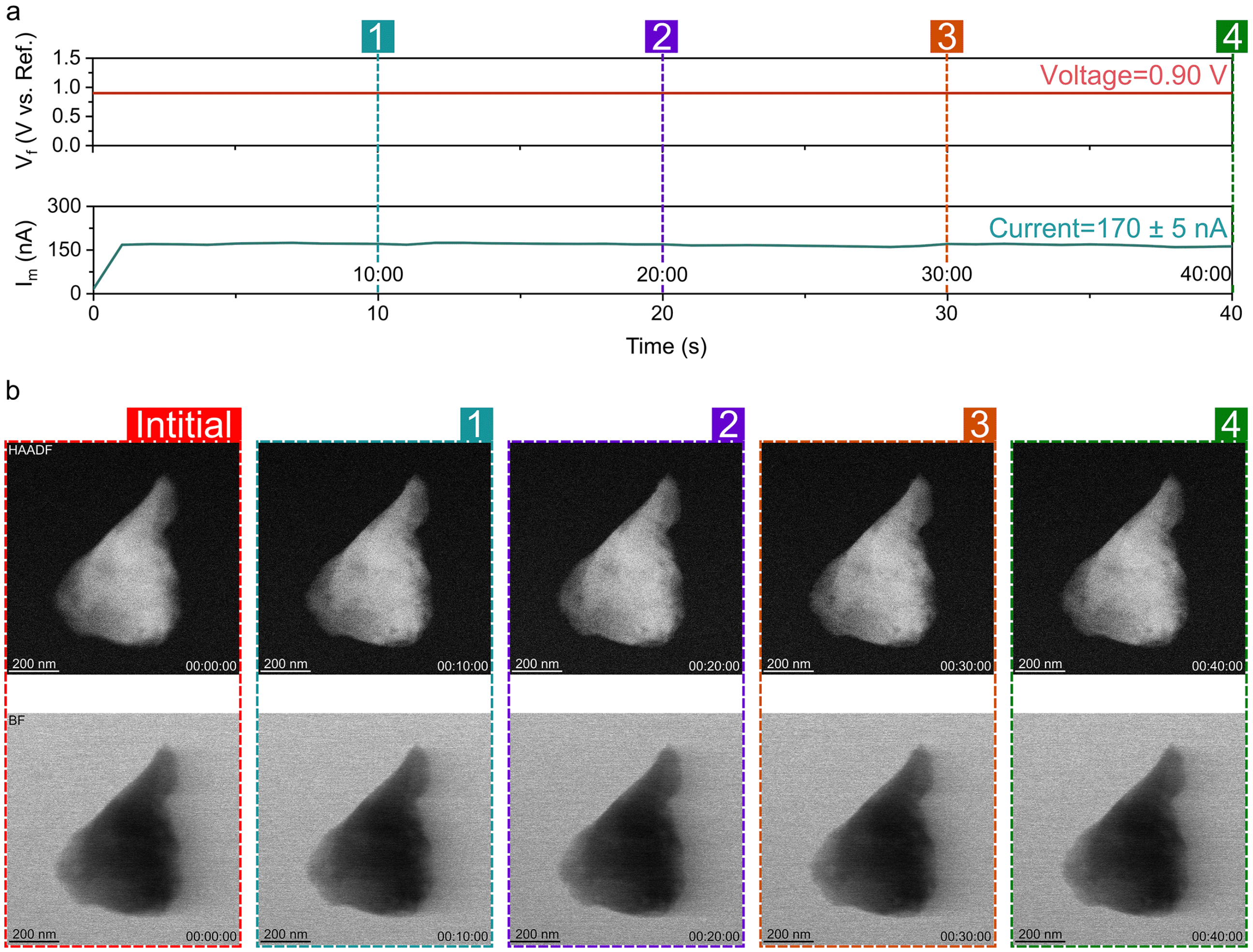

Supplementary Fig. 29 | Real-time observation on the variation of a (NiCo)$S_{1.33}$ particle away from the working electrode under the applied voltage of 0.9 V. a, The applied potential and corresponding current during the in-situ IT test. **b**, the HAADF and BF images of the (NiCo)$S_{1.33}$ particle at different times.

If it is Oswald ripening process that causes the increase of particle size and variation of morphology, the similar phenomenon should be observed under the same applied voltage conditions whether the particles are in contact with the working electrode or not. Therefore, we selected a nanoparticle, which has a comparable size with the central particle in Fig. 2e, without contacting the working electrode as the observation object and continuously monitor the morphology and size of this particle under the applied voltage of 0.9 V (Supplementary Fig. 29 a). According to Supplementary Fig. 29 b, it could be found that the morphology, size, and surface structure of this nanoparticle did not change during a period of up to 40 seconds.

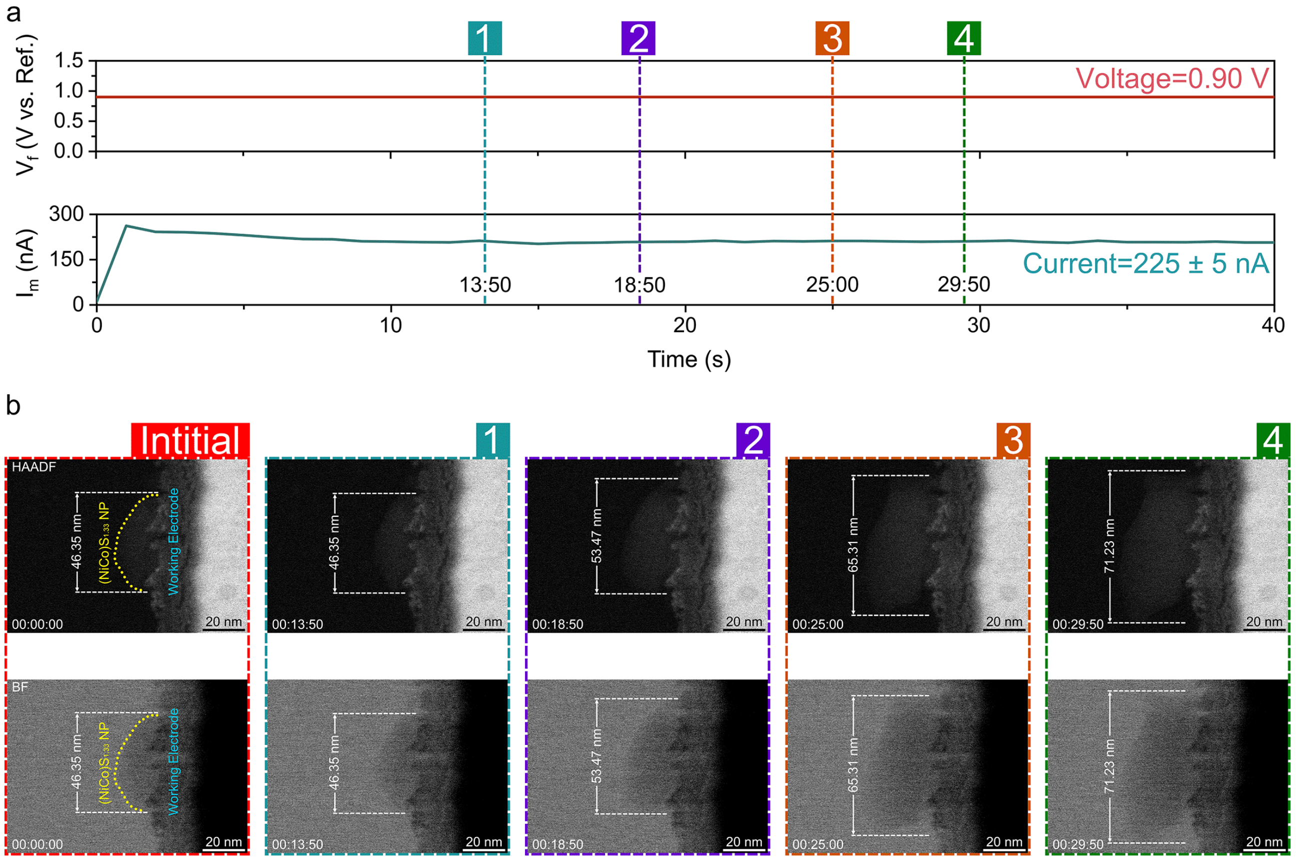

Supplementary Fig. 30 | Real-time observation on the variation of a small (NiCo)$S_{1.33}$ particle on the working electrode under the applied voltage of 0.9 V. a, The applied potential and corresponding current during the in-situ IT test. **b**, the HAADF and BF images of the small (NiCo)$S_{1.33}$ particle at different times.

According to the mechanism of Oswald ripening, the smaller particles will dissolve in the solvent, while the diameter of the large particles will continue to increase. Then, we could be able to observe the diameter reduction of the small particles during the dissolution process. Thus, we selected a small particle with a diameter of 46.35 nm as the observation object and continuously monitor the morphology and size of this small particle under the applied voltage of 0.9 V (Supplementary Fig. 30 a). As shown in Supplementary Fig. 30 b, the diameter of this small particle increased to 71.23 nm within 30 seconds, which suggested that there is no dissolution process occurred in the electrochemical environment.

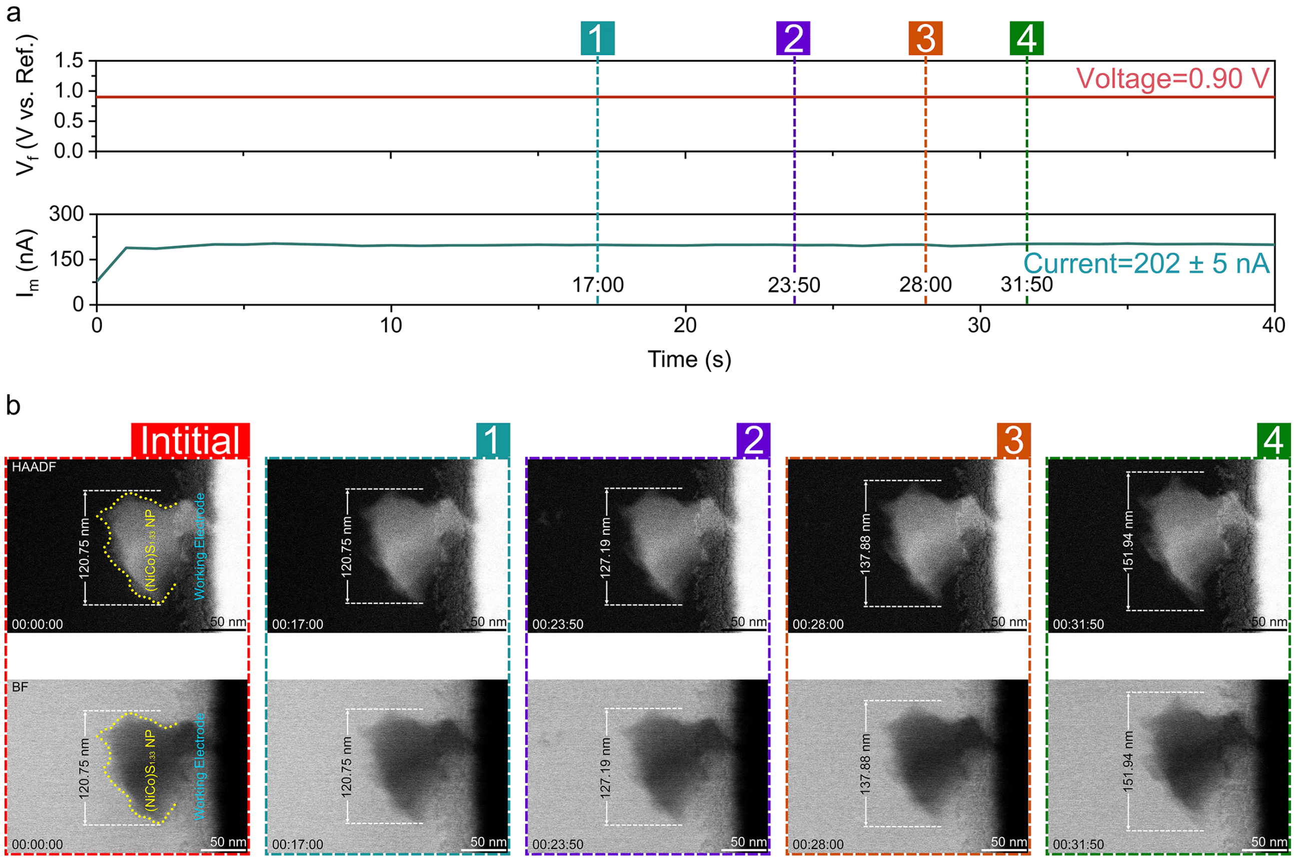

Supplementary Fig. 31 | Real-time observation on the variation of an individual (NiCo)$S_{1.33}$ particle on the working electrode under the applied voltage of 0.9 V. a, The applied potential and corresponding current during the in-situ IT test. **b**, the HAADF and BF images of the individual (NiCo)$S_{1.33}$ particle at different times.

We also selected an individual particle as the research object to observe the surface reconstruction process under the applied voltage of 0.9 V to provide a more convincing result. As shown in Supplementary Fig. 31, this individual particle exhibited the similar behaviour as those particles in Fig. 2d during the surface reconstruction process.

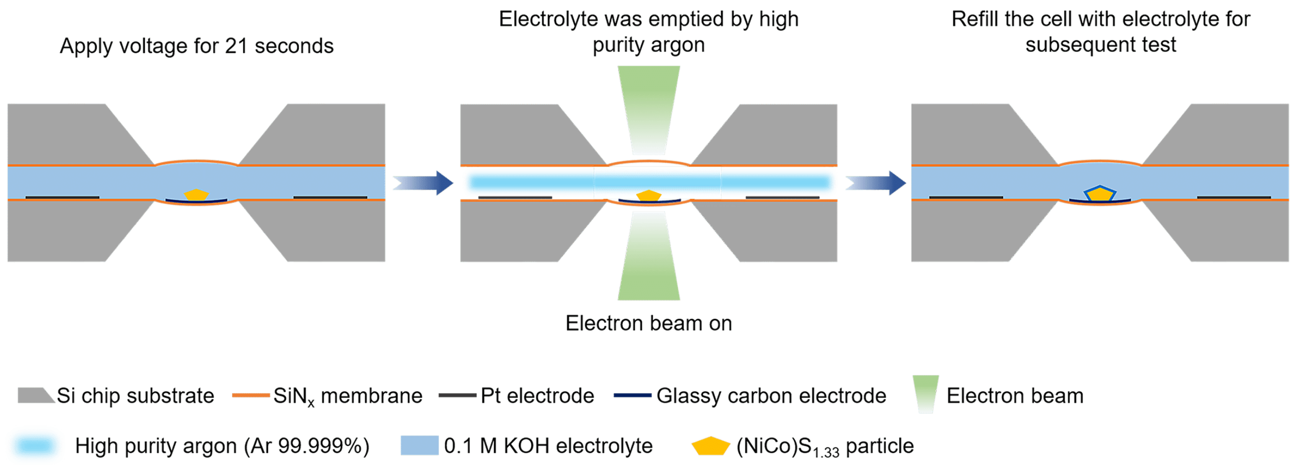

Supplementary Fig. 32 | Schematic diagram of test process and method for the in-situ EELS.

A specific voltage of 0, 0.9, 1.0, 1.1, 1.2 and 1.3 V (vs Pt pseudo-Reference) was applied for 21 seconds, respectively. After that, the electrolyte was emptied with the high purity argon, which can prevent the surface of catalyst particle from being oxidized, to improve the spatital resolution for structural and elemental analysis. The liquid cell was refilled with electrolyte for subsequent test with another voltage and repeat this process until all voltage conditions are finished.

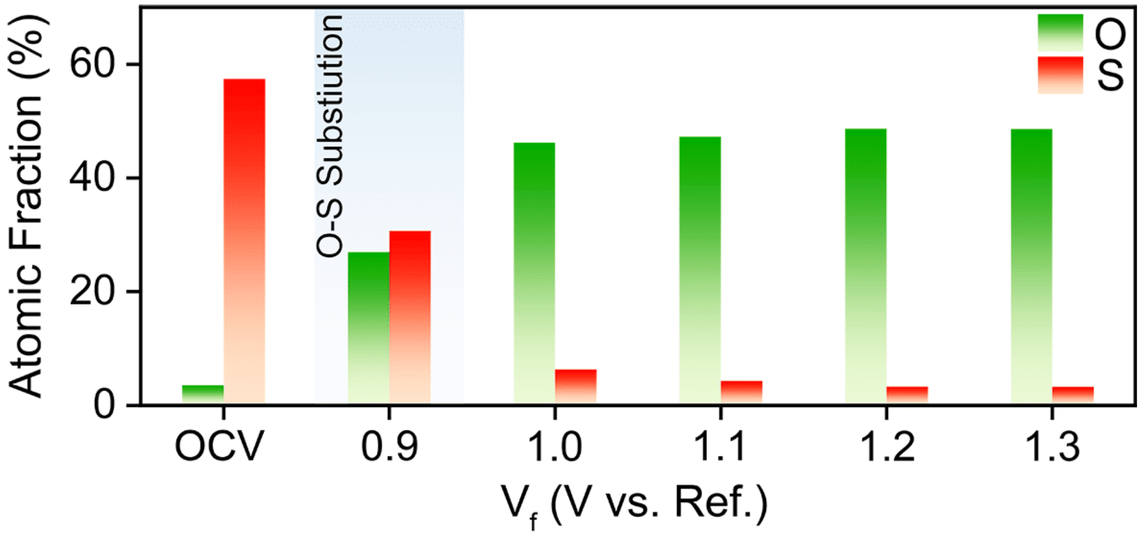

Supplementary Fig. 33 | Atomic fraction of S and O in $(NiCo)S_{1.33}$ under the different applied voltage.

in-situ structural and elemental evolution of $(NiCo)S_{0.89}$

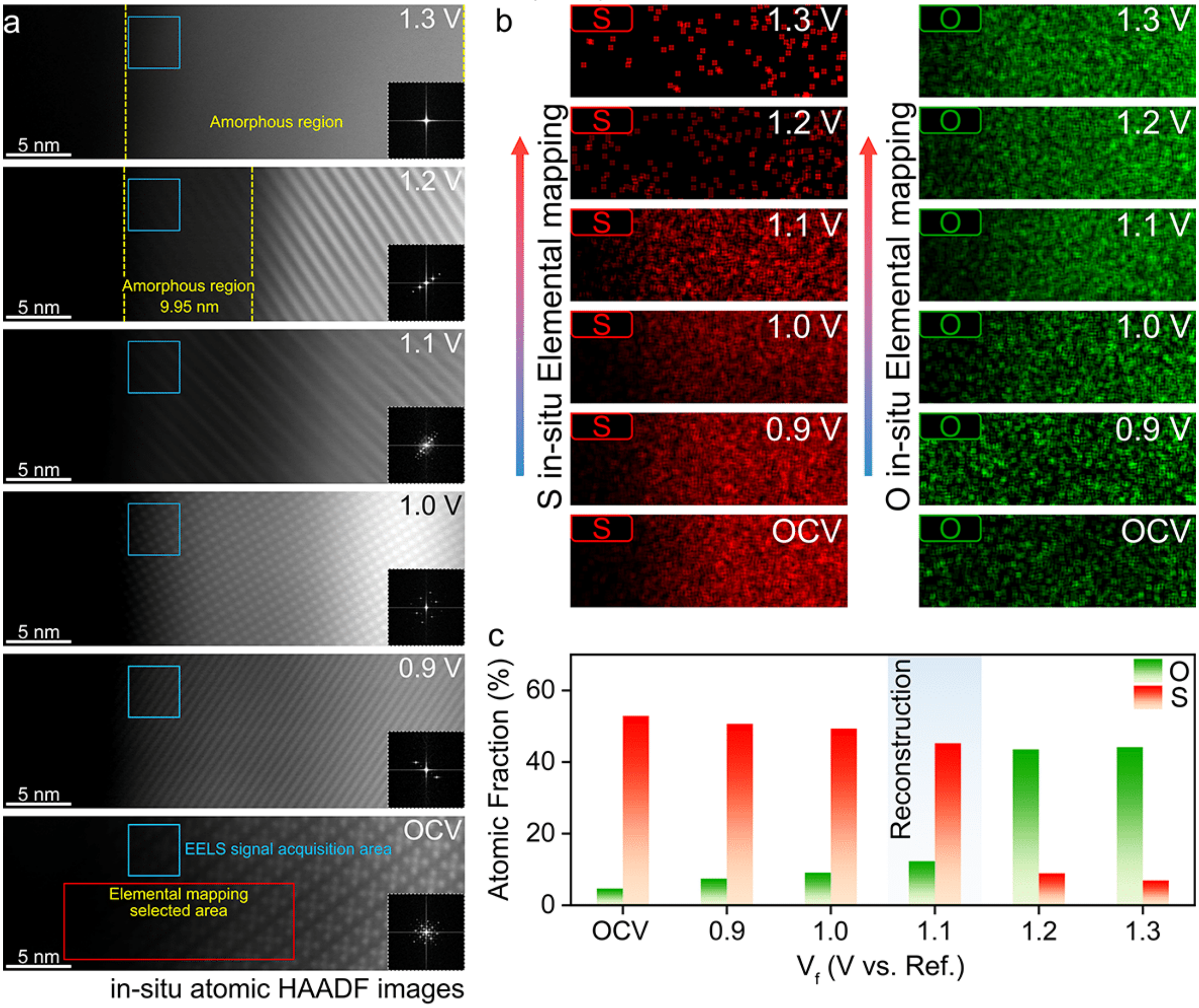

Supplementary Fig. 34 | Structural and elemental evolution of $(NiCo)S_{0.89}$ under different applied potentials. **a**, in-situ atomic HAADF images of $(NiCo)S_{0.89}$ after constantly applying different potentials for twenty seconds. **b**, in-situ elemental mapping of S and O under different applied potential. **c**, Atomic fraction of S and O in $(NiCo)S_{0.89}$ under the different applied voltage.

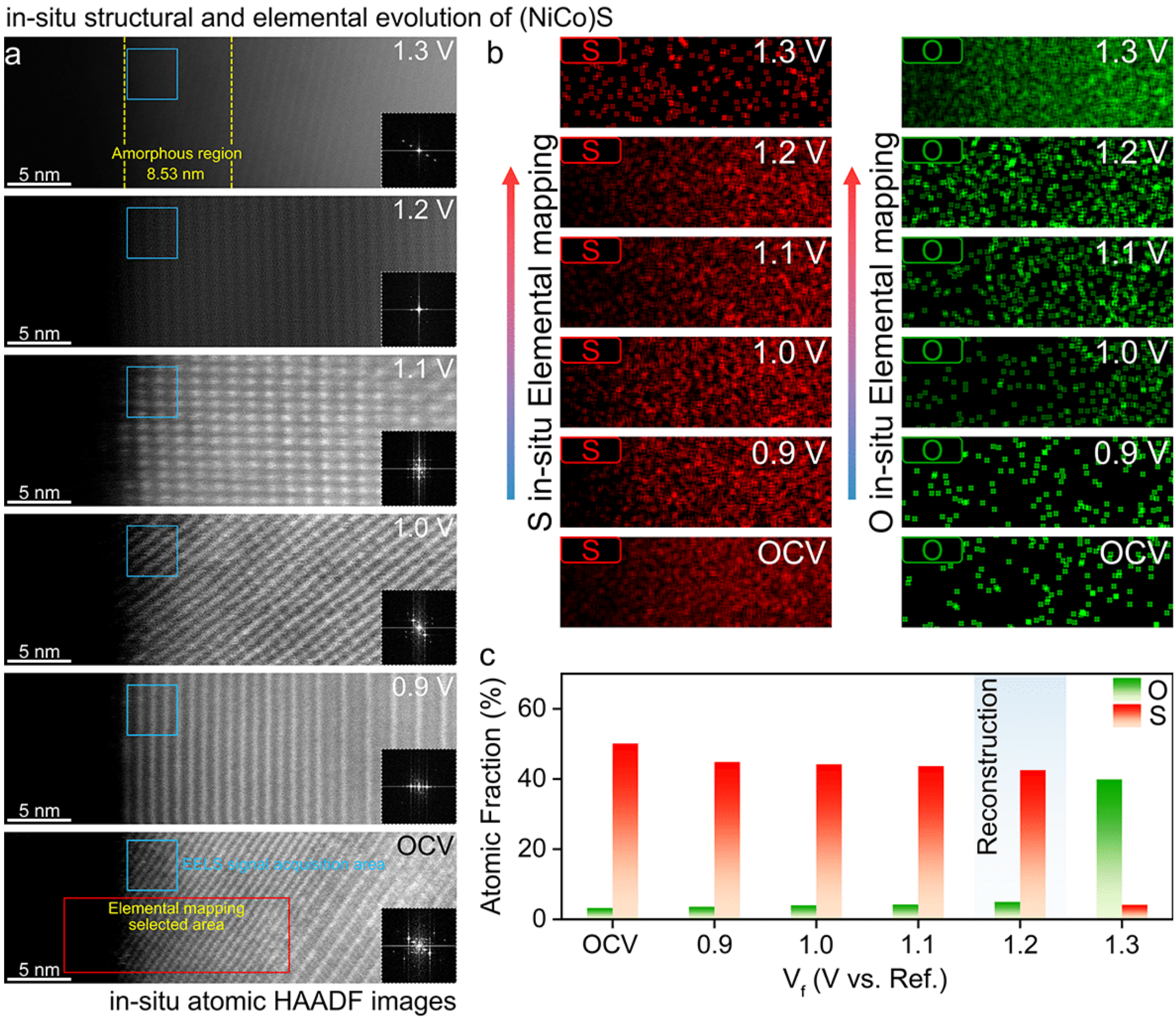

Supplementary Fig. 35 | Structural and elemental evolution of (NiCo)S under different applied potentials. a, in-situ atomic HAADF images of (NiCo)S after constantly applying different potentials for twenty seconds. **b**, in-situ elemental mapping of S and O under different applied potential. **c**, Atomic fraction of S and O in (NiCo)S under the different applied voltage.

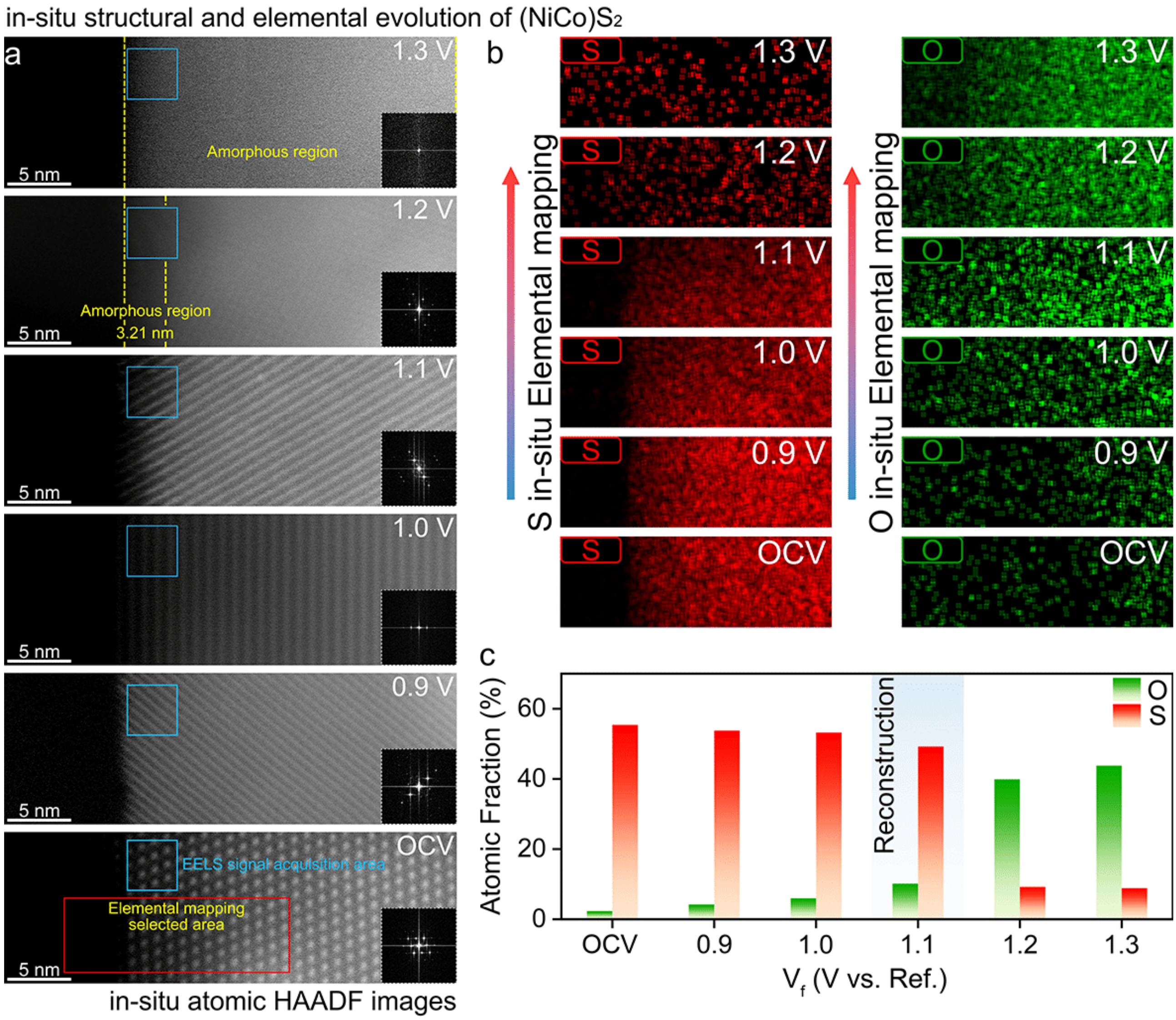

Supplementary Fig. 36 | Structural and elemental evolution of $(NiCo)S_2$ under different applied potentials. a, in-situ atomic HAADF images of $(NiCo)S_2$ after constantly applying different potentials for twenty seconds. **b**, in-situ elemental mapping of S and O under different applied potential. **c**, Atomic fraction of S and O in $(NiCo)S_2$ under the different applied voltage.

If the electron beam irradiation will cause the leaching of S, the prolonged irradiation will inevitably lead to the reduction of sulphur content in the sample. Thus, we have designed an irradiation experiment to investigate whether electron beam irradiation leads to the leaching of sulphur.

First of all, the screen current is an important parameter to measure the irradiation intensity of electron beam. The higher screen current, the more electrons will bombard the sample per unit time. In order to minimize the influence of electron beam irradiation on the sample, the screen current was set to 30 pA during the in-situ electrochemical-TEM experiment.

Based on the parameter setting of screen current in the in-situ EC-TEM experiment, the $(NiCo)S_{1.33}$ particles were continuously irradiated for 10 minutes at the screen current of 30 pA and 300 pA respectively in the irradiation experiment. And the elemental distribution and relative content of S before and after irradiation were characterized and measured. In addition, considering the difference between vacuum and liquid environments, we have conducted the irradiation experiments in vacuum and liquid (0.1 M KOH) environment respectively to obtain more comprehensive comparison results.

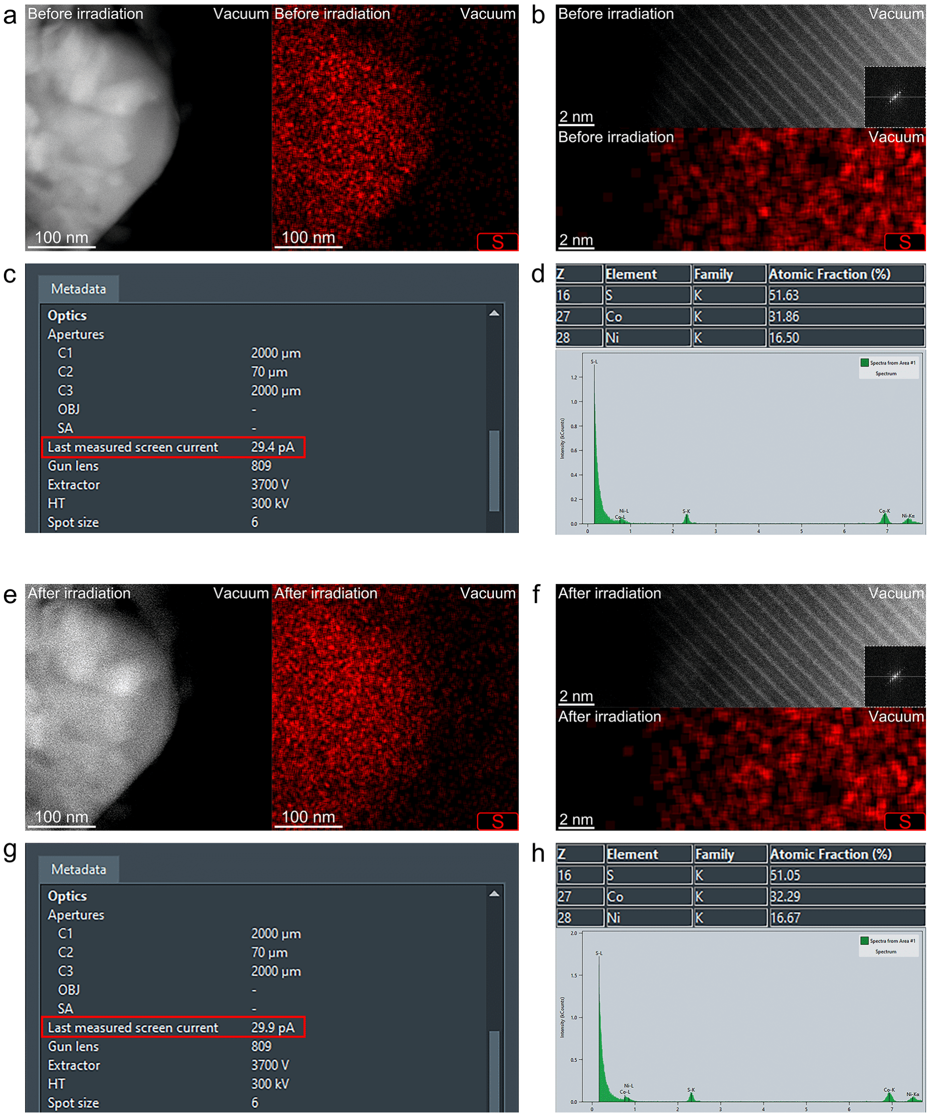

Supplementary Fig. 37 | Electron beam irradiation experiment of (NiCo)$S_{1.33}$ particles at the screen current of 30 pA in the vacuum environment. a, Low magnification HAADF image and elemental mapping of S before irradiation. **b**, High magnification HAADF image and elemental mapping of S before irradiation. **c**, Screenshot of the screen current parameter before irradiation. **d**, Relative contents of different elements and corresponding EDX element spectra before irradiation. **e**, Low magnification HAADF image and elemental mapping of S after irradiation. **f**, High magnification HAADF image and elemental mapping of S after irradiation. **g**, Screenshot of the screen current parameter after irradiation. **h**, Relative contents of different elements and corresponding EDX element spectra after irradiation.

As shown in Supplementary Fig. 37, the relative content and distribution of S did not change significantly after 10 minutes irradiation at the screen current of 30 pA. This result revealed that the electron beam irradiation with the screen current of 30 pA did not lead to the leaching of sulphur in the vacuum environment.

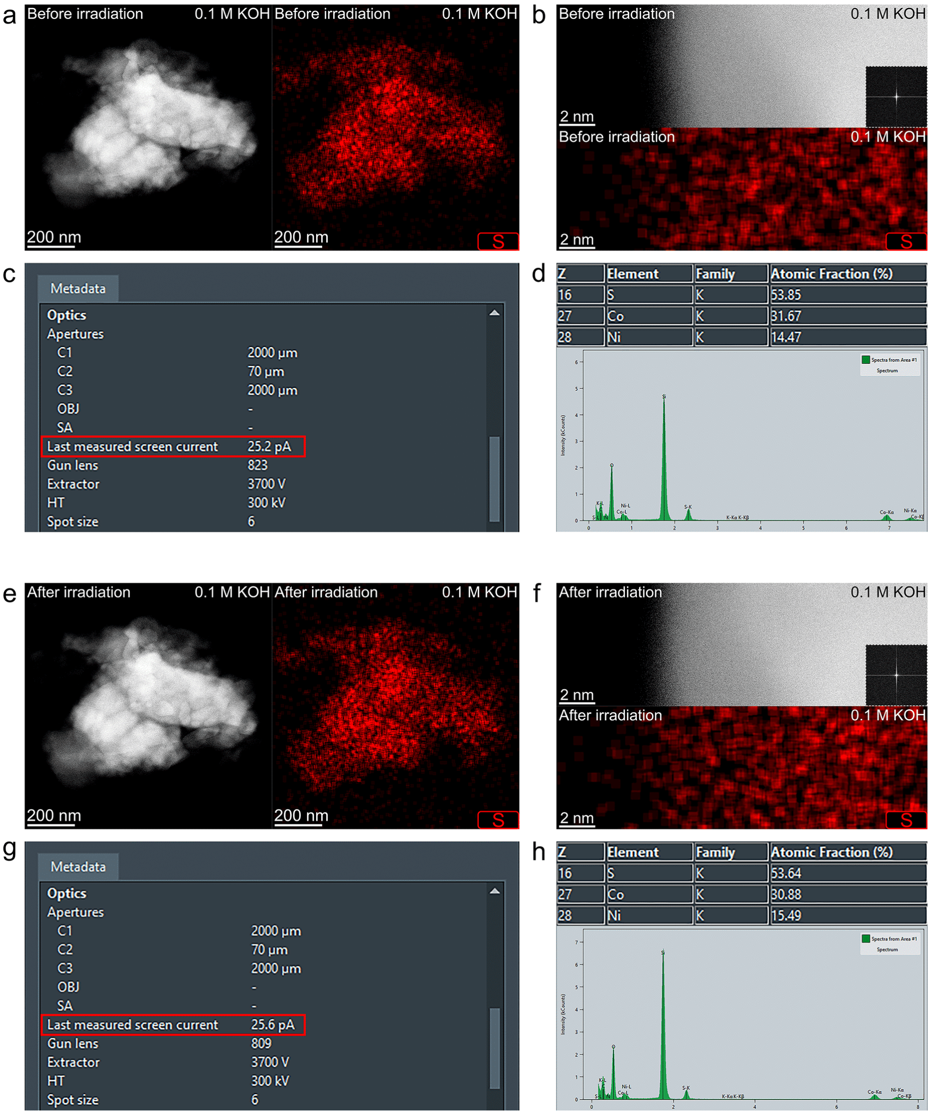

Supplementary Fig. 38 | Electron beam irradiation experiment of (NiCo)$S_{1.33}$ particles at the screen current of 30 pA in the liquid environment. a, Low magnification HAADF image and elemental mapping of S before irradiation. **b**, High magnification HAADF image and elemental mapping of S before irradiation. **c**, Screenshot of the screen current parameter before irradiation. **d**, Relative contents of different elements and corresponding EDX element spectra before irradiation. **e**, Low magnification HAADF image and elemental mapping of S after irradiation. **f**, High magnification HAADF image and elemental mapping of S after irradiation. **g**, Screenshot of the screen current parameter after irradiation. **h**, Relative contents of different elements and corresponding EDX element spectra after irradiation.

As shown in Supplementary Fig. 38, the relative content and distribution of S did not change significantly after 10 minutes irradiation at the screen current of 30 pA. This result revealed that the electron beam irradiation with the screen current of 30 pA did not lead to the leaching of sulphur in the liquid environment. (The screen current reduced to 25 pA due to the scattering effect of liquid on electron beam. The extra Si signal in the EDX spectrum comes from the silicon nitride film of the electrochemical chip.)

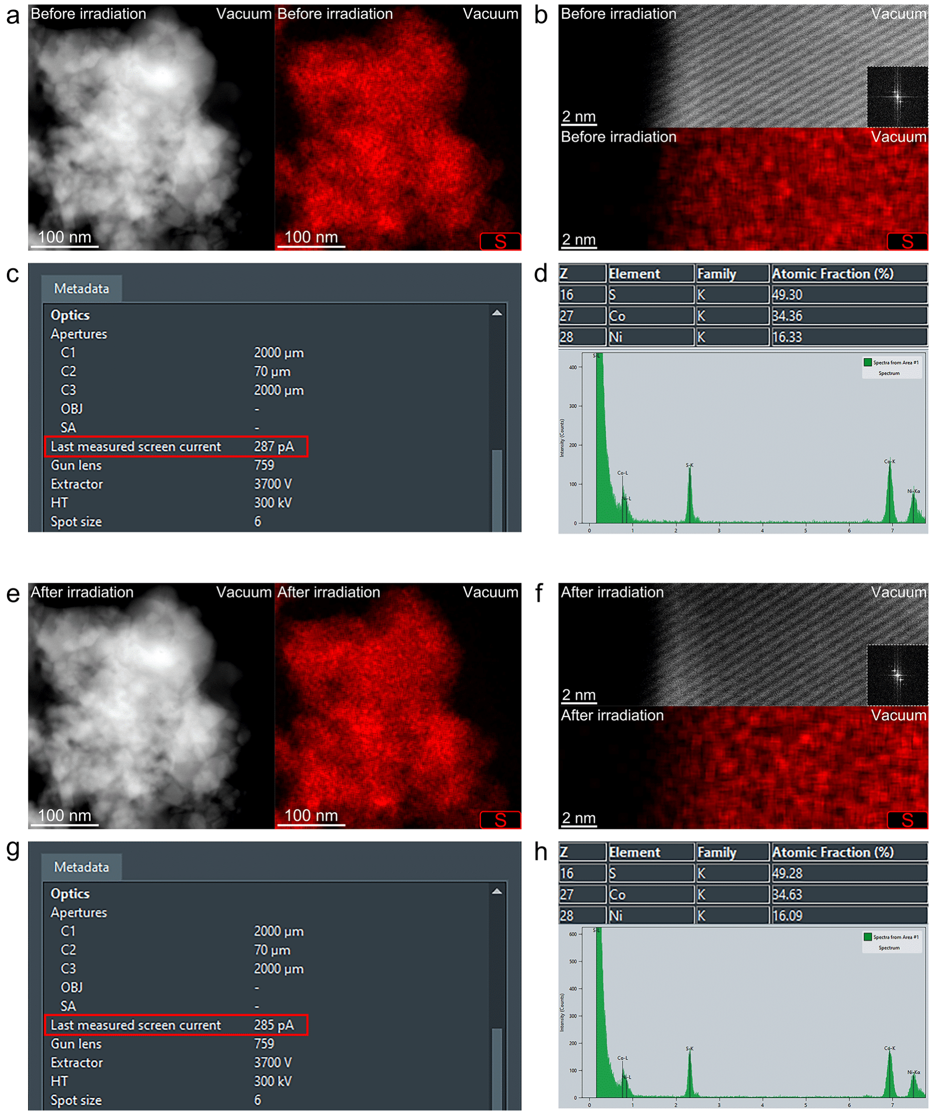

Supplementary Fig. 39 | Electron beam irradiation experiment of (NiCo)$S_{1.33}$ particles at the screen current of 300 pA in the vacuum environment. a, Low magnification HAADF image and elemental mapping of S before irradiation. **b**, High magnification HAADF image and elemental mapping of S before irradiation. **c**, Screenshot of the screen current parameter before irradiation. **d**, Relative contents of different elements and corresponding EDX element spectra before irradiation. **e**, Low magnification HAADF image and elemental mapping of S after irradiation. **f**, High magnification HAADF image and elemental mapping of S after irradiation. **g**, Screenshot of the screen current parameter after irradiation. **h**, Relative contents of different elements and corresponding EDX element spectra after irradiation.

As shown in Supplementary Fig. 39, the relative content and distribution of S did not change significantly after 10 minutes irradiation at the screen current of 300 pA. This result revealed that the electron beam irradiation with the screen current of 300 pA did not lead to the leaching of sulphur in the vacuum environment.

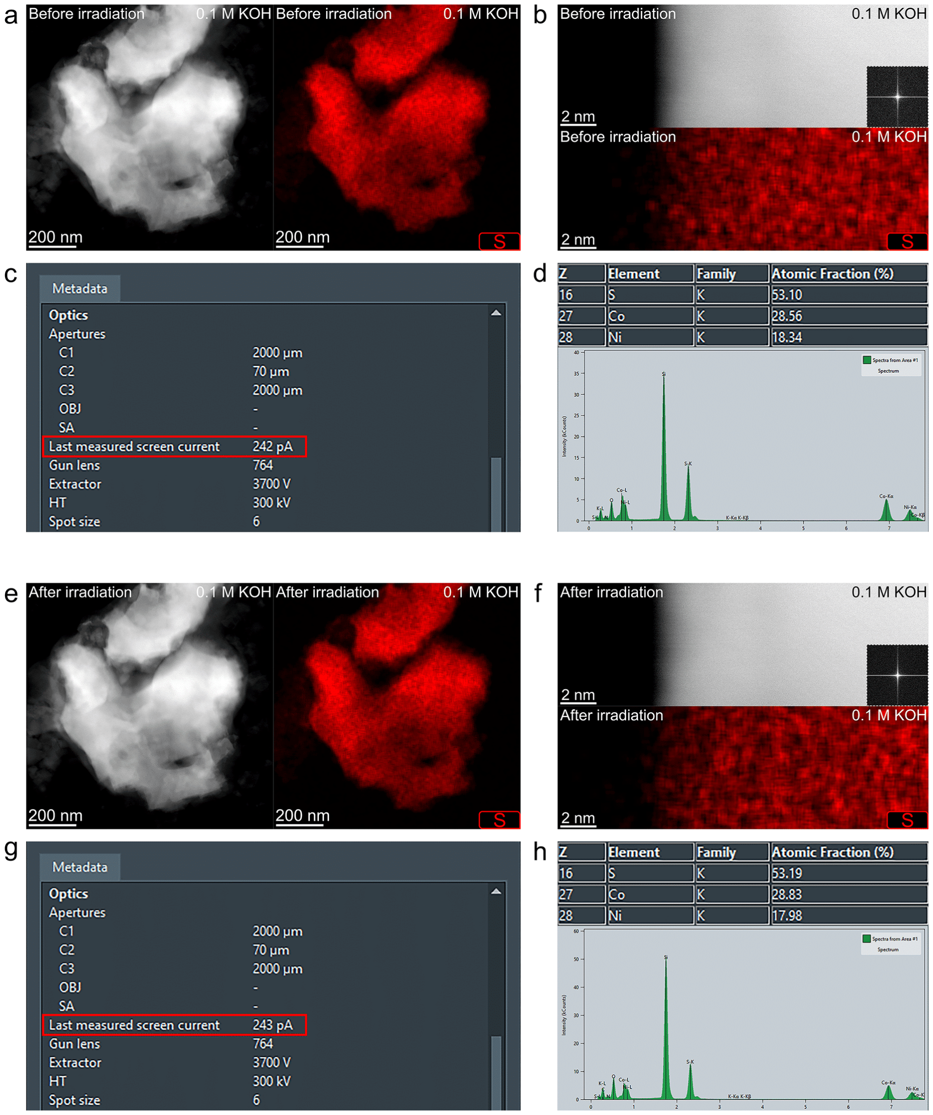

Supplementary Fig. 40 | Electron beam irradiation experiment of (NiCo)$S_{1.33}$ particles at the screen current of 300 pA in the liquid environment. a, Low magnification HAADF image and elemental mapping of S before irradiation. **b**, High magnification HAADF image and elemental mapping of S before irradiation. **c**, Screenshot of the screen current parameter before irradiation. **d**, Relative contents of different elements and corresponding EDX element spectra before irradiation. **e**, Low magnification HAADF image and elemental mapping of S after irradiation. **f**, High magnification HAADF image and elemental mapping of S after irradiation. **g**, Screenshot of the screen current parameter after irradiation. **h**, Relative contents of different elements and corresponding EDX element spectra after irradiation.

As shown in Supplementary Fig. 40, the relative content and distribution of S did not change significantly after 10 minutes irradiation at the screen current of 300 pA. This result revealed that the electron beam irradiation with the screen current of 300 pA did not lead to the leaching of sulphur in the liquid environment.

The above experimental results have revealed that the continuous electron beam irradiation is not the cause of sulphur leaching in our in-situ EC TEM at the current of 30 pA.

In order to investigate whether the high temperature will lead to the leaching of sulphur in $(NiCo)S_{1.33}$, we have designed an in-situ heating experiment to monitor the variation of relative content and distribution of S under the temperature of 500 ° C.

As shown in Supplementary Fig. 41, the temperature raised up from room temperature (25 ℃) to 500 ℃ within 950 seconds and held at 500 ℃ for 10 minutes. The relative content and distribution of S was obtained at 25 ℃ and 500 ℃ respectively to monitor the changes before and after heating. Besides, this heating experiment was conducted in a vacuum environment.

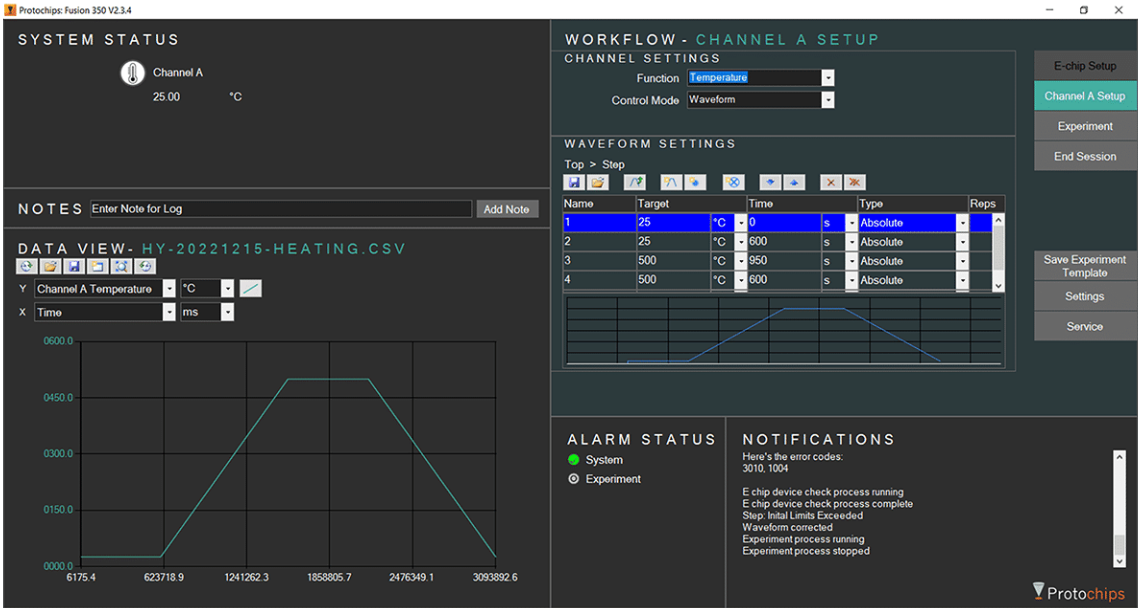

Supplementary Fig. 41 | Screenshot of the temperature and time parameters of in-situ heating experiment from the control software.

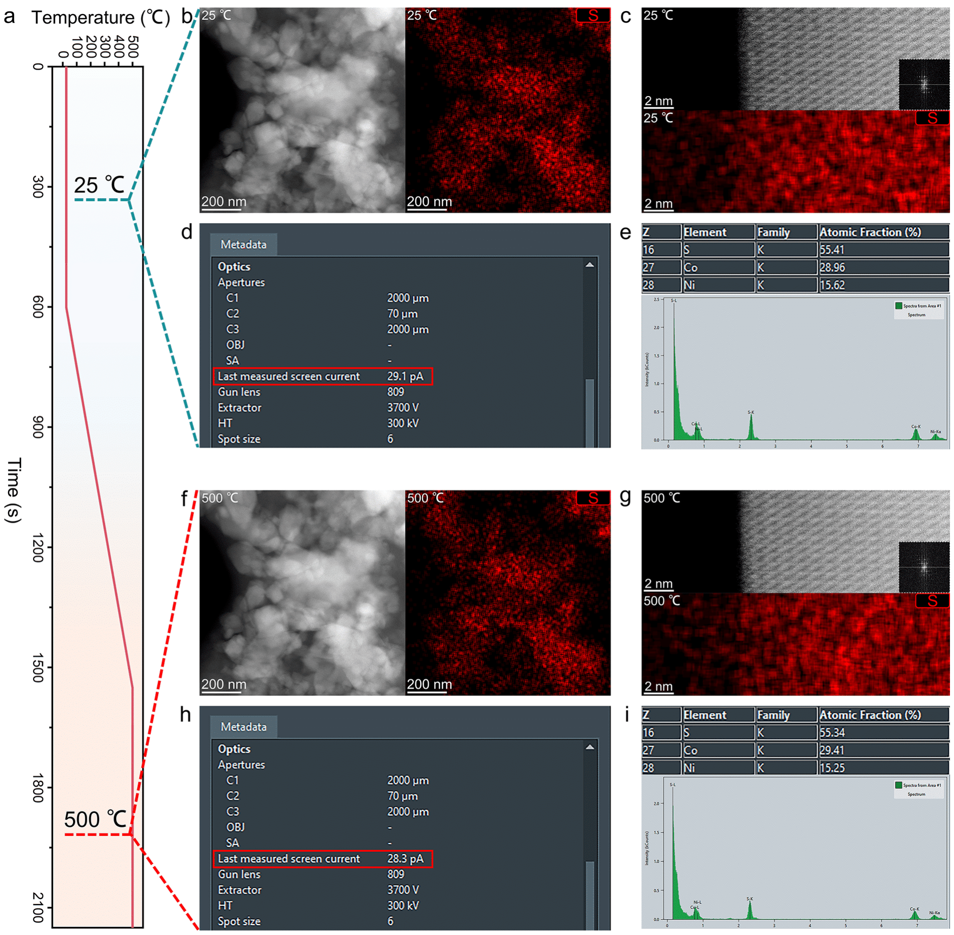

Supplementary Fig. 42 | in-situ heating experiment of $(NiCo)S_{1.33}$ particles at the screen current of 30 pA in the vacuum environment. a, the temperature-time curve. **b**, Low magnification HAADF image and elemental mapping of S at 25 °C. **c**, High magnification HAADF image and elemental mapping of S at 25 °C. **d**, Screenshot of the screen current parameter at 25 °C. **e**, Relative contents of different elements and corresponding EDX element spectra at 25 °C. **f**, Low magnification HAADF image and elemental mapping of S at 500 °C. **g**, High magnification HAADF image and elemental mapping of S at 500 °C. **h**, Screenshot of the screen current parameter at 500 °C. **i**, Relative contents of different elements and corresponding EDX element spectra at 500 °C.

As shown in Supplementary Fig. 42, the relative content and distribution of S did not change significantly after heating up to 500 °C at the screen current of 30 pA. This result revealed that the high temperature is not the cause of sulfur leaching in our in-situ EC-TEM at the current of 30 pA. In addition, the electrolyte with room temperature was constantly flowing during the in-situ EC TEM experiment, which would counteract the heating effect caused by electron beam irradiation at a certain extent and keep the catalyst particles in a constant temperature state.

In order to investigate whether the high temperature will lead to the leaching of sulphur in (NiCo)$S_{1.33}$ through another analytical technique and then correlate with the electron microscopy studies. We have performed the thermogravimetric analysis to carry out a macroscopical result to make up for the deficiency of microscopic electron microscope analysis.

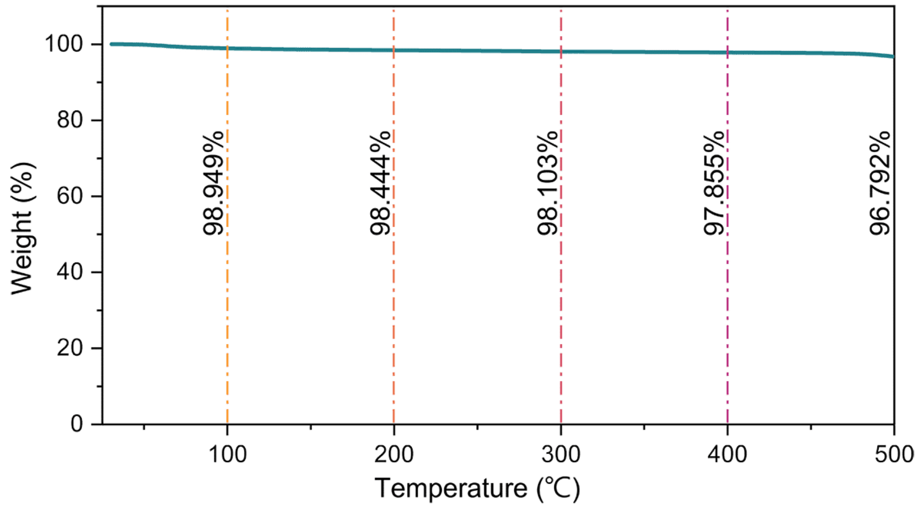

Supplementary Fig. 43 | Thermogravimetric analysis of (NiCo)$S_{1.33}$ powder during the process of heating up to 500 ℃ in the N_2 environment.

As shown in Supplementary Fig. 43, the overall quality of (NiCo)$S_{1.33}$ powder did not change significantly during the process of heating up to 500 ℃ in the nitrogen environment.

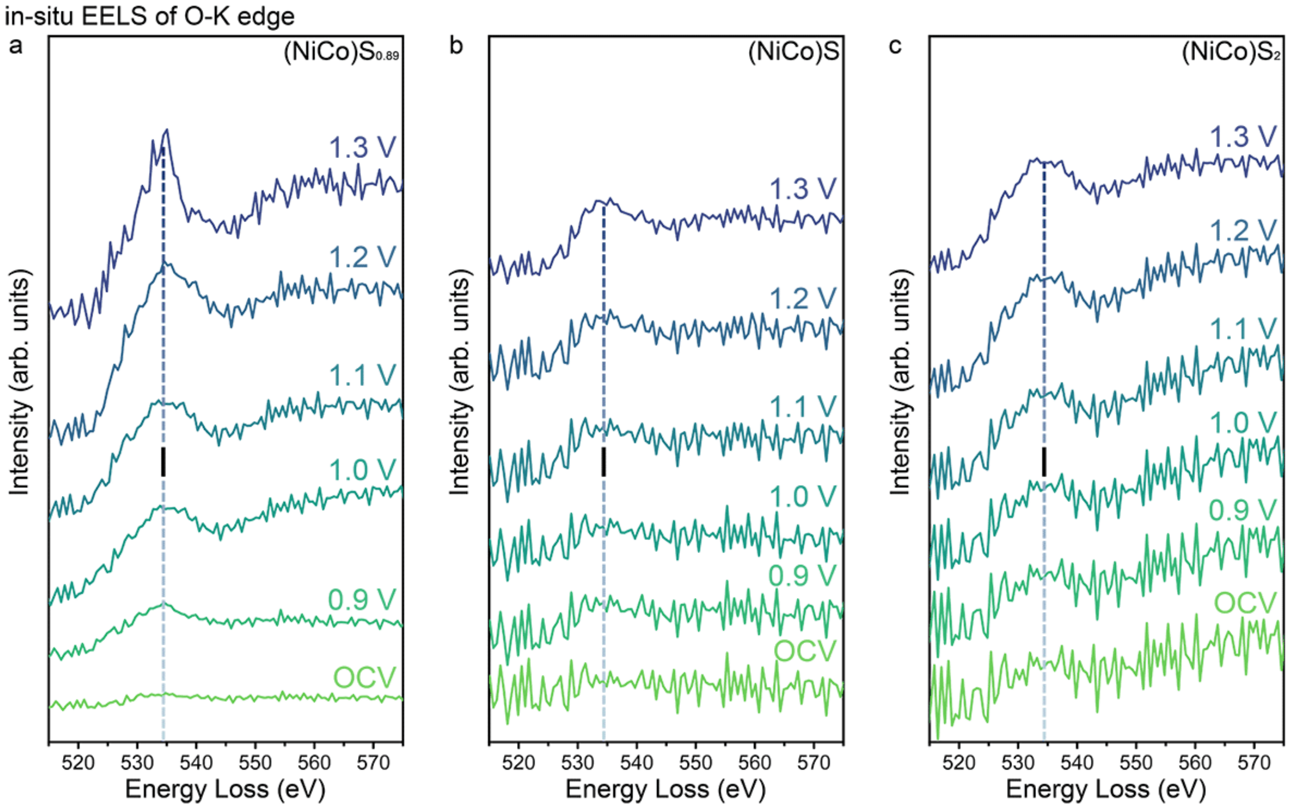

Supplementary Fig. 44 | in-situ EELS spectrum of O K-edge under different applied potential. a-c, O K-edge spectra collected under different applied potential of $(NiCo)S_{0.89}$, (NiCo)S and $(NiCo)S_2$, respectively. The I peaks can be assigned to the characteristic peaks of oxygen in amorphous structure.

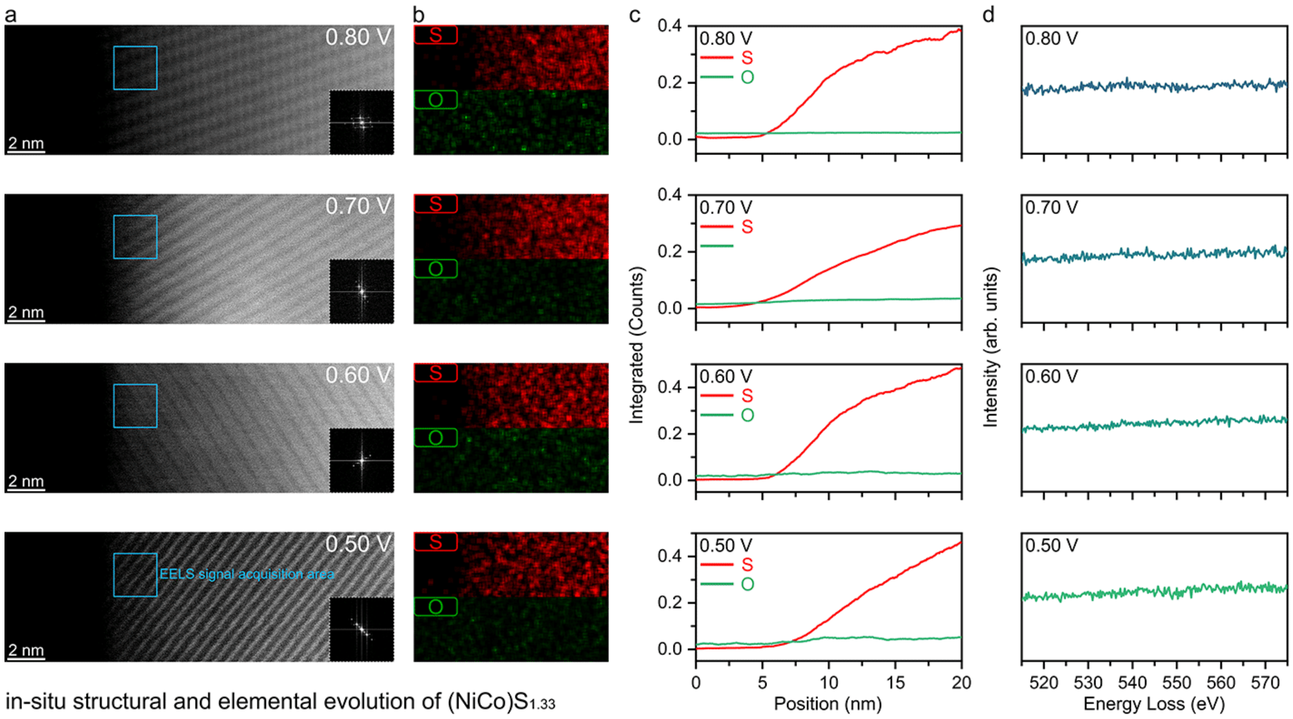

Supplementary Fig. 45 | Structural, elemental evolution and O K-edge of (NiCo)$S_{1.33}$ under different applied potentials. **a**, in-situ atomic HAADF images of (NiCo)$S_{1.33}$ after constantly applying different potentials for twenty seconds. **b**, in-situ elemental mapping of S and O under different applied potential. **c**, Corresponding elemental distribution curve extracted from **b**. **d**, in-situ EELS spectrum of O K-edge under different applied potential.

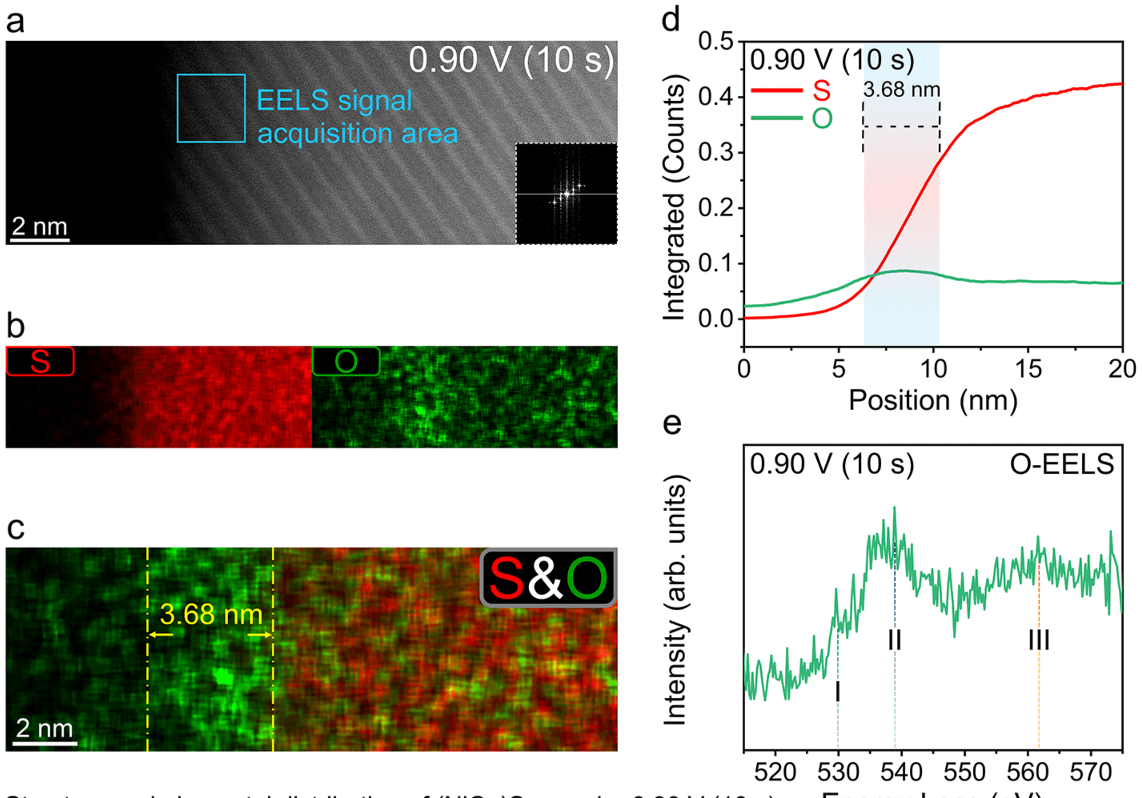

Structure and elemental distribution of $(NiCo)S_{1.33}$ under 0.90 V (10 s)

Supplementary Fig. 46 | Structural, elemental evolution and O K-edge of $(NiCo)S_{1.33}$ under the applied potential of 0.9 V after 10 s. a, Atomic HAADF image. **b**, Separated elemental mapping of S and O. **c**, Mixed elemantal mapping of S and O. **d**, The corresponding elemental distribution curve extracted from **c**. **e**, EELS spectrum of O K-edge. The I, II and III peaks can be assigned to the hybridization of O 2p with Co 3d, Ni 3d and Co 4sp orbitals, respectively.

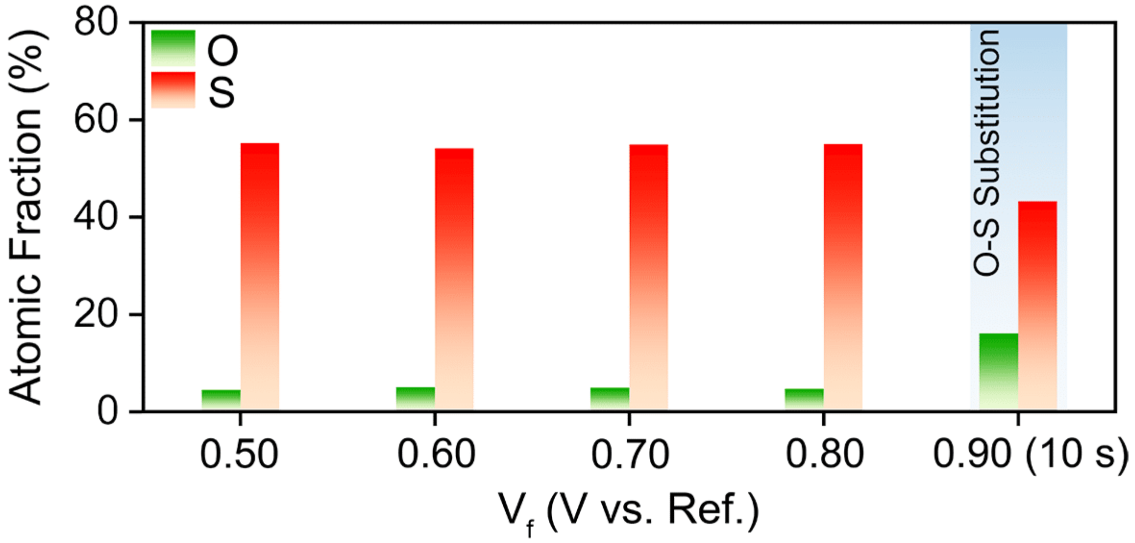

Supplementary Fig. 47 | Atomic fraction of S and O in $(NiCo)S_{1.33}$ under the different applied voltage.

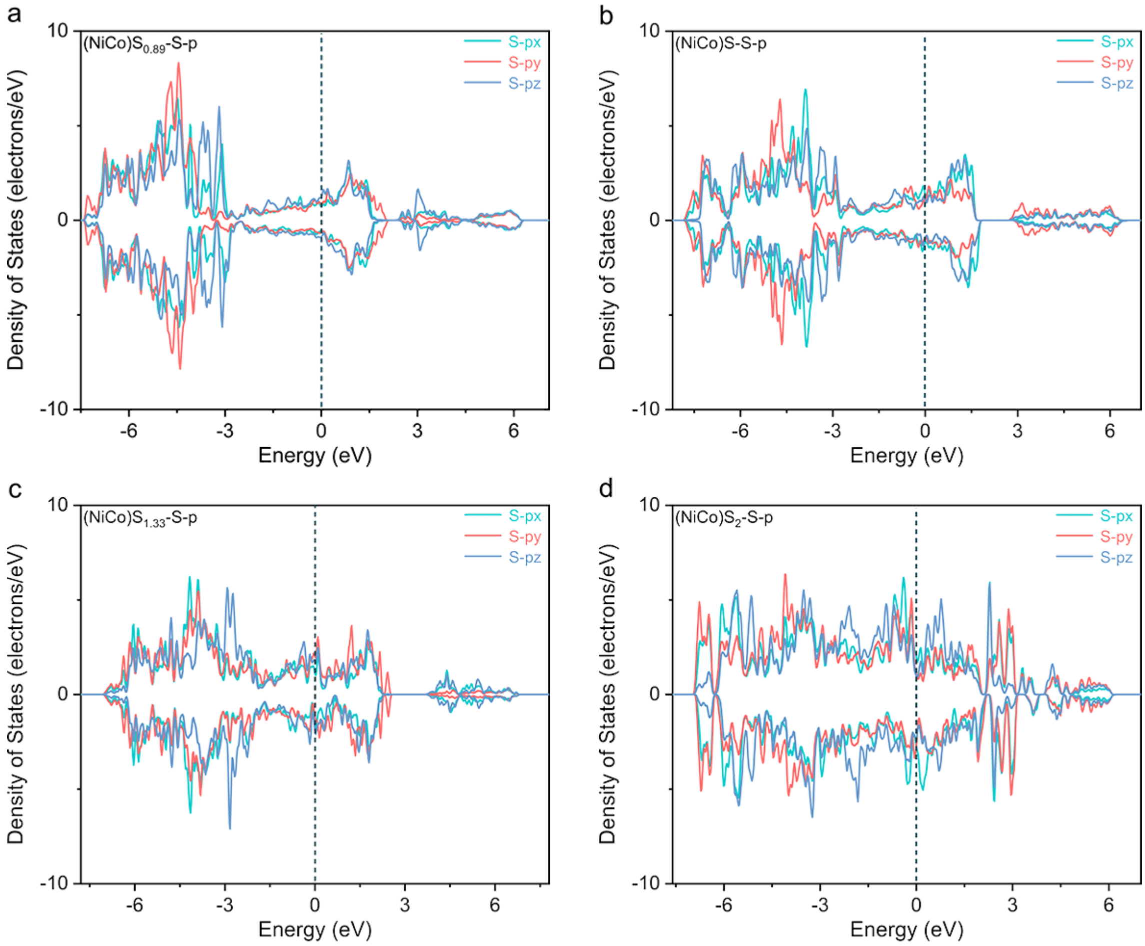

Supplementary Fig. 48 | The calculation of PDOS-S in sulphides. a-d, PDOS-S of $(NiCo)S_{0.89}$, $(NiCo)S$, $(NiCo)S_{1.33}$ and $(NiCo)S_2$, respectively.

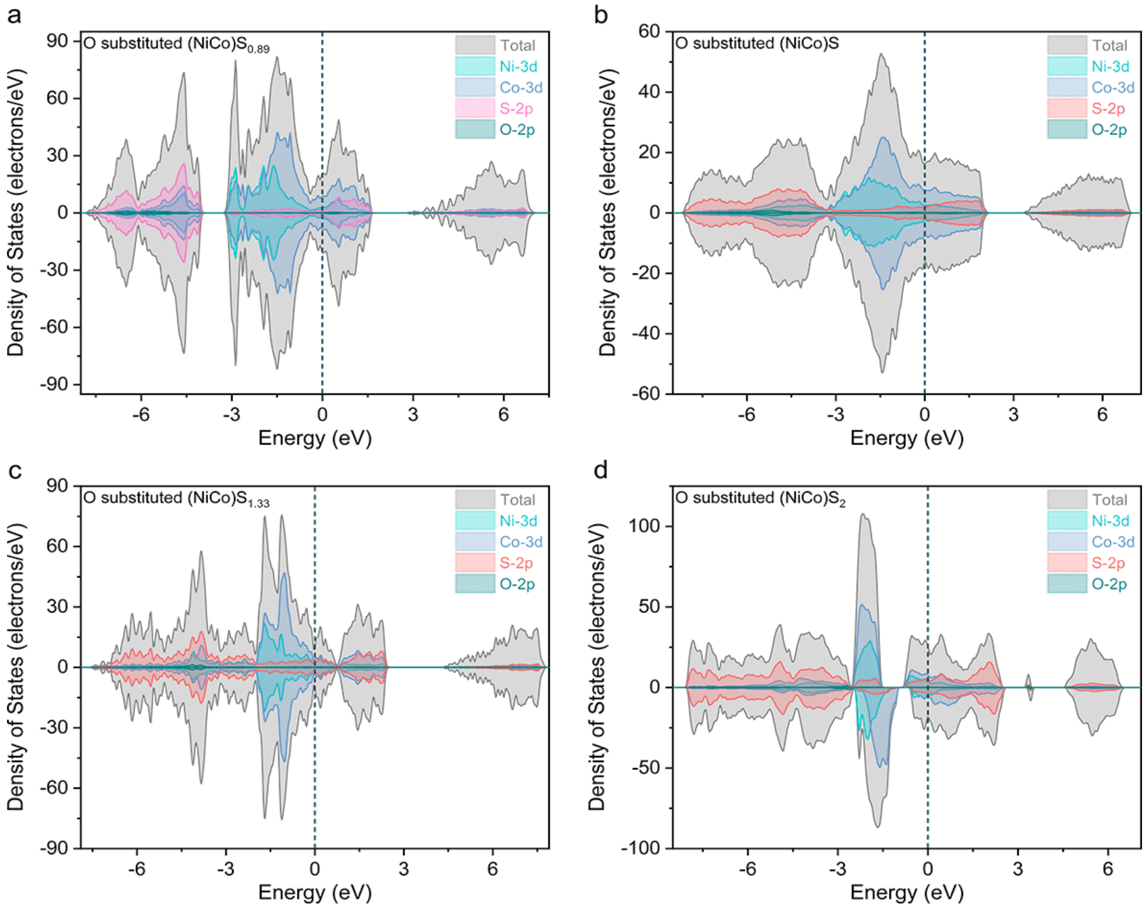

Supplementary Fig. 49 | The calculation of PDOS in O-substituted sulphides. a-d, PDOS of O-substituted $(NiCo)S_{0.89}$, $(NiCo)S$, $(NiCo)S_{1.33}$ and $(NiCo)S_2$, respectively.

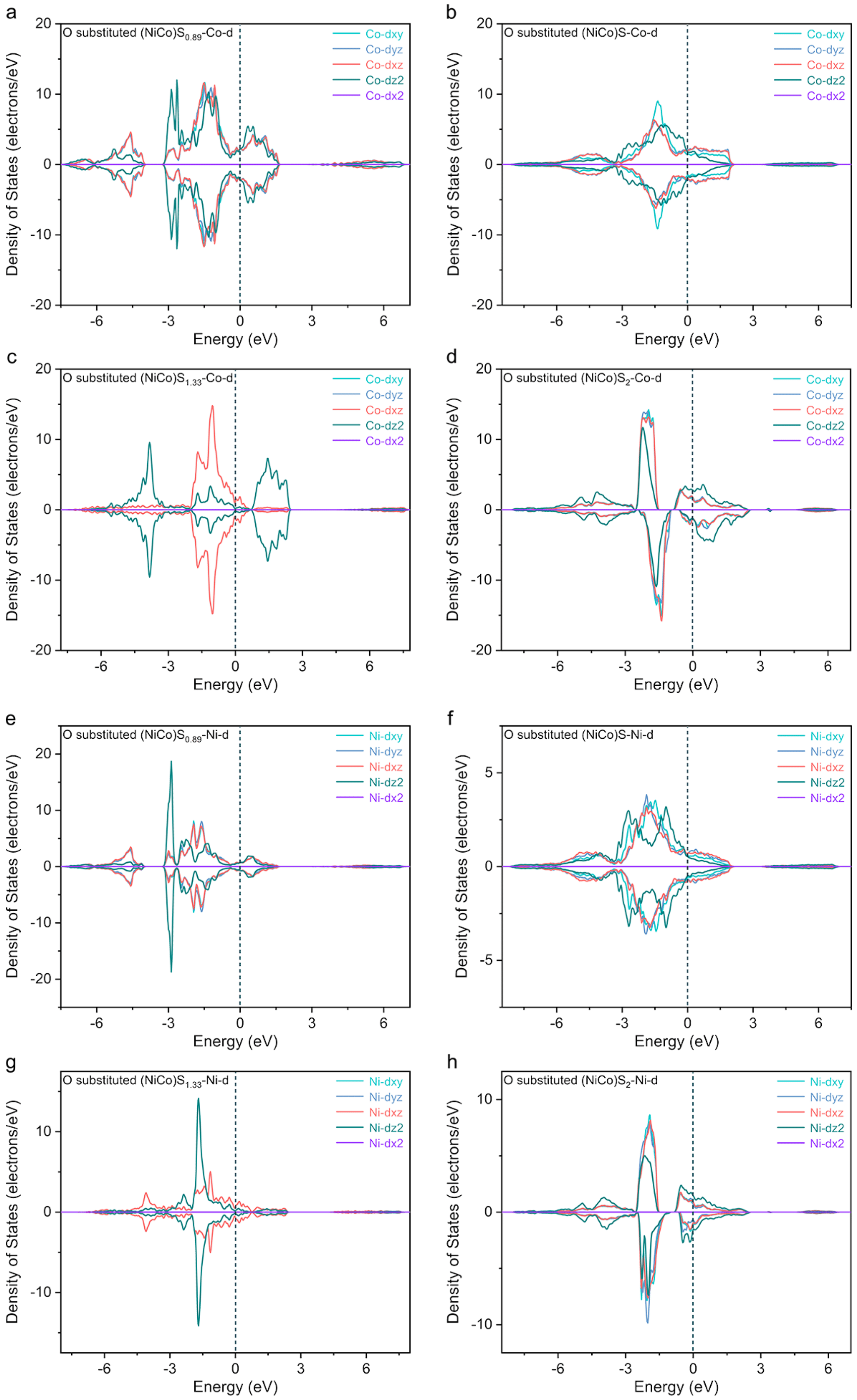

Supplementary Fig. 50 | The calculation of PDOS-Co and Ni in O-substituted sulphides. a-d, PDOS-Co of O-substituted (NiCo)$S_{0.89}$, (NiCo)S, (NiCo)$S_{1.33}$ and (NiCo)S_2, respectively. **e-h**, PDOS-Ni of O-substituted (NiCo)$S_{0.89}$, (NiCo)S, (NiCo)$S_{1.33}$ and (NiCo)S_2, respectively.

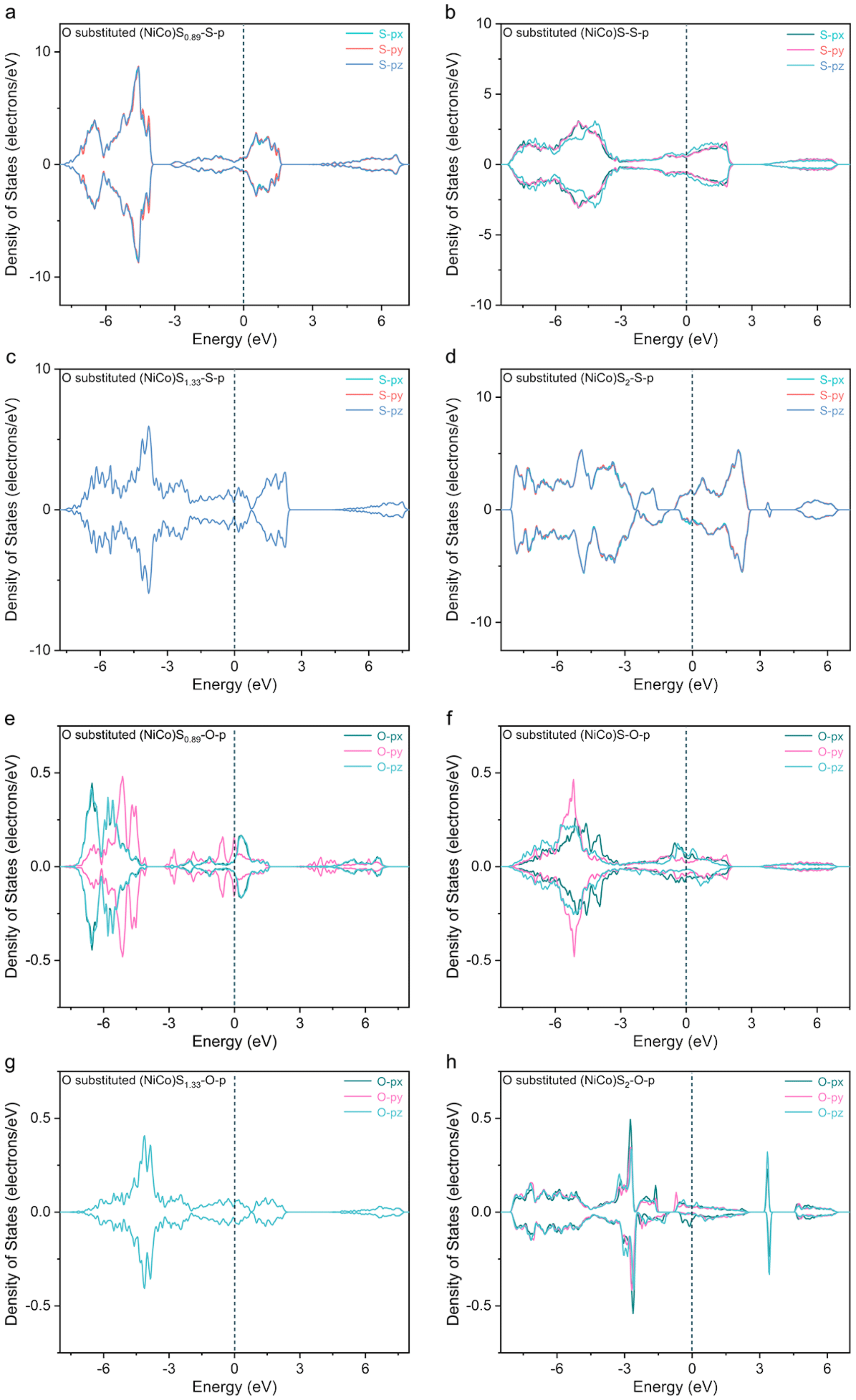

Supplementary Fig. 51 | The calculation of PDOS-S and O in O-substituted sulphides. a-d, PDOS-S of O-substituted $(NiCo)S_{0.89}$, (NiCo)S, $(NiCo)S_{1.33}$ and $(NiCo)S_2$, respectively. **e-h**, PDOS-O of O-substituted $(NiCo)S_{0.89}$, (NiCo)S, $(NiCo)S_{1.33}$ and $(NiCo)S_2$, respectively.

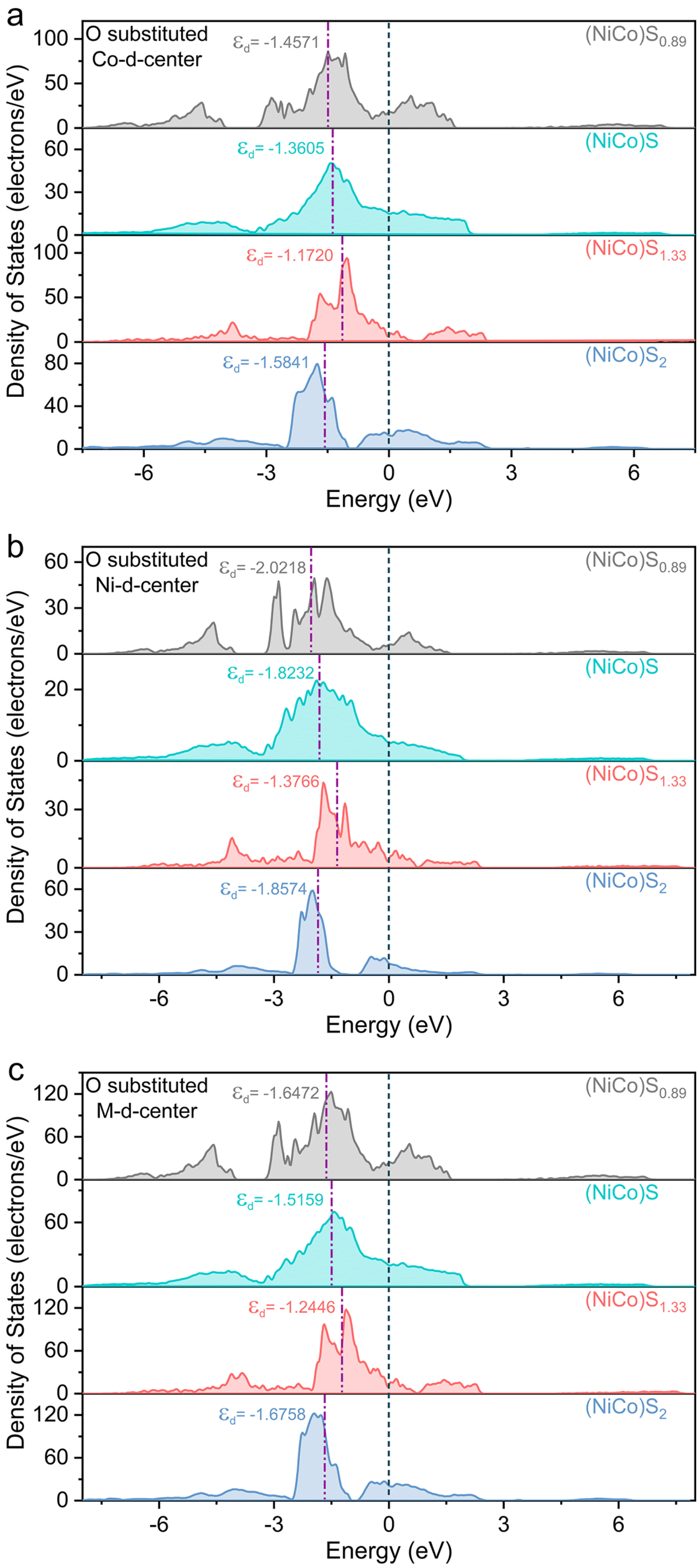

Supplementary Fig. 52 | The calculation of d-band center in O-substituted sulphides. a-c, d-band center of Co, Ni, Metal in O-substituted sulphides, respectively.

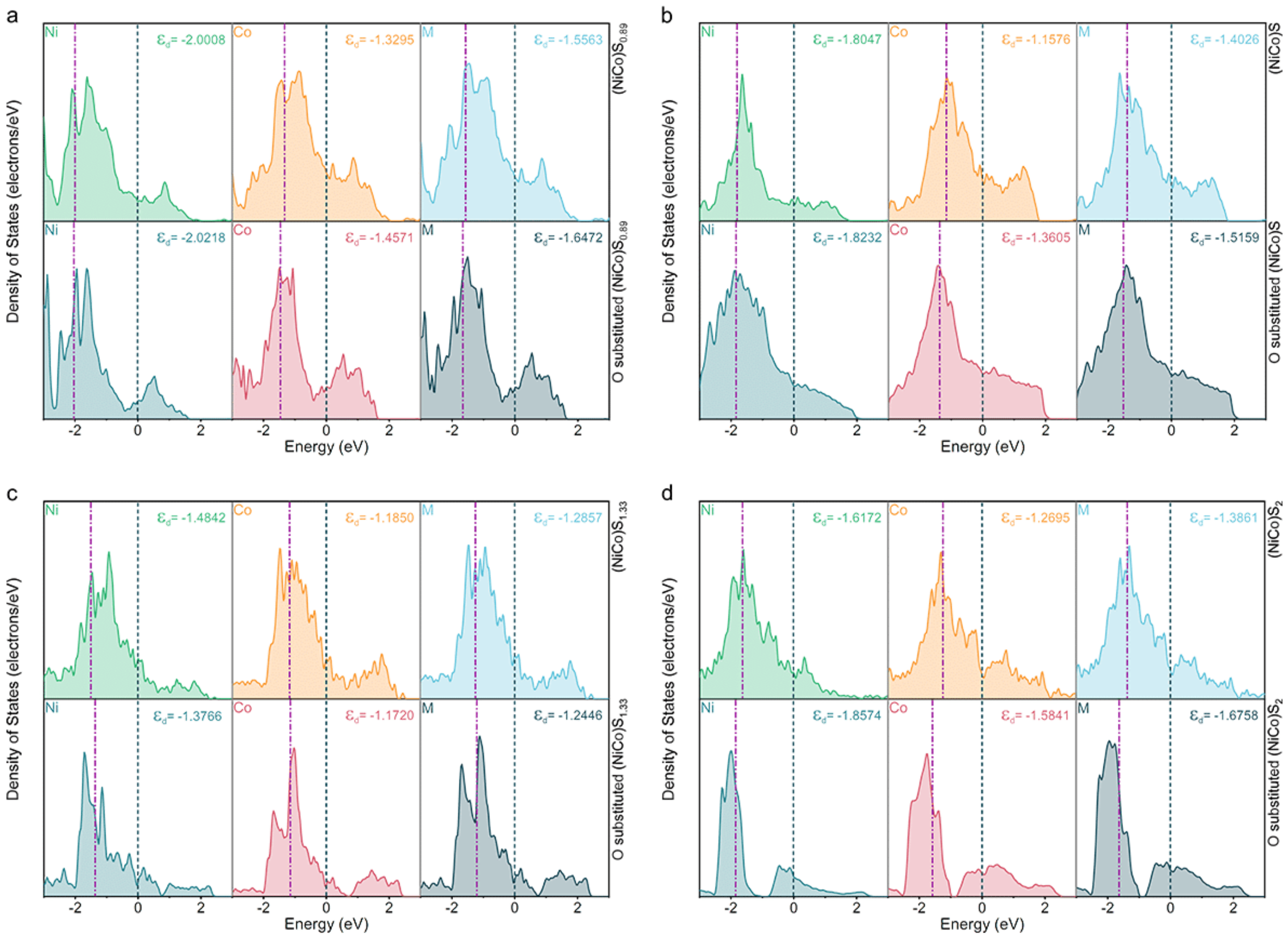

Supplementary Fig. 53 | The comparison of d-band center in pure and O-substituted sulphides. a-d, Ni, Co and Metal d-band centre of pure and O-substituted $(NiCo)S_{0.89}$, (NiCo)S, $(NiCo)S_{1.33}$ and $(NiCo)S_2$, respectively.

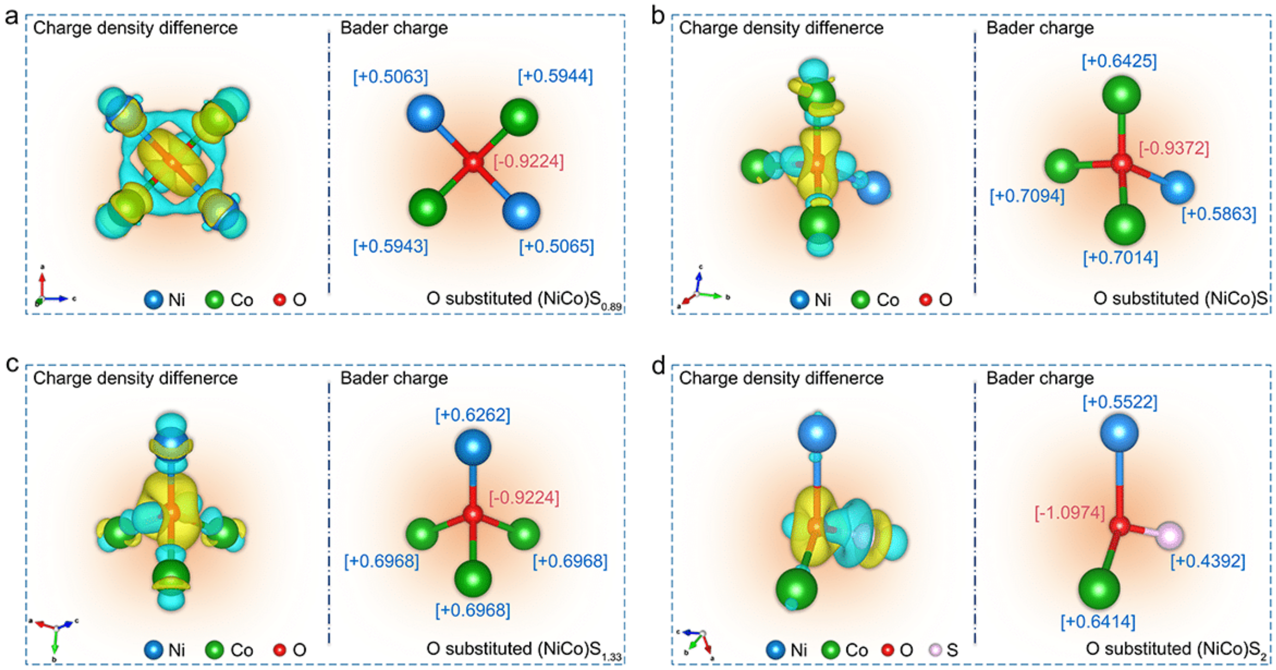

Supplementary Fig. 54 | The calculation of Bader charge and charge density difference. a-d, Bader charge and charge density difference of O-substituted $(NiCo)S_{0.89}$, (NiCo)S, $(NiCo)S_{1.33}$ and $(NiCo)S_2$, respectively.

Bard charge and charge density difference also lead to the same conclusion of enhanced adsorption to reaction intermediates with the d-band center results.

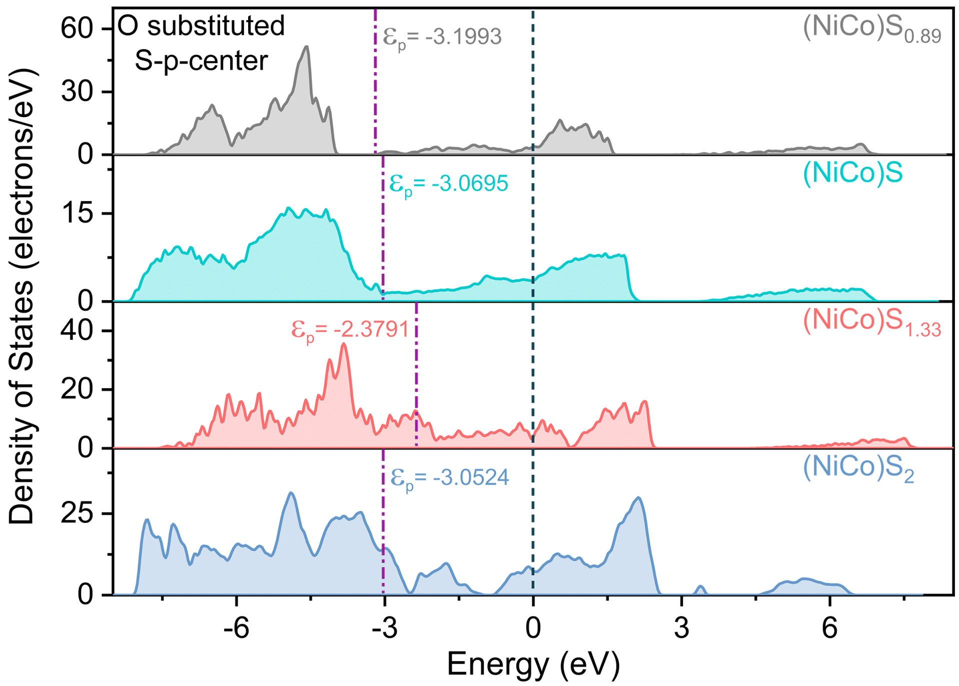

Supplementary Fig. 55 | S p-band centre of O-substituted $(NiCo)S_{0.89}$, (NiCo)S, $(NiCo)S_{1.33}$ and $(NiCo)S_2$.

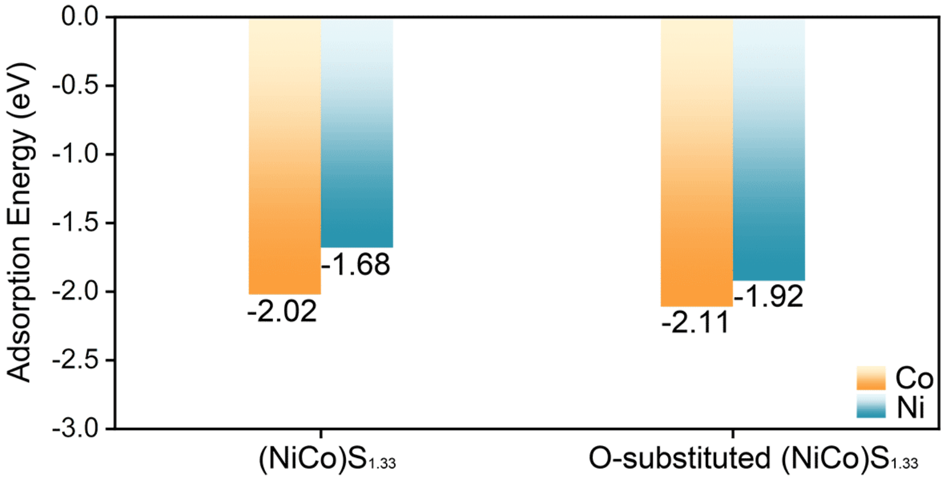

Supplementary Fig. 56 | The calculation of OH* adsorption energy on catalytically active sites. Comparison of OH* adsorption energy on catalytically active Co and Ni sites on (110) terminated surfaces of $(NiCo)S_{1.33}$ and O-substituted $(NiCo)S_{1.33}$.

The higher OH* adsorption energy indicates more preferential attachment on the active site. As shown in Supplementary Fig. 56, the adsorption energy of OH* on Co sites on (110) terminated surfaces of $(NiCo)S_{1.33}$ and O-substituted $(NiCo)S_{1.33}$ were calculated to be -2.02 and -2.11 eV, respectively. In comparison, the adsorption energy of OH* on Ni sites of both $(NiCo)S_{1.33}$ and O-substituted $(NiCo)S_{1.33}$ were lower than that on Co sites, which suggested that Co is the dominated active sites. Besides, the adsorption energy of OH* on Ni sites was revealed to lift more obvious than that of Co, which implied the enhancement of OH* adsorption capacity of Ni.

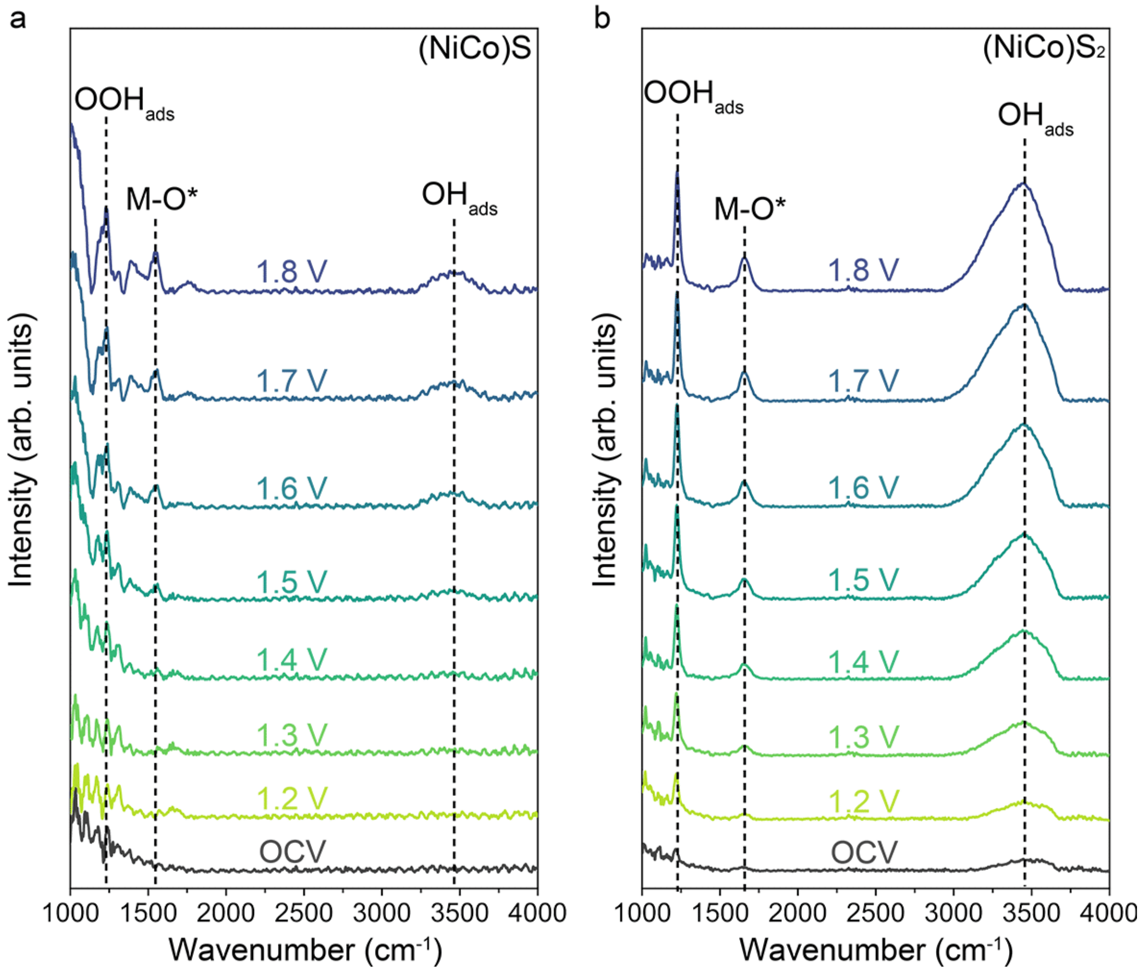

Supplementary Fig. 57 | in-situ FT-IR spectra of (NiCo)S and (NiCo)S_2 recorded during the multi-potential steps. a, in-situ FT-IR spectra of (NiCo)S. **b**, in-situ FT-IR spectra of (NiCo)S_2.

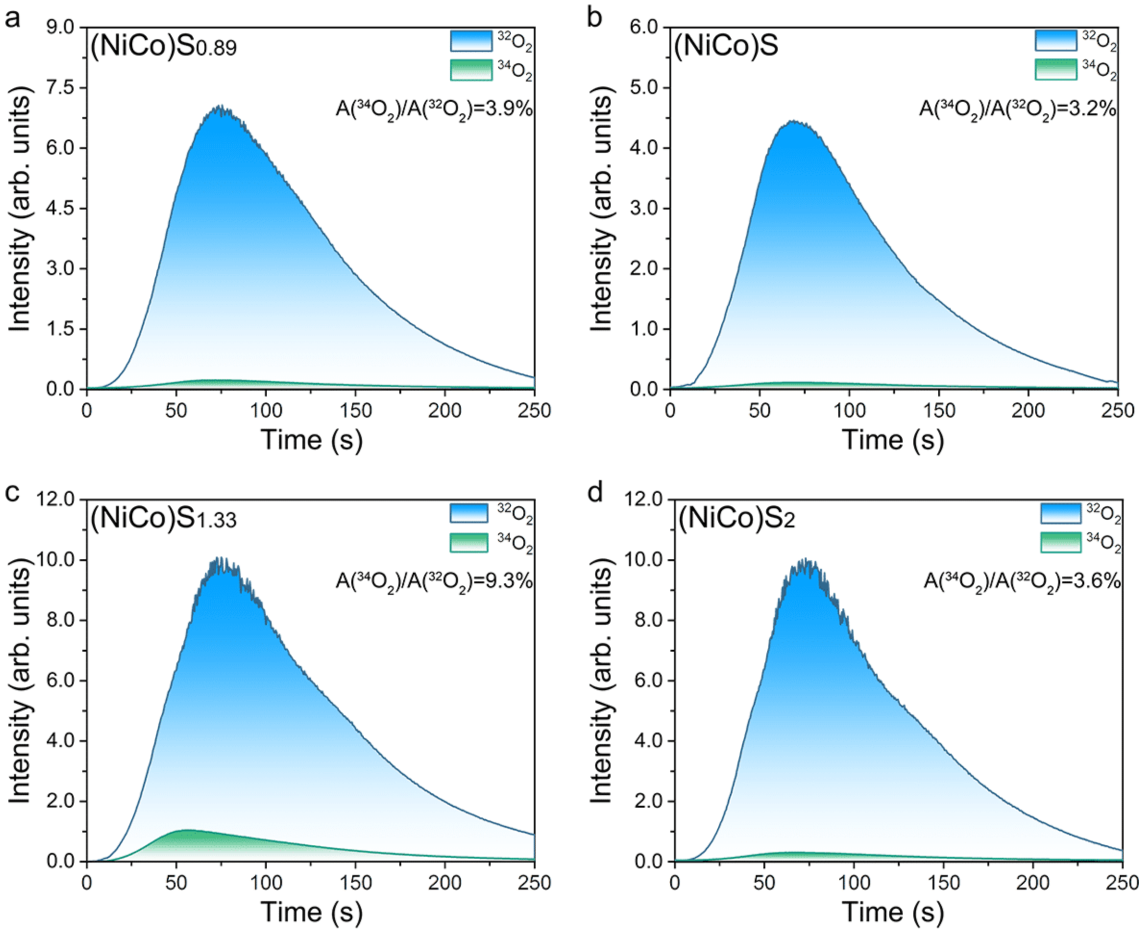

Supplementary Fig. 58 | DEMS signals of $^{34}O_2$ and $^{32}O_2$ of $(NiCo)S_{0.89}$, (NiCo)S, $(NiCo)S_{1.33}$ and $(NiCo)S_2$ from the reaction products cycled in $H_2{}^{16}O$ aqueous KOH electrolyte. a, DEMS signals of $(NiCo)S_{0.89}$. **b**, DEMS signals of (NiCo)S. **c**, DEMS signals of $(NiCo)S_{1.33}$. **d**, DEMS signals of $(NiCo)S_2$.

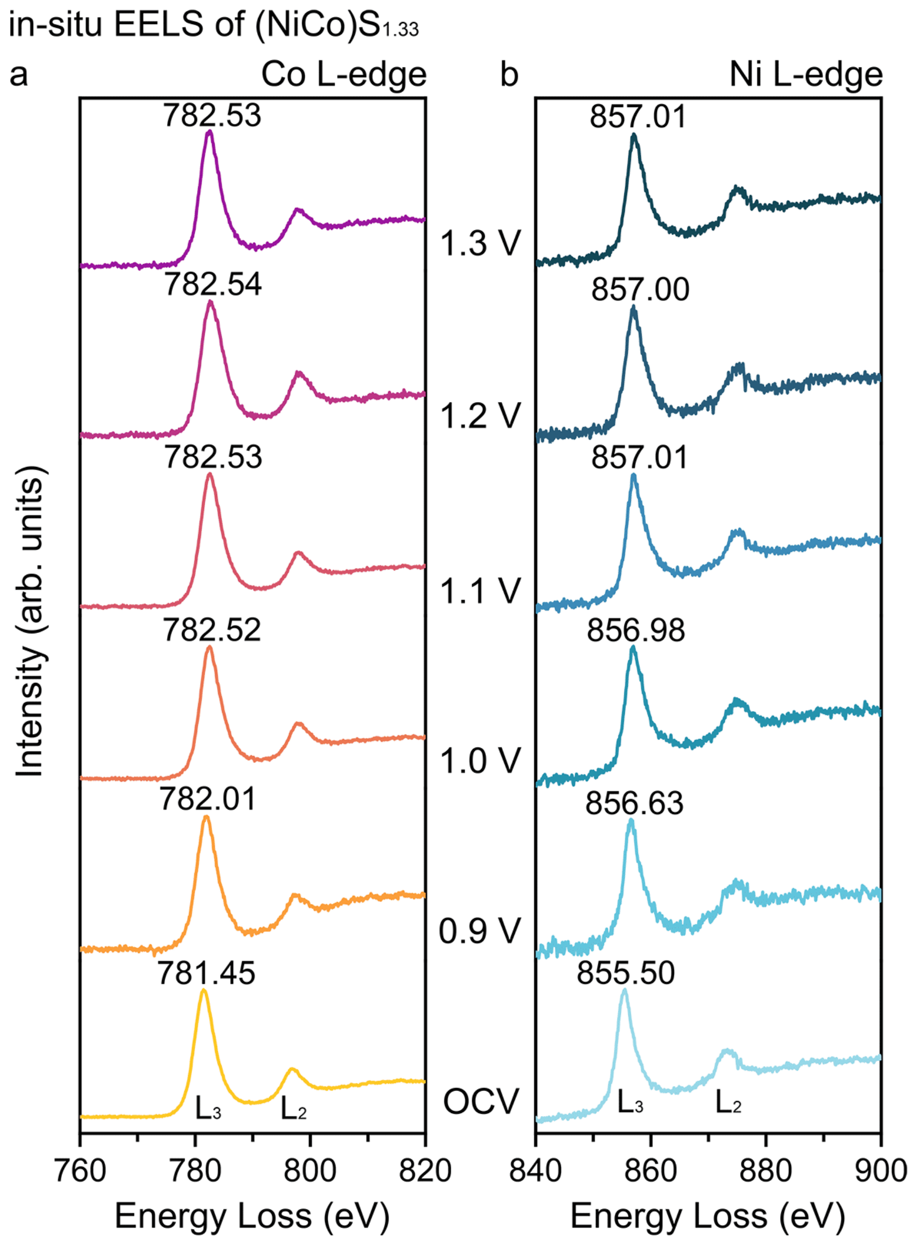

Supplementary Fig. 59 | in-situ EELS spectra of Co and Ni L-edge of $(NiCo)S_{1.33}$ under different applied potential. a, in-situ EELS spectrum of Co L-edge. **b**, in-situ EELS spectrum of Ni L-edge.

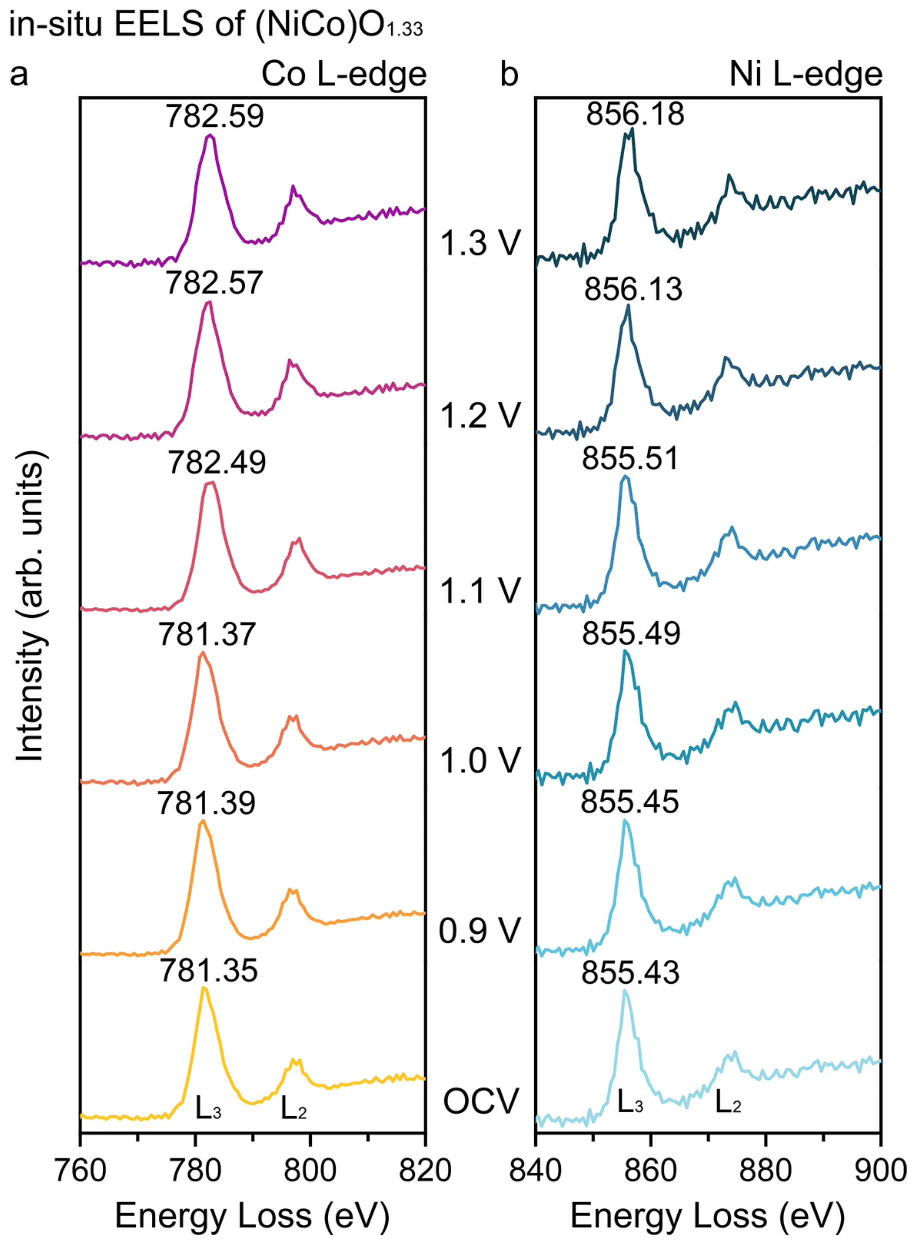

Supplementary Fig. 60 | in-situ EELS spectrum of Co and Ni L-edge of $(NiCo)O_{1.33}$ under different applied potential. a, in-situ EELS spectrum of Co L-edge. **b**, in-situ EELS spectrum of Ni L-edge.

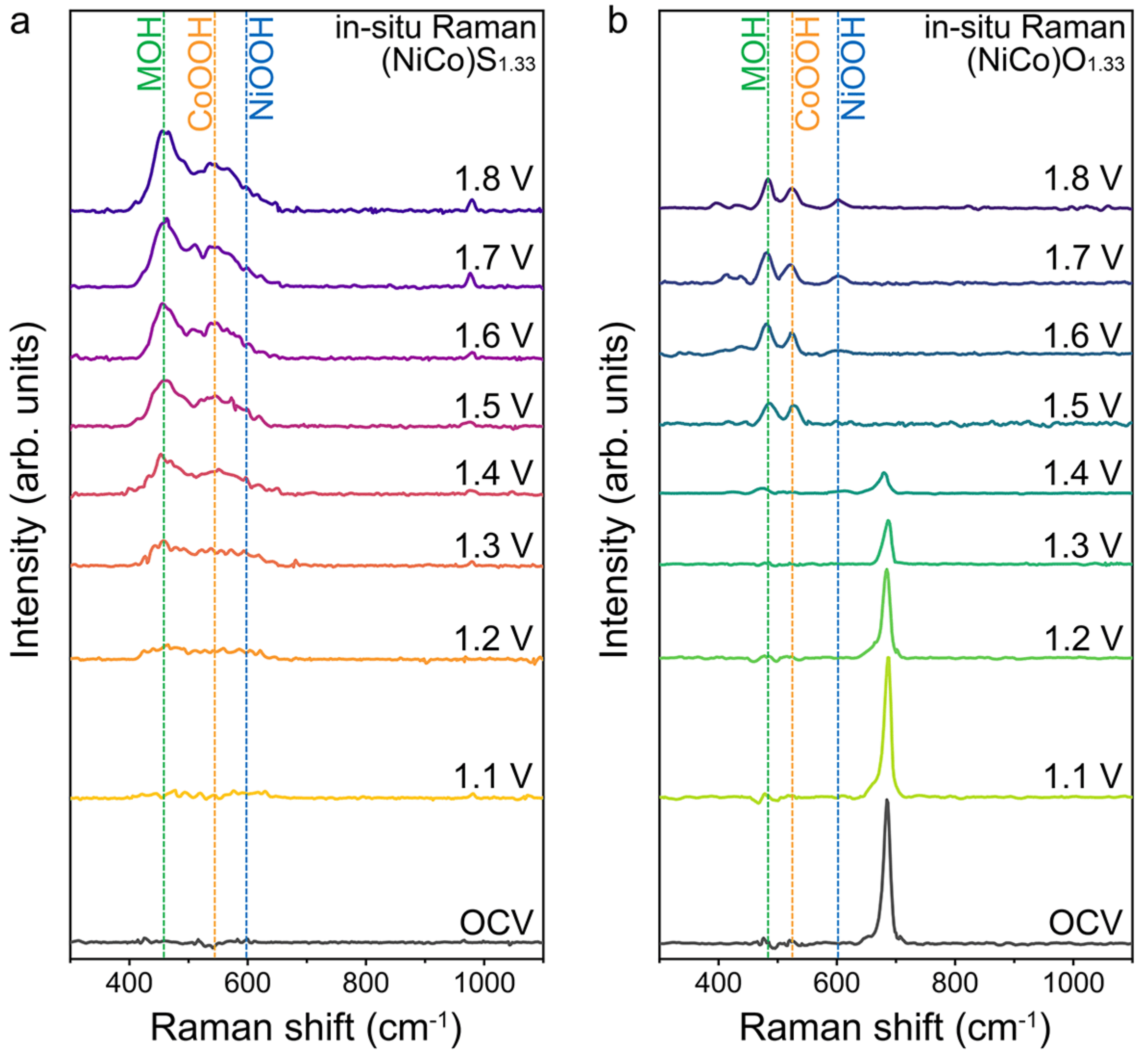

Supplementary Fig. 61 | in-situ Raman spectra of the (NiCo)$S_{1.33}$ and (NiCo)$O_{1.33}$ at various constant applied potentials. a, in-situ Raman spectra of (NiCo)$S_{1.33}$. **b**, in-situ Raman spectra of (NiCo)$S_{1.33}$.

The operando Raman spectra of the (NiCo)$S_{1.33}$ and (NiCo)$O_{1.33}$ was taken at different applied potentials, the pronounced peaks at approximately 462, 544, and 600 cm^{-1} could be ascribed to Co or Ni-OH, Co-OOH and Ni-OOH, respectively.

Supplementary Table 1 | The d-electron number, total d-electron energy and ε_d of metal atoms in $(NiCo)S_{0.89}$, $(NiCo)S$, $(NiCo)S_{1.33}$ and $(NiCo)S_2$.

		**Co**	**Ni**	**M**
$(NiCo)S_{0.89}$	d-electron number	181.822	92.7265	274.5485
	Total d-electron energy	-241.7314	-185.5358	-427.2673
	ε_d	- 1.3295	- 2.0008	- 1.5563
$(NiCo)S$	d-electron number	152.6416	92.9857	245.6273
	Total d-electron energy	-176.7053	-167.8072	-344.5125
	ε_d	- 1.1576	- 1.8047	- 1.4026
$(NiCo)S_{1.33}$	d-electron number	121.5021	61.6732	183.1753
	Total d-electron energy	-143.9777	-91.5377	-235.5154
	ε_d	- 1.1850	- 1.4842	- 1.2857
$(NiCo)S_2$	d-electron number	121.5398	61.3125	182.8523
	Total d-electron energy	-154.2961	-99.1551	-253.4513
	ε_d	- 1.2695	- 1.6172	- 1.3861

Supplementary Table 2 | The atomic fraction of elements in $(NiCo)S_{1.33}$ under different applied voltage. The atomic fraction of Co, Ni, S and O were extracted from elemental mapping data.

**$(NiCo)S_{1.33}$**	**Co**	**Ni**	**S**	**O**
OCV	24.74	13.81	57.93	3.52
0.9 V	28.28	14.02	30.72	26.98
1.0 V	32.81	14.67	6.32	46.20
1.1 V	34.53	13.94	4.28	47.25
1.2 V	33.86	14.22	3.29	48.63
1.3 V	33.32	14.85	3.25	48.58

Supplementary Table 3 | The atomic fraction of elements in $(NiCo)S_{0.89}$, (NiCo)S and $(NiCo)S_2$ under different applied voltage. The atomic fraction of Co, Ni, S and O were extracted from elemental mapping data.

**$(NiCo)S_{0.89}$**	**Co**	**Ni**	**S**	**O**
OCV	30.12	12.35	52.88	4.65
0.9 V	29.21	12.68	50.67	7.44
1.0 V	28.54	13.01	49.33	9.12
1.1 V	31.38	11.06	45.25	12.31
1.2 V	35.00	12.55	8.93	43.52
1.3 V	36.11	12.79	6.94	44.16
**(NiCo)S**				
OCV	29.86	16.78	50.12	3.24
0.9 V	29.33	22.24	44.85	3.58
1.0 V	28.19	23.61	44.19	4.01
1.1 V	28.65	23.45	43.68	4.22
1.2 V	29.01	23.50	42.51	4.98
1.3 V	32.43	23.54	4.15	39.88
**$(NiCo)S_2$**				
OCV	31.25	11.03	55.39	2.33
0.9 V	30.87	11.16	53.76	4.21
1.0 V	29.98	10.85	53.22	5.95
1.1 V	30.11	10.52	49.23	10.14
1.2 V	32.55	18.34	9.22	39.89
1.3 V	33.08	14.28	8.87	43.77

Supplementary Table 4 | The atomic fraction of elements in $(NiCo)S_{1.33}$ under different applied voltage. The atomic fraction of Co, Ni, S and O were extracted from elemental mapping data.

**$(NiCo)S_{1.33}$**	**Co**	**Ni**	**S**	**O**
0.50 V	28.09	12.16	55.23	4.52
0.60 V	27.74	13.01	54.15	5.10
0.70 V	27.55	12.57	54.93	4.95
0.80 V	27.42	12.85	55.01	4.72
0.90 V (10 s)	33.86	13.13	43.28	16.08

Supplementary Table 5 | The p-electron number, total p-electron energy and ε_p of sulphide atoms in $(NiCo)S_{0.89}$, $(NiCo)S$, $(NiCo)S_{1.33}$ and $(NiCo)S_2$.

	**p-electron number**	**Total p-electron energy**	**ε_p**
$(NiCo)S_{0.89}$	95.3909	-301.3832	- 3.1595
$(NiCo)S$	96.8745	-274.5534	- 2.8341
$(NiCo)S_{1.33}$	97.4003	-222.0939	- 2.2802
$(NiCo)S_2$	118.8855	-299.6440	- 2.5339

Supplementary Table 6 | Calculated lattice parameters a, b, and c (Å), cell volume per atom V (Å^3), formation enthalpy per atom ΔH (eV) and Energy of $(NiCo)S_{0.89}$, (NiCo)S, $(NiCo)S_{1.33}$ and $(NiCo)S_2$.

	a (Å)	b (Å)	c (Å)	V (Å3)	*ΔH*	Energy
O-$(NiCo)S_{0.89}$	9.927	9.927	9.927	14.386	-0.7325	-422.449
O-(NiCo)S	10.131	6.754	10.300	12.716	-0.6503	-290.134
O-$(NiCo)S_{1.33}$	9.325	9.325	9.325	14.481	-0.8334	-336.061
O-$(NiCo)S_2$	16.604	11.070	5.535	14.129	-0.7760	-406.216

Supplementary Table 7 | The d-electron number, total d-electron energy and ε_d of metal atoms in O-substituted $(NiCo)S_{0.89}$, (NiCo)S, $(NiCo)S_{1.33}$ and $(NiCo)S_2$.

		Co	Ni	M
$O\text{-}(NiCo)S_{0.89}$	d-electron number	185.2171	93.9619	279.1790
	Total d-electron energy	-269.8741	-189.9767	-459.8509
	ε_d	-1.4571	-2.0218	-1.6472
O-(NiCo)S	d-electron number	123.2872	62.3456	185.6328
	Total d-electron energy	-167.7325	-113.6666	-281.3991
	ε_d	-1.3605	-1.8232	-1.5159
$O\text{-}(NiCo)S_{1.33}$	d-electron number	123.1786	67.7670	190.9456
	Total d-electron energy	-144.3656	-93.2866	-237.6522
	ε_d	-1.1720	-1.3766	-1.2446
$O\text{-}(NiCo)S_2$	d-electron number	122.5278	61.8475	184.3753
	Total d-electron energy	-194.0918	-114.8767	-308.9683
	ε_d	-1.5841	-1.8574	-1.6758

Supplementary Table 8 | The p-electron number, total p-electron energy and ε_p of sulphide atoms in O-substituted $(NiCo)S_{0.89}$, $(NiCo)S$, $(NiCo)S_{1.33}$ and $(NiCo)S_2$.

	**p-electron number**	**Total p-electron energy**	**ε_p**
O-$(NiCo)S_{0.89}$	95.1406	-304.3796	-3.1993
O-(NiCo)S	71.1632	-218.4335	-3.0695
O-$(NiCo)S_{1.33}$	96.4874	-229.5491	-2.3791
O-$(NiCo)S_2$	148.7318	-453.9879	-3.0524

References

1. Kresse, G. Ab initio molecular dynamics for liquid metals. *Phys. Rev. B* **47**, 558 (1993).
2. Kresse, G. et al. Efficient iterative schemes for ab initio total-energy calculations using a plane-wave basis set. *Phys. Rev. B* **54**, 11169-11186 (1996).
3. Perdew, J.P. et al. Generalized gradient approximation made simple. *Phys. Rev. Lett.* **77**, 3865 (1996).
4. Blöchl, P.E. Projector augmented-wave method. *Phys. Rev. B.* **50**, 17953-17979 (1994).
5. Grimme, S. et al. A consistent and accurate ab initio parametrization of density functional dispersion correction (DFT-D) for the 94 elements H-Pu. *J. Chem. Phys.* **132**, 154104 (2010).
