# Supplementary material for: Understanding the sulphur-oxygen exchange process of metal sulphides prior to oxygen evolution reaction
Source: Nat Commun. 2023 Apr 7;14:1949. doi: 10.1038/s41467-023-37751-y (PMC10082196; doi:10.1038/s41467-023-37751-y)
Supplement: Supplementary file 3 — Description of Additional Supplementary Files [file 41467_2023_37751_MOESM3_ESM.docx]

**Description of Additional Supplementary Files**

File name: Supplementary Movie

Description: Real-time observation of the surface reconstruction process of (NiCo)S_1.33_ particles under the applied voltage of 0.9 V (vs Pt pseudo-Reference).

File name: Source Data

Description: The source data of figures in the main text and Supplementary Information.
